# Discovery and enantiocontrol of axially chiral urazoles via organocatalytic tyrosine click reaction

**DOI:** 10.1038/ncomms10677

**Published:** 2016-02-11

**Authors:** Ji-Wei Zhang, Jin-Hui Xu, Dao-Juan Cheng, Chuan Shi, Xin-Yuan Liu, Bin Tan

**Affiliations:** 1 Department of Chemistry, South University of Science and Technology of China, Shenzhen 518055, China

## Abstract

Axially chiral compounds play an important role in areas such as asymmetric catalysis. The tyrosine click-like reaction is an efficient approach for synthesis of urazoles with potential applications in pharmaceutical and asymmetric catalysis. Here we discover a class of urazole with axial chirality by restricted rotation around an N–Ar bond. By using bifunctional organocatalyst, we successfully develop an organocatalytic asymmetric tyrosine click-like reaction in high yields with excellent enantioselectivity under mild reaction conditions. The excellent remote enantiocontrol of the strategy originates from the efficient discrimination of the two reactive sites in the triazoledione and transferring the stereochemical information of the catalyst into the axial chirality of urazoles at the remote position far from the reactive site.

## Abstract (web summary)

Axially chiral compounds play an important role in areas such as asymmetric catalysis. Here, the authors report an organocatalytic asymmetric tyrosine click-like reaction, giving access to enantio enriched urazoles with restricted rotation around an N-C bond.

## Graphical abstract

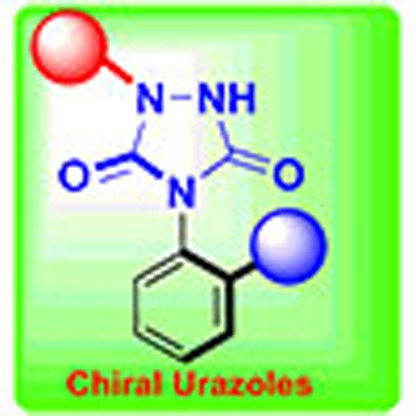

Urazoles are important heterocyclic compounds with potential pharmaceutical applications and valuable utilities in the area of protein modification chemistry due to the simplicity of chemical synthesis and ease of optimization of reaction conditions^1^,^2^,^3^,^4^. In addition, oxidation of urazoles gives rise to a very useful class of persistent cyclic hydrazyl radicals for versatile transformations^5^,^6^. Consequently, there is a large demand for easy access to a broad variety of these compounds. In this regard, the tyrosine click reaction provides a straightforward strategy to access such compounds under mild conditions as illustrated in Fig. 1a, in which a class of cyclic diazodicarboxamides (triazodiones) reacted selectively and rapidly with the phenol side chain of tyrosine as first developed by the Barbas group for the application in bioconjugate chemistry^7^,^8^. Although the development of other methodologies towards the synthesis of these compounds has also been reported^9^,^10^,^11^, to the best of our knowledge, there is no any report involving the direct construction of chiral urazoles in a catalytic enantioselective manner. Inspired by a developing research field on atropisomeric compounds possessing an N–Ar chiral axis^12^, we envisioned that urazoles directly obtained from tyrosine click-like reaction could be recognized as a type of axially chiral skeleton containing an N–Ar chiral axis because of the presence of two N–Ar bonds in arylurazoles.

After discovery of the axially chiral urazoles (Fig. 1b, compound **D**), we turned our attention to construct the chiral urazoles in an atroposelective approach via tyrosine click-like reaction. In this scenario, three major challenges would be encountered: (1) the selection of suitable catalyst to interact with the substrates in high efficiency to inhibit the very strong background reaction; (2) the choice of an appropriate chiral catalyst prompt to efficiently induce remote axial enantiocontrol at the distant position via organocatalytic desymmetrization strategy^13^,^14^,^15^,^16^,^17^,^18^,^19^; (3) the use of mild reaction conditions to circumvent the axial rotation. Recently, some strategies have been successfully developed for the organocatalytic synthesis of axially chiral compounds^20^,^21^,^22^,^23^,^24^,^25^,^26^,^27^,^28^,^29^,^30^,^31^,^32^,^33^. Although the task of controlling the remote axial chirality under the current reaction system is a formidable challenge, the success of the above results provides strong evidence that organocatalysis can be performed in the control of axial chirality by using rationally designed substrate or catalyst. It is well known that bifunctional organocatalysts have made a great contribution to the field of asymmetric catalysis^34^,^35^,^36^,^37^. In such catalysts, the acidic and basic centres acting as both hydrogen-bonding donors and acceptors, respectively, thus activating the nucleophile and electrophile at the same time in an appropriate spatial configuration. As shown in Fig. 1c, we speculated that the utility of bifunctional organocatalysts could be expected by distinguishing the two nonequivalent reactive nitrogen centres (*a* and *b*) in the triazoledione and transferring the central chirality of the catalyst into the axial chirality far from the reaction site. As part of our continued interest in the area of synthesis of axially chiral compounds^38^ and asymmetric catalysis^39^, herein, we would like to exhibit the remote control of the axial chirality of arylurazoles by using a desymmetrization strategy via organocatalytic tyrosine click reaction of 4-aryl-1,2,4-triazole-3,5-dione (ATAD). The key feature of our strategy is the ability of a bifunctional organocatalyst to transfer its stereochemical information to a remote position and thereafter efficiently control its axial chirality.

## Results

### Discovery of urazoles with axial chirality

**Figure 1:**
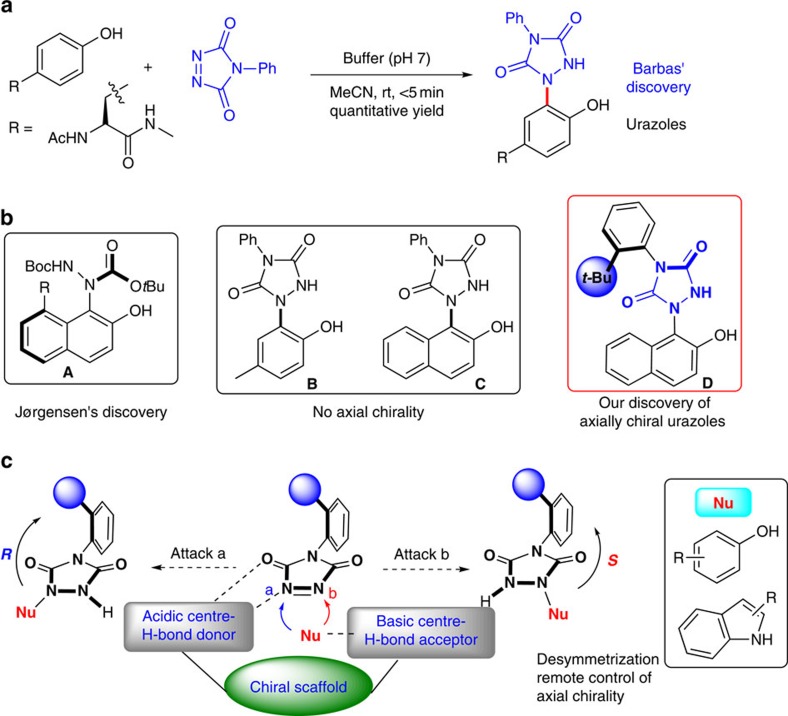
Synthesis of urazoles via tyrosine click reaction and discovery of axial chirality and strategy for remote enantiocontrol. (**a**) Synthesis of urazoles via tyrosine click reaction (Barbas' discovery). (**b**) Discovery of urazoles with axial chirality. (**c**) Our strategy for remote enantiocontrol of axial chirality of urazoles.

In 2006, the Jørgensen group discovered a new class of axially chiral skeleton **A** via asymmetric amination of 8-amine-2-naphthol with azodicarboxylates (Fig. 1b)^40^,^41^. Motivated by this pioneering discovery, we synthesized the compounds **B** and **C** through tyrosine click reaction and imagined that such compounds should have axial chirality due to the significant restricted rotation between nitrogen atom and the directly attached phenol ring or naphthol ring (Fig. 1b). Disappointedly, they did not display axial chirality based on the chiral stationary high-performance liquid chromatography (HPLC) analysis presumably because of the relatively low rotational barrier of the N–Ar bond. To further screen different aryl substituents of triazodiones, we are pleased to find that urazole **D** with a steric bulky substituent (*t*-butyl group) in the *ortho* position of the phenyl ring shows apparently axial chirality. As such, a class of urazoles with axial chirality was discovered (Fig. 1b).

### Optimization of reaction conditions involving naphthols

To investigate the feasibility of our hypothesis, we initiated to conduct the tyrosine click reaction of naphthol (**1a**) with 4-(2-*tert*-butylphenyl)-3*H*-1,2,4-triazole-3,5-dione (**2a**) by using Takemoto catalyst (**C1**)^42^ in dichloromethane (DCM) at room temperature. To our delight, the desired product **3a** was obtained in almost quantitative yield in less than 5 min, albeit without any enantioselectivity. Using the analysis of chiral HPLC, the urazole compound **3a** was confirmed to be atropisomeric and two peaks corresponding to the enantiomers were observed on the chiral HPLC at room temperature without any change during the analysis timescale. In the absence of organocatalyst, the reaction also proceeded very smoothly (less than 5 min for the model reaction) in quantitative yield, indicating that the strong background reaction might be the major challenge for efficiently realizing enantioselective transformation. With these initial results in hand and to improve the enantioselectivity, we turned our attention to decrease the reaction temperature to −78 °C. Gratifyingly, the reaction proceeded completely within just 5 min and the desired product was obtained in 57% isolated yield with 25% enantioselectivity excess (ee). We next investigated different bifunctional thiourea-tertiary amine catalysts (Table 1, entries 2–5). Among the tested catalysts, Takemoto catalyst **C3** with a cyclic tertiary amine proved to be very promising, with the ee value up to 75%. Considering that the additional aromatic stacking interaction might be involved in the transition states, catalysts **C6** and **C7** with an axial binaphthyl moiety were tested^43^. Catalyst **C7** displayed an excellent enantiocontrol (entry 7), while catalyst **C6** with opposite configuration of diamine gave rise to poor enantioselectivity (entry 6). As shown in entry 8, the diamine skeleton in the catalyst had a great influence on the asymmetric induction. Of the solvents tested for the reaction catalysed by **C7**, diethyl ether proved optimal with respect to the enantioselectivity (Table 1, entry 10). It is noteworthy that the reaction proceeded smoothly without having any affect on enantioselectivity (99% ee) and with an improved chemical yield up to 82% when 5 mol% of catalyst was used (entry 11).

### Substrate scope

After the optimal reaction condition being established, we set out to explore the substrate scope with respect to various phenols and 2-naphthnols as reactants (Table 2). All of the investigated reactions were complete within 60 min and gave products in moderate to good yields (51–85%) and with excellent enantioselectivities (90–99% ee). As regarding the use of a variety of 2-naphthols, bearing electron-withdrawing (Table 2, products **3b**–**3f**) and electron-donating (Table 2, products **3g**–**3h**) groups, the reaction of these 2-naphthols with **2a** gave the expected products with very high stereoselectivities. These results indicated that there was only limited influence on stereoselectivity regardless of the electronic properties of the substituents at the different positions on the aromatic ring. It is noteworthy that the use of 4-substituted phenol, such as 4-*tert*-butyl-phenol and 4-phenyl-phenol, also afforded the desired products **3i** and **3j** in excellent stereocontrol with a modified reaction conditions, respectively, demonstrating that the substrate scope could not be only limited to naphthols.

Next, we explored the generality of the reaction with regard to variation of ATADs. A broad range of ATADs containing different substituents at the aromatic ring reacted smoothly with 2-naphthol **1a** to produce the corresponding axially chiral urazoles with high efficiency and excellent entantiocontrol (Table 3). The electronic and position properties of the aromatic ring substituents did not affect the selectivities of the tyrosine click reactions. It should be pointed out that the ortho group is not only restricted to *tert*-butyl group or iodo, and the bromo or phenyl group at the ortho position could also be obtained with excellent enantioselectivities (**3p** and **3q**). It should be emphasized that the presence of I or Br is very convinient to do the further transformation for diversity-oriented synthesis and drug discovery due to the high reactivity in many transition metal-catalysed reactions^44^. Experiments on the configurational stability of the product were carried out by heating a solution of **3a** in toluene or MeCN at 80 °C for 12 h. Chiral HPLC analysis showed that the ee value of **3a** did not have any effect. Therefore, the obtained axially chiral compounds may have potential wide applications as asymmetric organocatalysts/ligands.

### Optimization of reaction conditions involving indoles

To expand the synthetic utility of this methodology and further develop the application of the very reactive ATAD, we next focused our attention on more challenging nucleophiles. Although much progress has been made in the development of organocatalytic asymmetric intermolecular transformation by using indoles as nucleophiles^45^,^46^, to the best of our knowledge, only few examples involving 2-substituted indoles as nucleophile have been reported with good enantiocontrol, which is probably ascribed to the interrupted interaction between the substrates and the organocatalyst^47^. We envisaged that the very reactive and multifunctional electrophile ATAD might provide new possibility to proceed such a remote control process with good stereoselectivity with bifunctional organocatalysts. To our delight, by using the standard reaction conditions (Table 4, entry 1), we found that the reaction of 2-phenylindole **4a** with 4-(2-*tert*-butylphenyl)-3*H*-1,2,4-triazole-3,5-dione (**2a**) proceeded smoothly by simply using the catalyst **C6**, giving the desired product **5a** in 74% yield with 15% ee. However, after making great efforts on investigation of the optimized reaction conditions, we could not improve the enantioselectivity by using thiourea-tertiary amine organocatalyst (see Supplementary Table 1 for details). On the basis of these findings and own comprehension on the phosphoric acid catalysis^48^,^49^,^50^, we envisioned that phosphoric acid might perform bifunctional action to activate indole and ATAD simutaneously and control the enantioselectivity^51^,^52^,^53^,^54^. As shown in Table 4, phosphoric acid catalyst proved to be a suitable organocatalyst for this tranformation. On optimizing the reaction conditions through variation of the phosphoric acid catalysts, solvents and catalyst loadings (Table 4, entries 3–15), the following protocol was proved to be optimal: reaction of **4a** and **2a** with the molar ratio of 1.0:1.2 by using phosphoric acid **CP5** (5 mol%) as catalyst in DCM/Et_2_O (1/1) at −78 °C for 10 min, **5a** was obtained in exellent yield with 97% ee (Table 4, entry 15). It should be noted that the chiral spiro-phosphoric acid catalyst displayed better enantioselectivity than the BINOL-derived catalyst if the substituent in the 3 and 3′ positions is the same (entries 6 and 12; entries 10 and 13; entries 11 and 15).

### Substrate scope with indoles as nucleophiles

Having identified the optimized reaction conditions, the reaction was extended to include various 2-substituted indoles and triazoledione compounds with catalyst **CP5**. As shown in Table 5, the reaction proceeded smoothly to give the desired product **5a**–**5m** in very high yield (86–96%) and excellent enantioselectivity (84–97% ee). It should be noted that the electronic nature, bulkiness or positions of the substituents on the cyclic diazo compounds and substituted indoles have only minimal effect on efficiencies and enantioselectivities. In addition to aromatic groups, alkyl substituents on indole were used to acquire the desired products (**5l** and **5m**) with excellent yields and good stereoselectivities in just less than 5 min (Table 5, entries 12–13).

### Preliminary evaluation as chiral ligands

**Figure 2:**
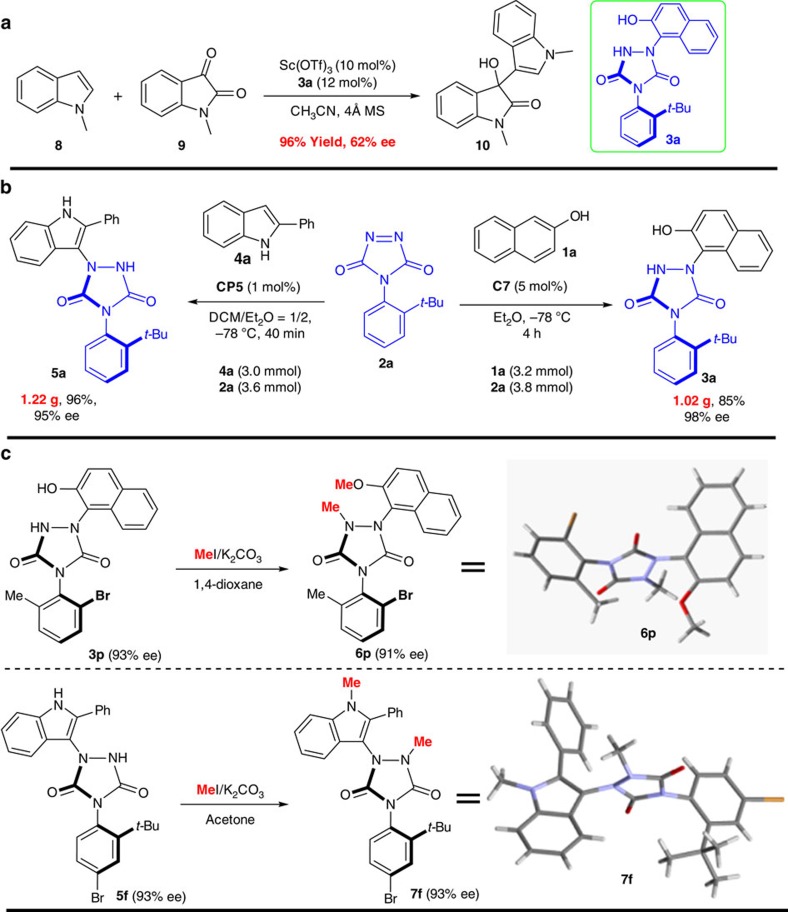
Application in asymmetric catalysis and gram-scale synthesis of 3a/5a and further transformation. (**a**) Potential application of catalytic asymmetric synthesis of substituted 3-hydroxy-2-oxindole. (**b**) Gram-scale synthesis of axially chiral urazoles via tyrosine click reaction. (**c**) Further transformation for confirmation of absolute configuration.

To verify the stability of such axial compounds, we heated the obtained product **5a** in MeCN at 80 °C for 12 h and no ee erosion was observed. Thus, this kind of axially chiral compounds displayed a high rotation energy about the N–Ar bond, indicating that the chiral urazoles may have potential applications in the field of asymmetric organocatalysts and Lewis acid catalysis. To really investigate the potential application of the resultant axially chiral urazoles in the field of asymmetric catalysis, we chose the addition of *N*-methylindole (**8**) to *N*-methylisatin (**9**) as a model reaction and evaluated the potential application in the asymmetric catalysis (see Supplementary Table 2). Gratifyingly, the reaction proceeded completely within 8 h at 5 °C and the desired product (**10**) was obtained in 96% yield with 62% ee (Fig. 2a), demonstrating that the newly developed axially chiral urazoles have the potential application in asymmetric synthesis. Further work encompassing the application of axially chiral urazoles as ligands or catalysts for enantioselective reactions is currently in progress in our laboratory.

### Gram-scale synthesis of enantiopure urazoles

To further demonstrate the utility of the tyrosine click-like reaction, gram-scale syntheses of products **3a** and **5a** were carried out. As displayed in Fig. 2b, there was almost no change in reactivity and stereoselectivity, suggesting that this method should have the potential for large-scale chemical production (also see Supplementary Note 3). It should be worth highlighting that the reaction by using 2-phenyl indole as nucleophile was proceeded very smoothly, with only 1 mol% of phosphoric acid catalyst **CP5**. The absolute configuration of **3p** was attributed to be *aS* and **5f** was assigned to be *aR* using X-ray diffraction analysis of their methylation derivatives **6p** and **7f** (Fig. 2c, see also Supplementary Fig. 1).

## Discussion

We have successfully developed an organocatalytic asymmetric tyrosine click-like reaction in high yields with excellent enantioselectivity under mild reaction conditions in an excellent remote enantiocontrol manner. The reaction represents a very convenient approach to an interesting class of axially chiral urazole derivatives, with potential biological activities and potential application as effective chiral organocatalysts/ligands. The excellent remote enantiocontrol of the process stems from the efficient discrimination of the two reactive sites in the triazoledione-involving phenols or indoles as nucleophile and transferring the chirality of the catalyst into the axial chirality of urazoles at the remote position far from the reactive site. The application of this strategy to a broader substrate scope and mechanistic investigations of the desymmetrization strategy are currently underway in our group.

## Methods

### General information

Reagents were purchased at the highest commercial quality and used without further purification, unless otherwise stated. Analytical thin layer chromatography (TLC) was performed on precoated silica gel 60 F254 plates. Flash column chromatography was performed using Tsingdao silica gel (60, particle size 0.040–0.063 mm). Visualization on TLC was achieved by the use of ultraviolet light (254 nm). NMR spectra were recorded on a Bruker DPX 400 spectrometer at 400 MHz for ^1^H NMR, 100 MHz for ^13^C NMR and 376 MHz for ^19^F NMR in CDCl_3_ or acetone-*d*_6_ with tetramethylsilane as internal standard. Chemical shifts are reported in p.p.m., and coupling constants are given in Hz. Data for ^1^H NMR are recorded as follows: chemical shift (p.p.m.), multiplicity (s, singlet; d, doublet; t, triplet; q, quartet; m, multiplet), coupling constant (Hz) and integration. Data for ^13^C NMR are reported in terms of chemical shift (*δ*, p.p.m.). High-resolution mass spectra were recorded on a LC-TOF spectrometer (Micromass). Enantiomeric excess was determined on Agilent HPLC using the DAICEL CHIRAL column. For preparation of 4-aryl-l,2,4-triazoline-3,5-diones, see Supplementary Note 1.

Racemic compounds were obtained without catalyst.

### General procedure for synthesis of axially chiral urazoles **3**

In a Schlenk tube, 4-aryl-l,2,4-triazoline-3,5-diones **2** (0.12 mmol) and catalyst **C7** (5 mol%, 0.005 mmol) were dissolved in Et_2_O (2 ml; also see Supplementary Note 2). The solution was stirred for 10 min at −78 °C before 2-naphthols and phenols 1 (0.10 mmol) were added. The resulting solution was stirred at −78 °C until the red colour disappeared. After monitored with TLC, the reaction mixture was acidified with 6 N HCl and concentrated. Then, the obtained crude material was purified using silica gel column chromatography (CH_2_Cl_2_ to CH_2_Cl_2_/Acetone=10/1) to afford the pure products **3**. In some cases, reactions were performed with 20 mol% of catalyst **C7** in 2.0 ml solvent, for **3d** in DCM at −78 °C; **3i** and **3j** in dry toluene at −40 °C.

### General procedure for synthesis of axially chiral urazoles **5**

In a Schlenk tube, 4-aryl-l,2,4-triazoline-3,5-diones **2** (0.12 mmol) and catalyst **CP5** (5 mol%, 0.005 mmol) were dissolved in DCM/Et_2_O=1/1 (2 ml; also see Supplementary Note 2). The solution was stirred for 10 min at −78 °C before 2-substituted indole **4** (0.10 mmol) was added. The resulting solution was stirred under this condition until the purple colour disappeared. After being monitored with TLC, the reaction mixture was concentrated, and then purified using silica gel column chromatography (CH_2_Cl_2_/Acetone=20/1) to afford the pure products **5**.

## Additional information

**Accession codes:** The X-ray crystallographic coordinates for structures reported in this Article have been deposited at the Cambridge Crystallographic Data Centre (CCDC), under deposition numbers CCDC 1439772 and CCDC 1440020. These data can be obtained free of charge from The Cambridge Crystallographic Data Centre via http://www.ccdc.cam.ac.uk/data_request/cif.

**How to cite this article:** Zhang, J.-W. *et al*. Discovery and enantiocontrol of axially chiral urazoles via organocatalytic tyrosine click reaction. *Nat. Commun.* 7:10677 doi: 10.1038/ncomms10677 (2016).

## Supplementary Material

Supplementary Information Supplementary Figures 1-101, Supplementary Tables 1-2, Supplementary Notes 1-3 and Supplementary References

## Figures and Tables

**Table 1:**
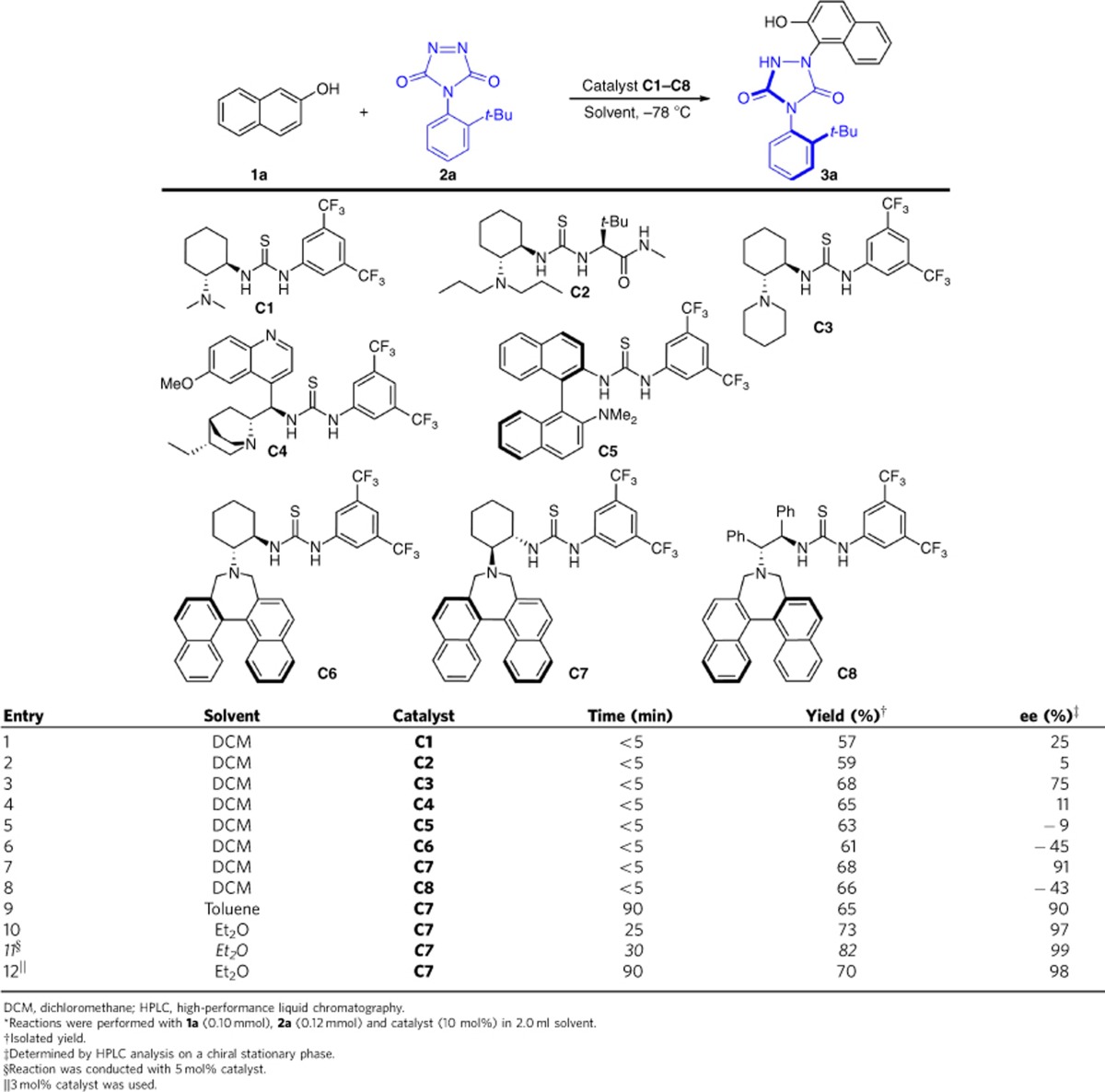
Optimization of the organocatalytic enantioselective tyrosine click reaction^*^.

**Table 2:**
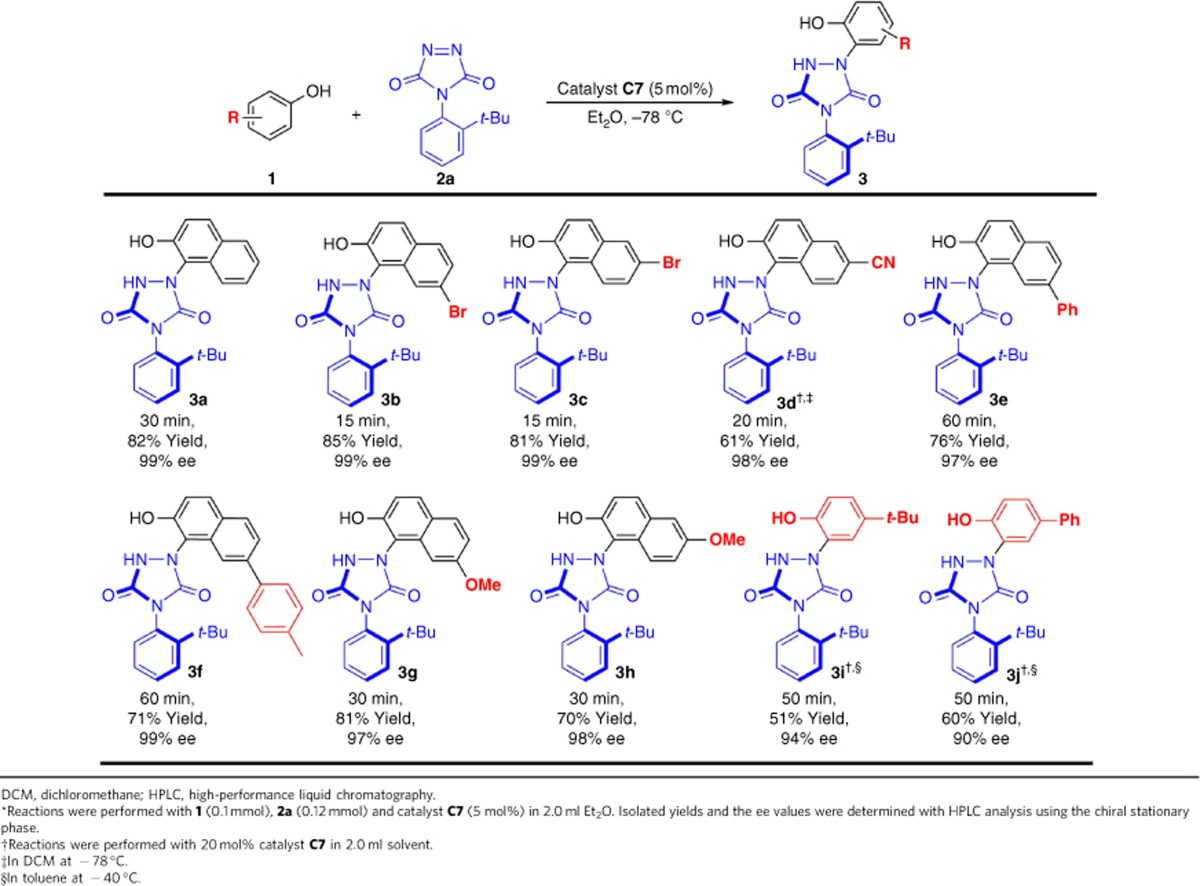
Substrate scope of naphthols or phenols^*^.

**Table 3:**
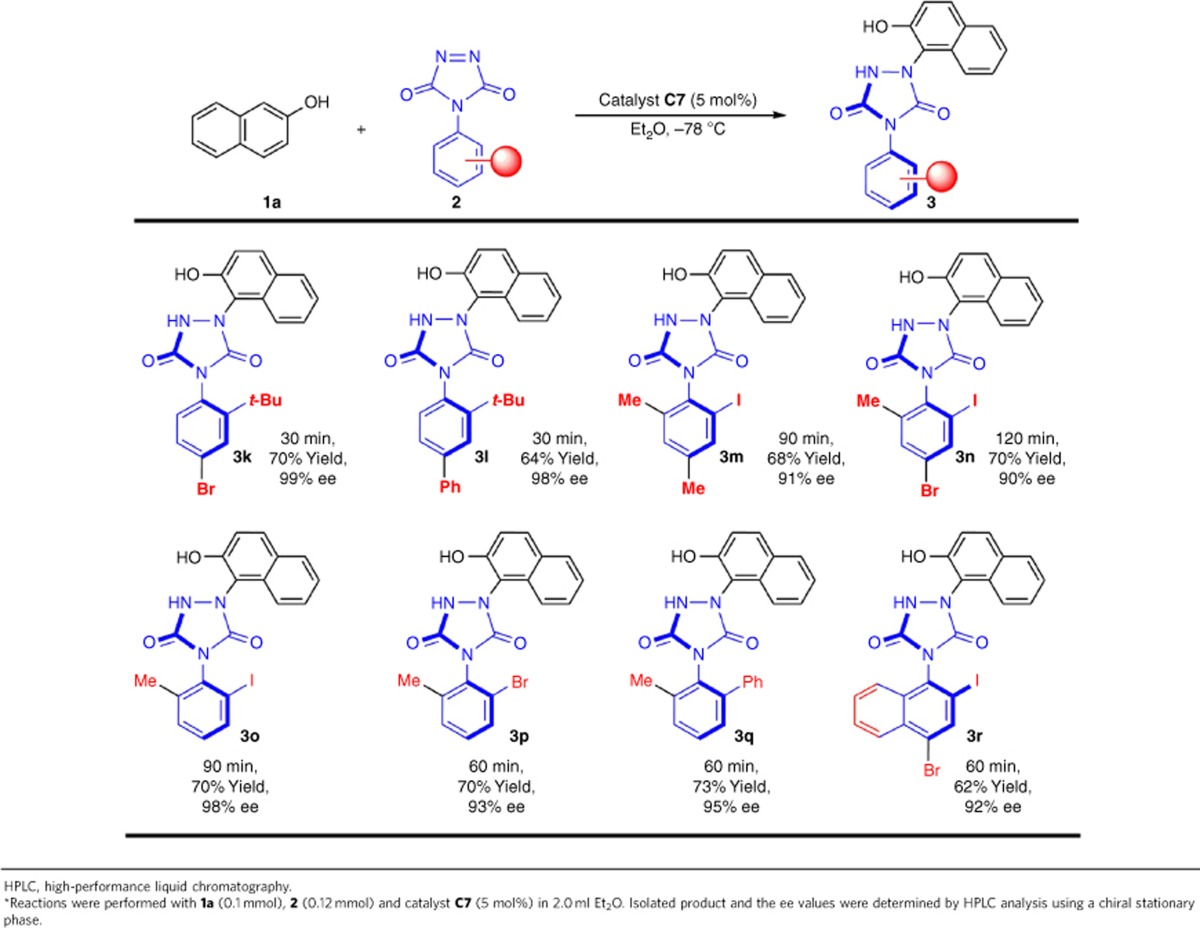
The reaction substrate scope of 4-aryl-1,2,4-triazoline-3,5-diones^*^.

**Table 4:**
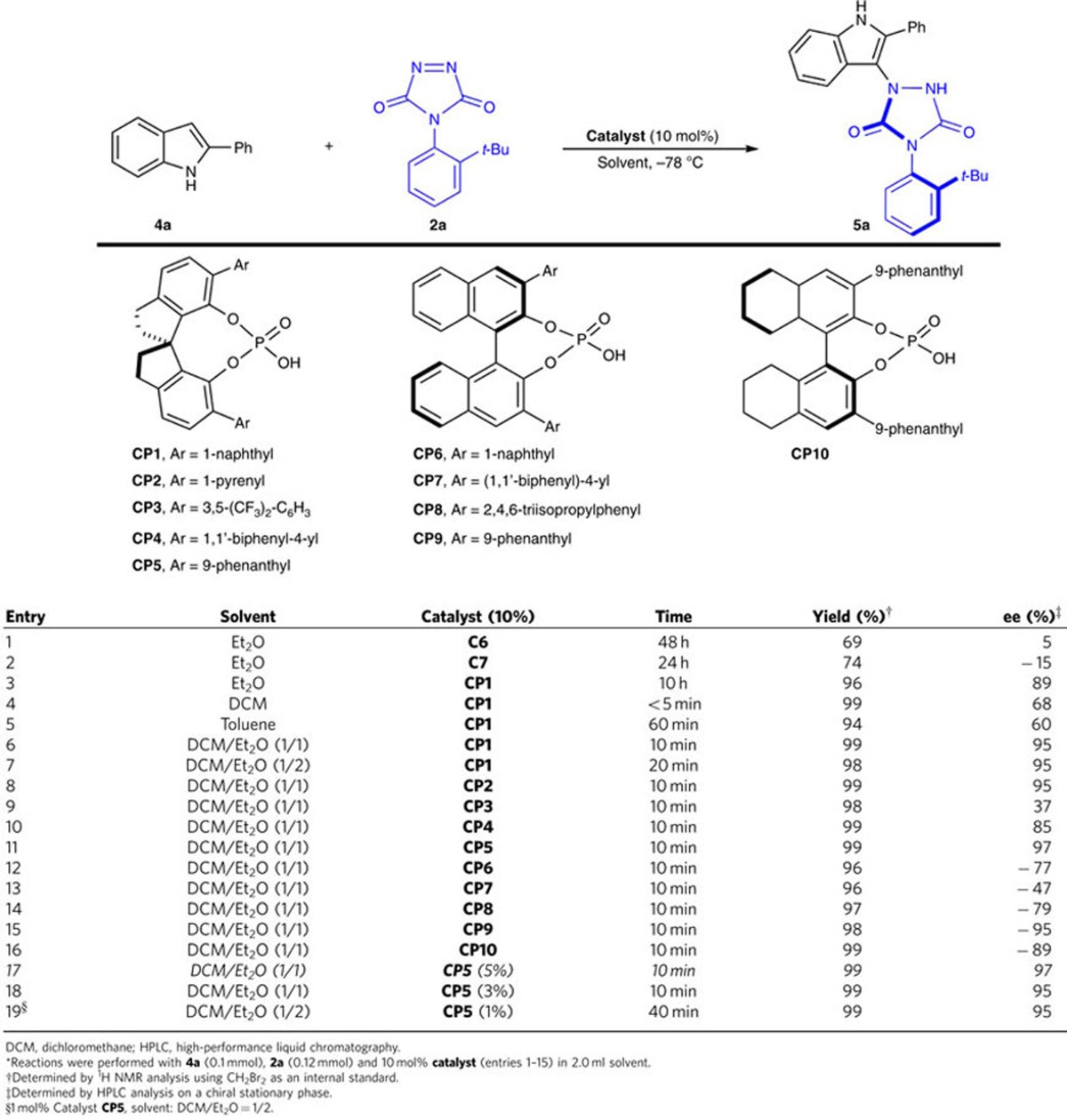
Optimization of the asymmetric tyrosine click-like reaction involving indoles as nucleophiles^*^.

**Table 5:**
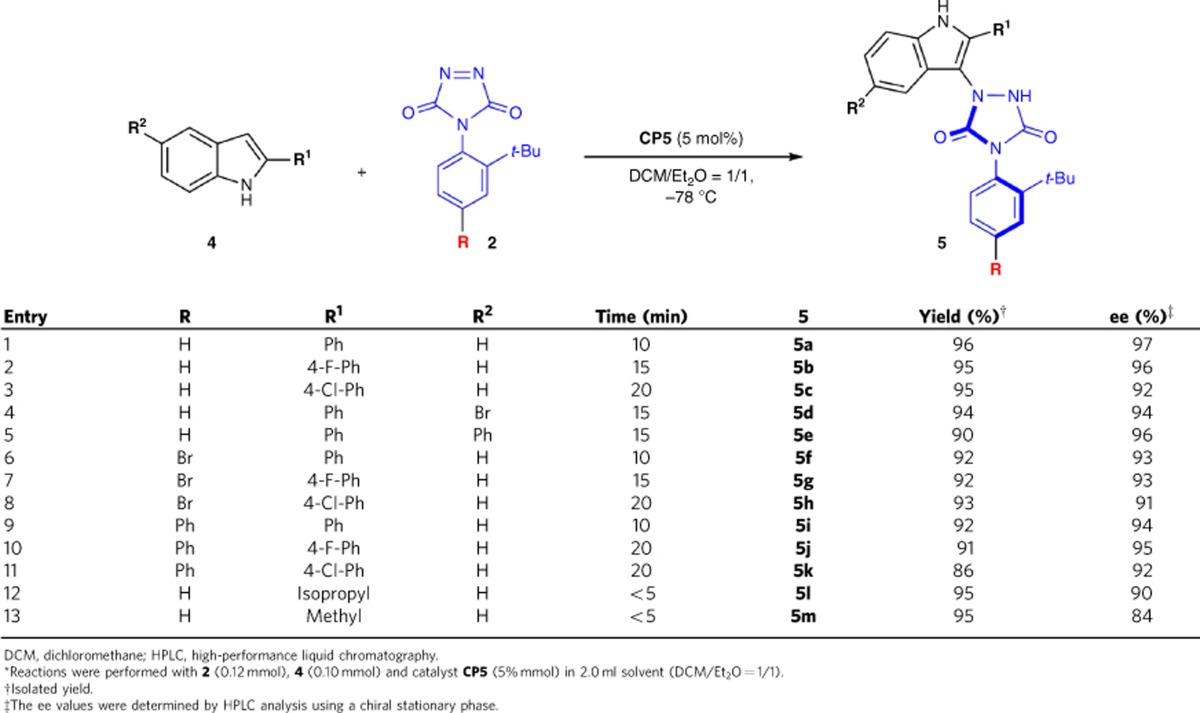
The substrate scope by using indoles as nucleophiles^*^.
