# Supplementary material for: Discovery and enantiocontrol of axially chiral urazoles via organocatalytic tyrosine click reaction
Source: Nat Commun. 2016 Feb 11;7:10677. doi: 10.1038/ncomms10677 (PMC4753251; doi:10.1038/ncomms10677)
Supplement: Supplementary Information — Supplementary Figures 1-101, Supplementary Tables 1-2, Supplementary Notes 1-3 and Supplementary References [file ncomms10677-s1.pdf]

Supplementary Figure 1

(X-ray structures of 6p and 7f)

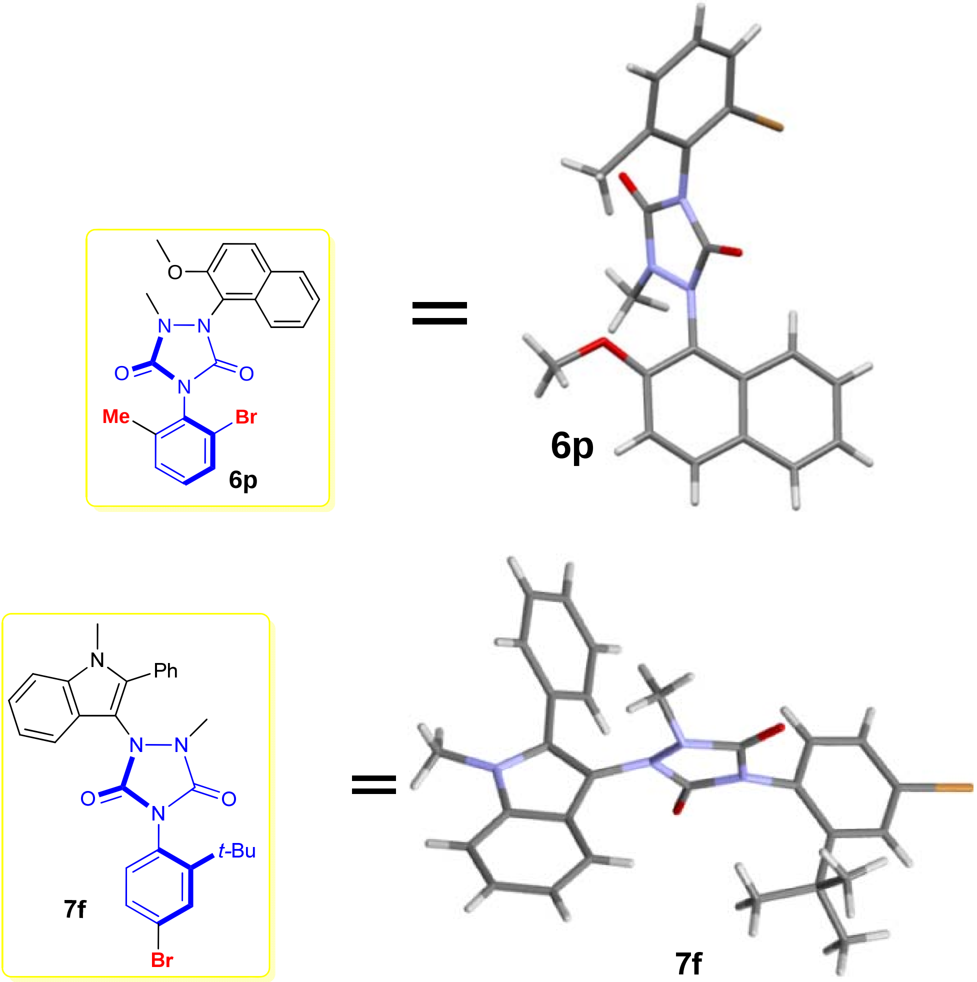

Supplementary Figures 2-68 (NMR Spectra)

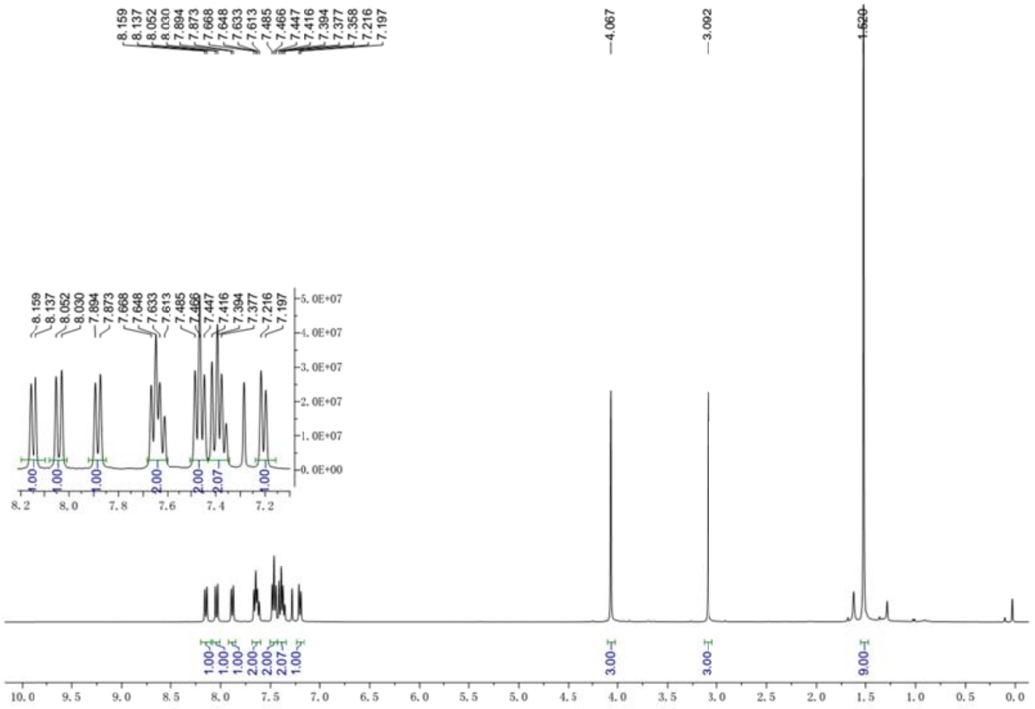

Supplementary Figure 2. ^{1}H NMR of the **6a**

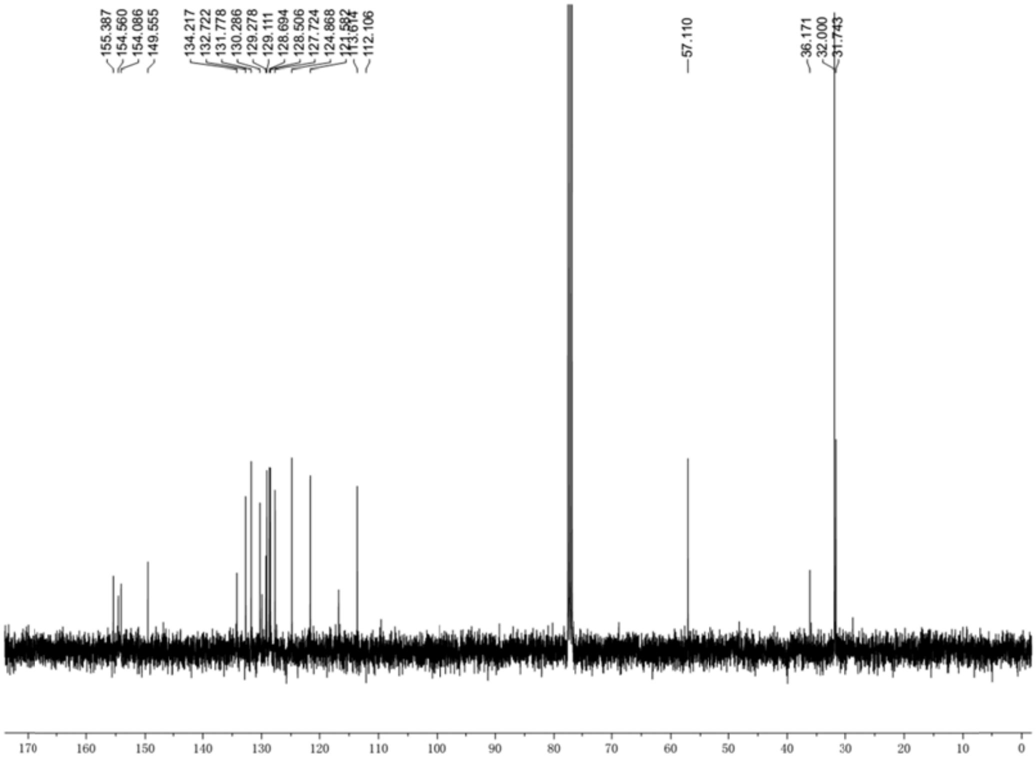

Supplementary Figure 3. ^{13}C NMR of the **6a**

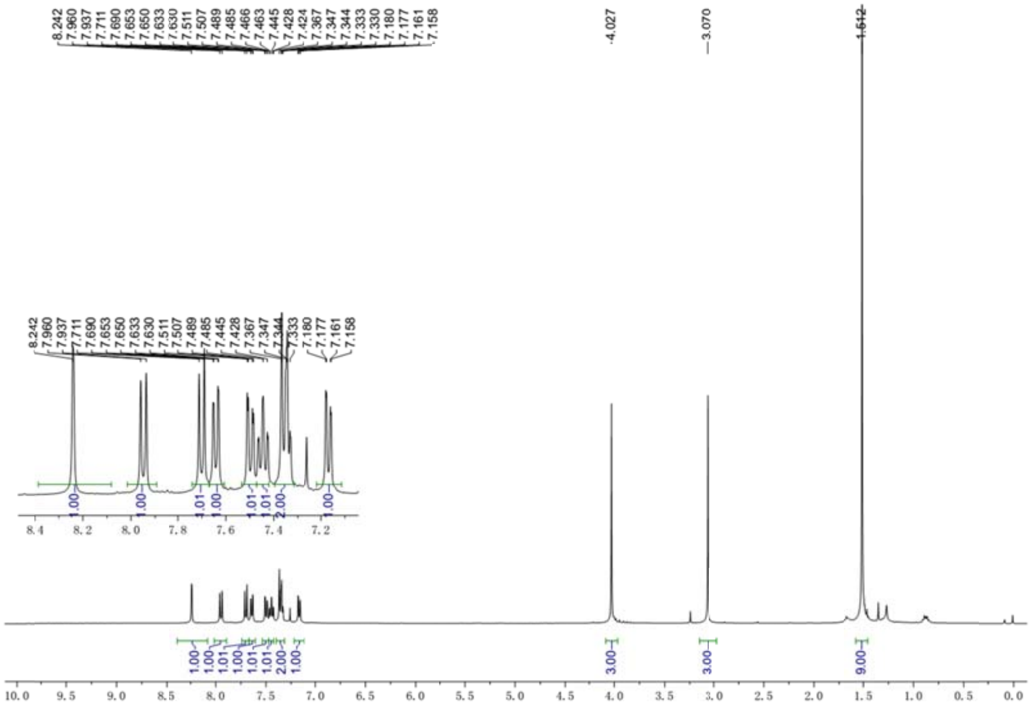

Supplementary Figure 4. ^{1}H NMR of the **6b**

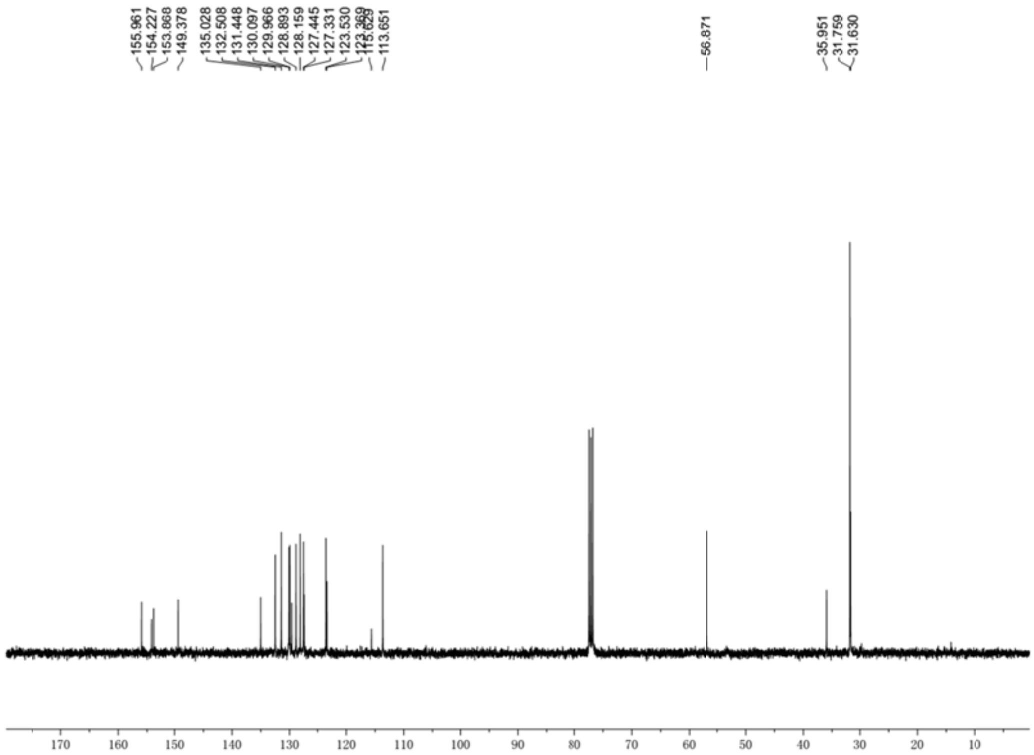

Supplementary Figure 5. ^{13}C NMR of the **6b**

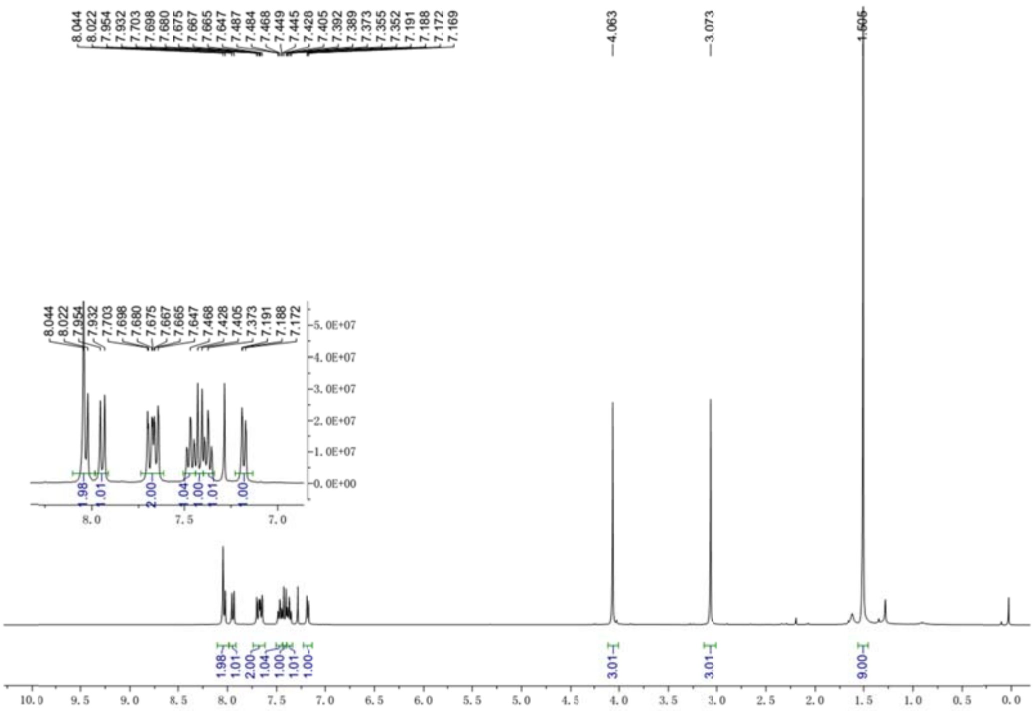

Supplementary Figure 6. ^{1}H NMR of the **6c**

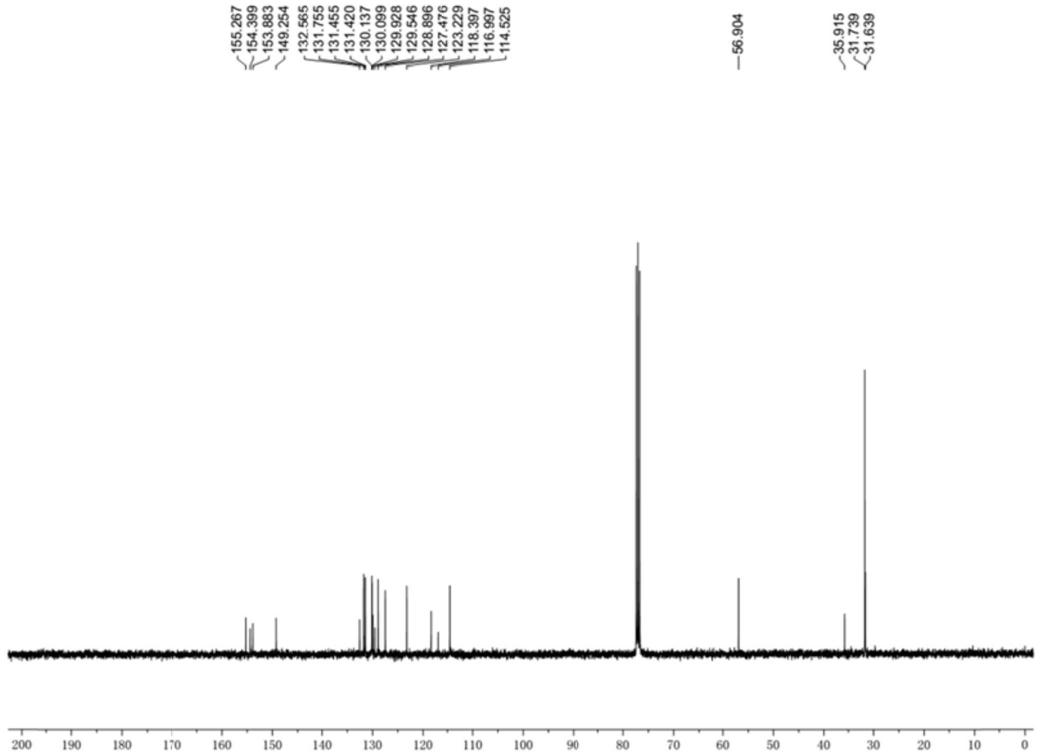

Supplementary Figure 7. ^{13}C NMR of the **6c**

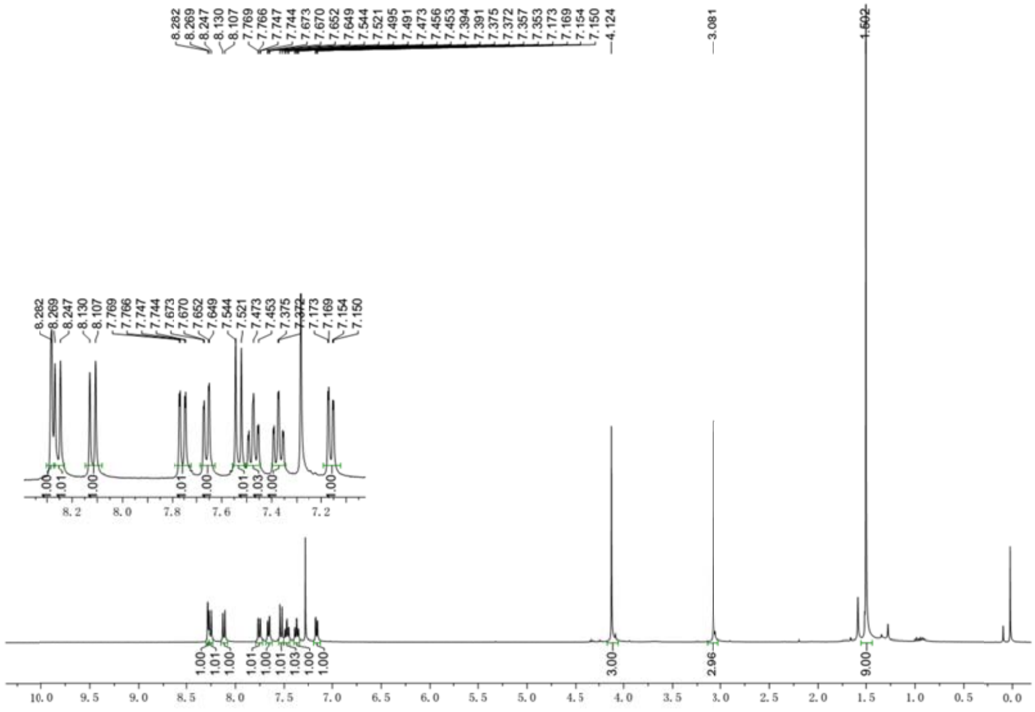

Supplementary Figure 8. ^{1}H NMR of the **6d**

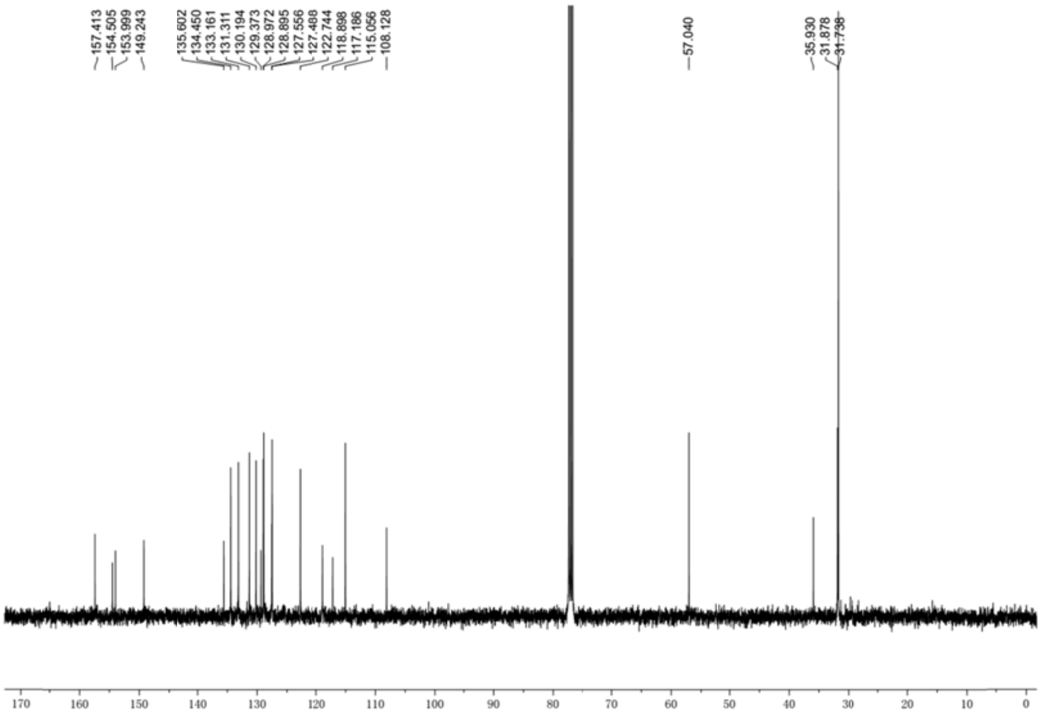

Supplementary Figure 9. ^{13}C NMR of the **6d**

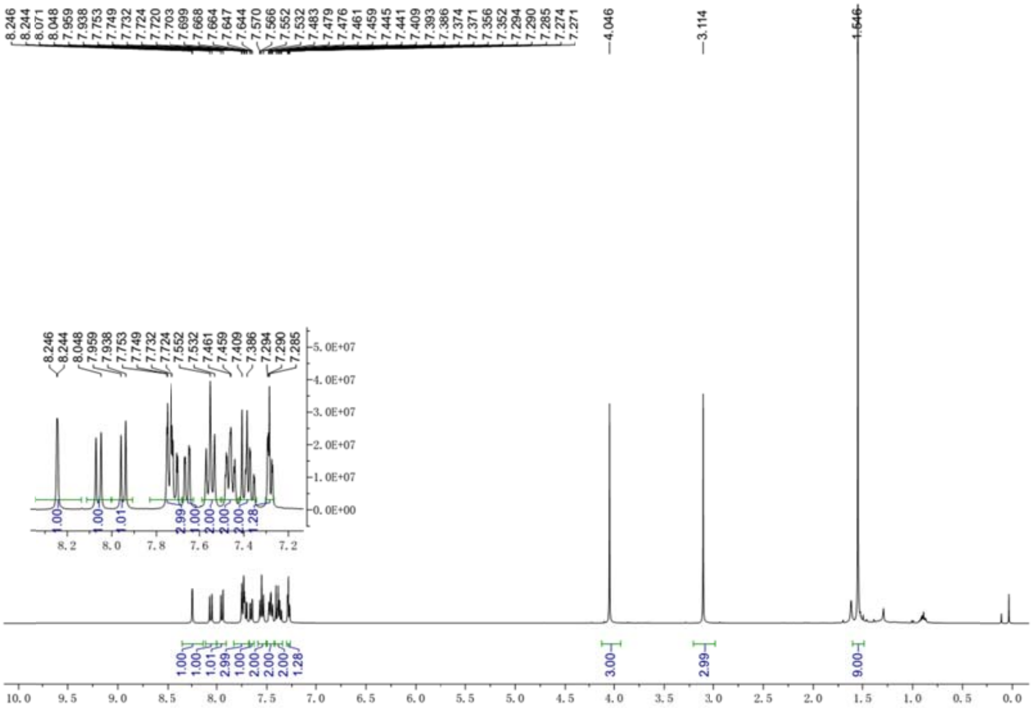

Supplementary Figure 10. ^{1}H NMR of the **6e**

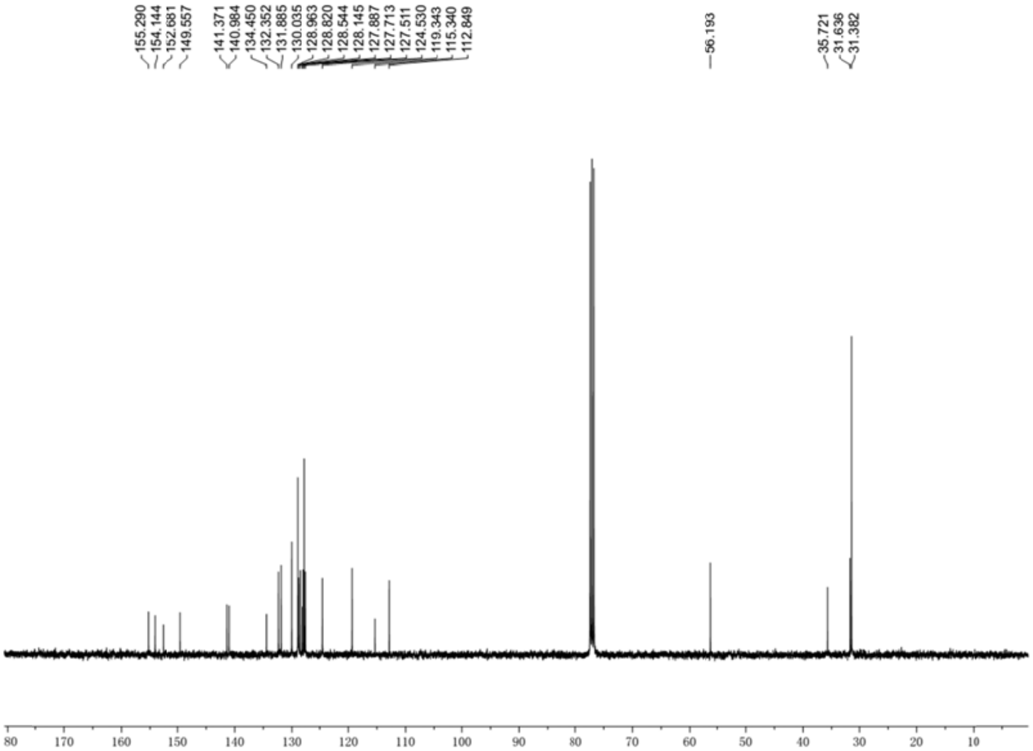

Supplementary Figure 11. ^{13}C NMR of the **6e**

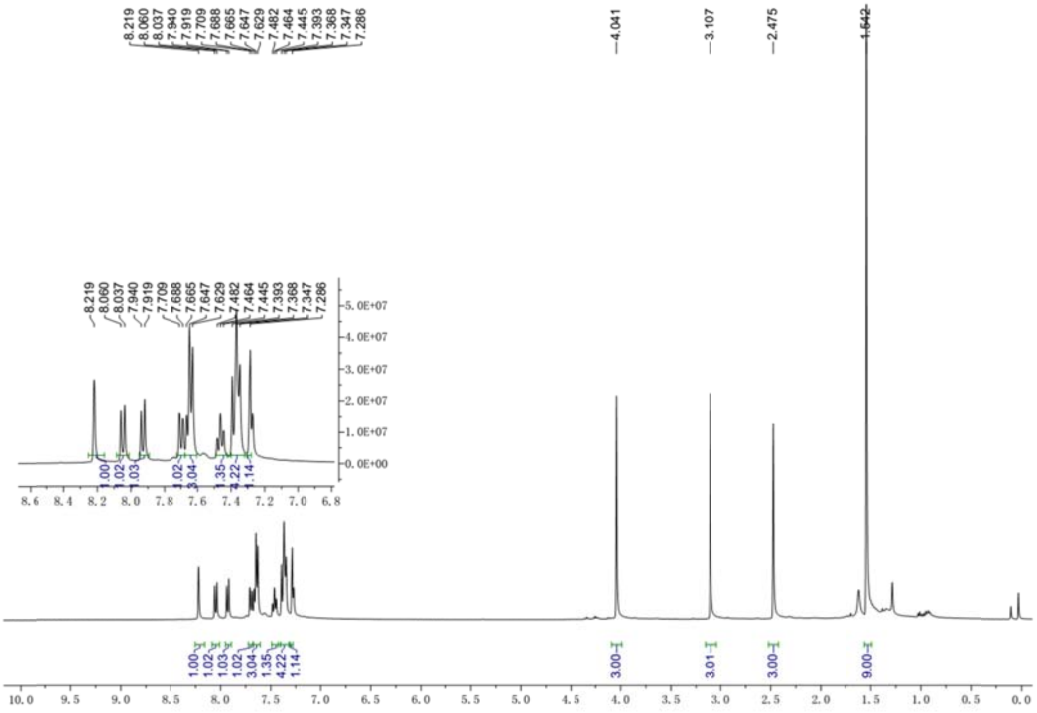

Supplementary Figure 12. ^{1}H NMR of the **6f**

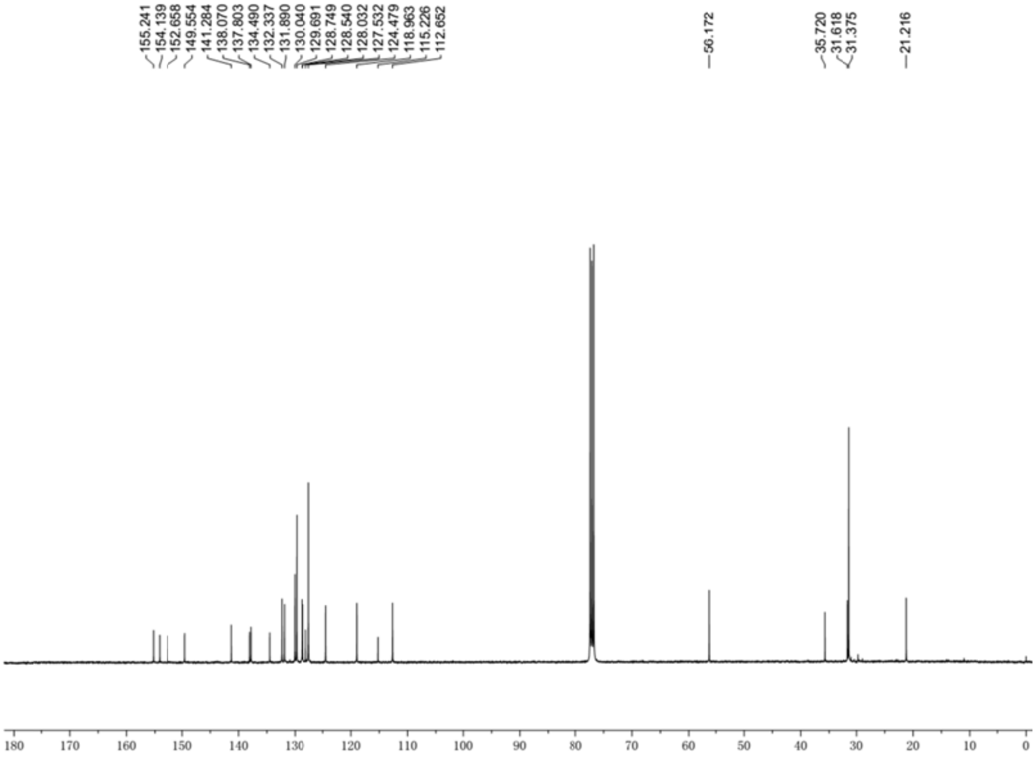

Supplementary Figure 13. ^{13}C NMR of the **6f**

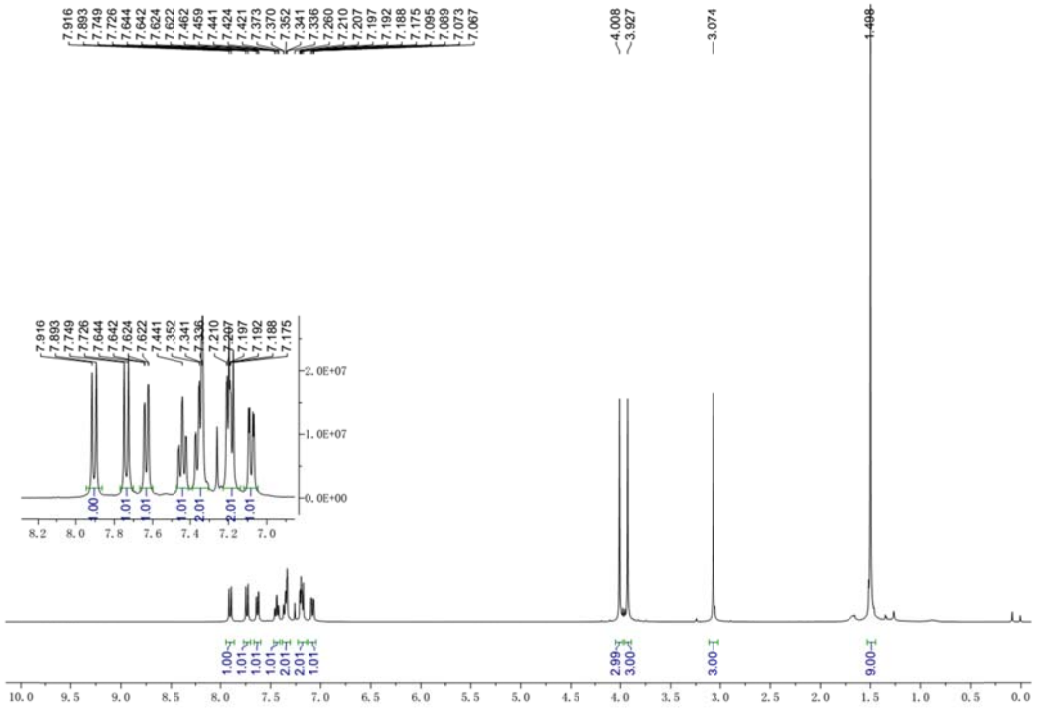

Supplementary Figure 14. ^{1}H NMR of the **6g**

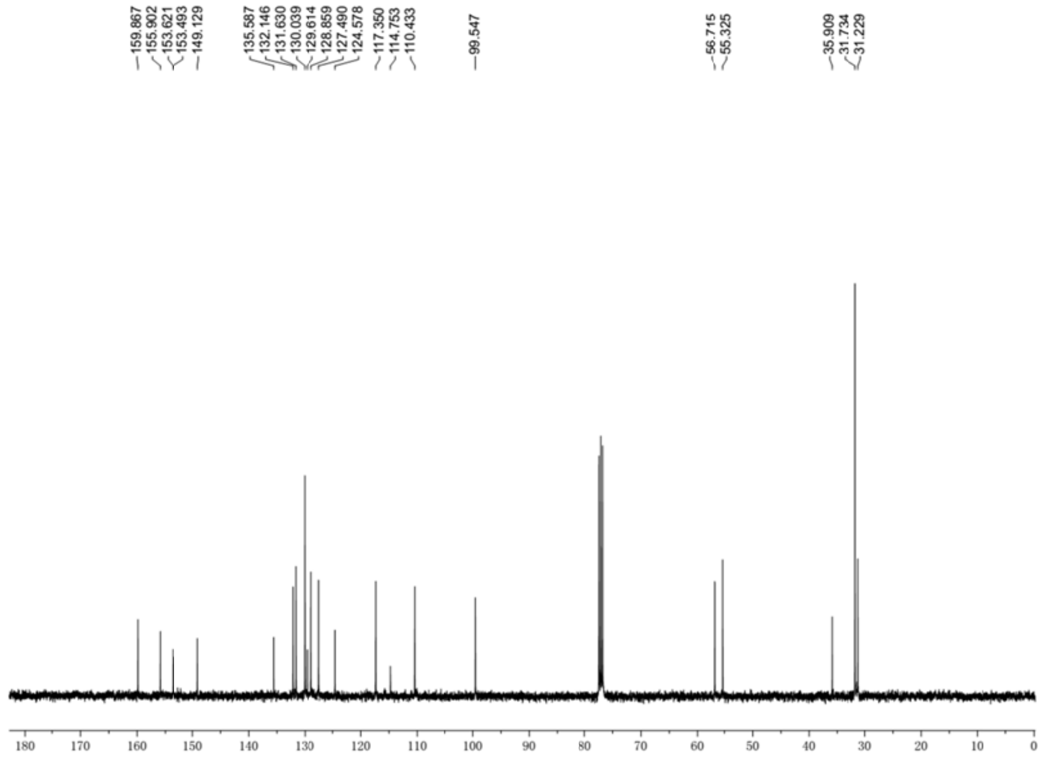

Supplementary Figure 15. ^{13}C NMR of the **6g**

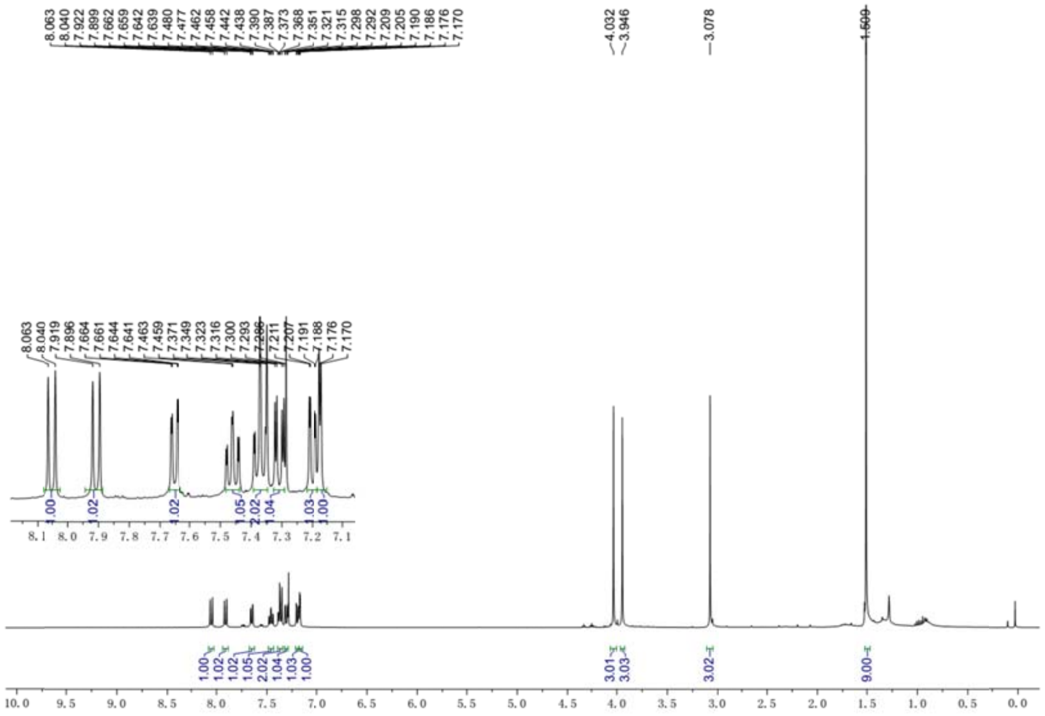

Supplementary Figure 16. ^{1}H NMR of the **6h**

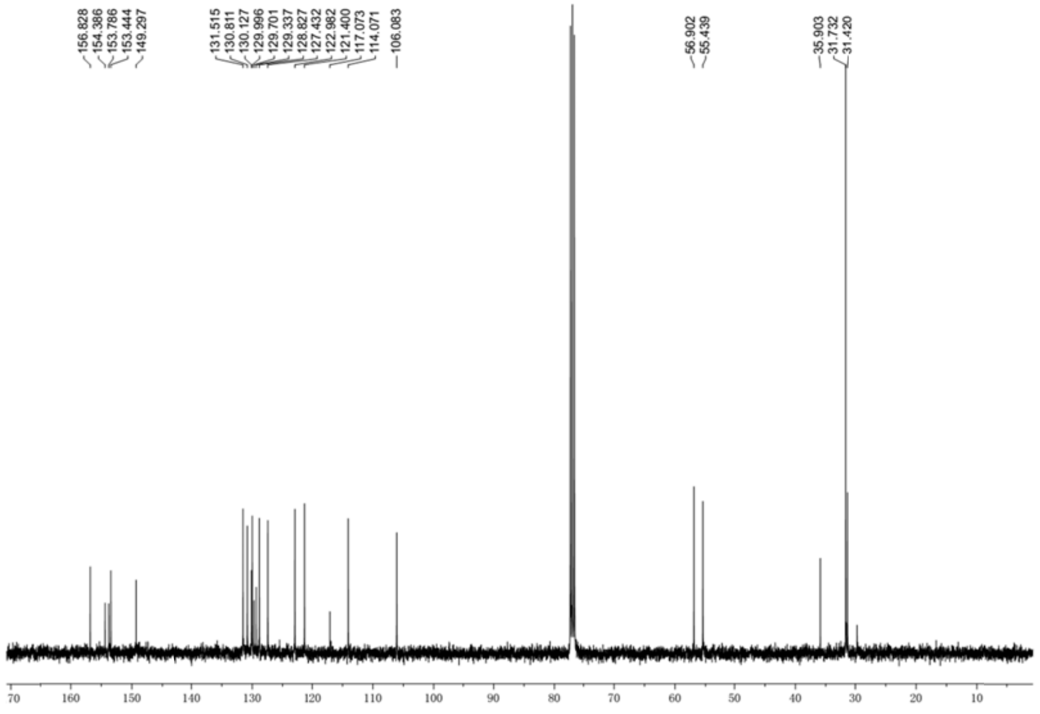

Supplementary Figure 17. ^{13}C NMR of the **6h**

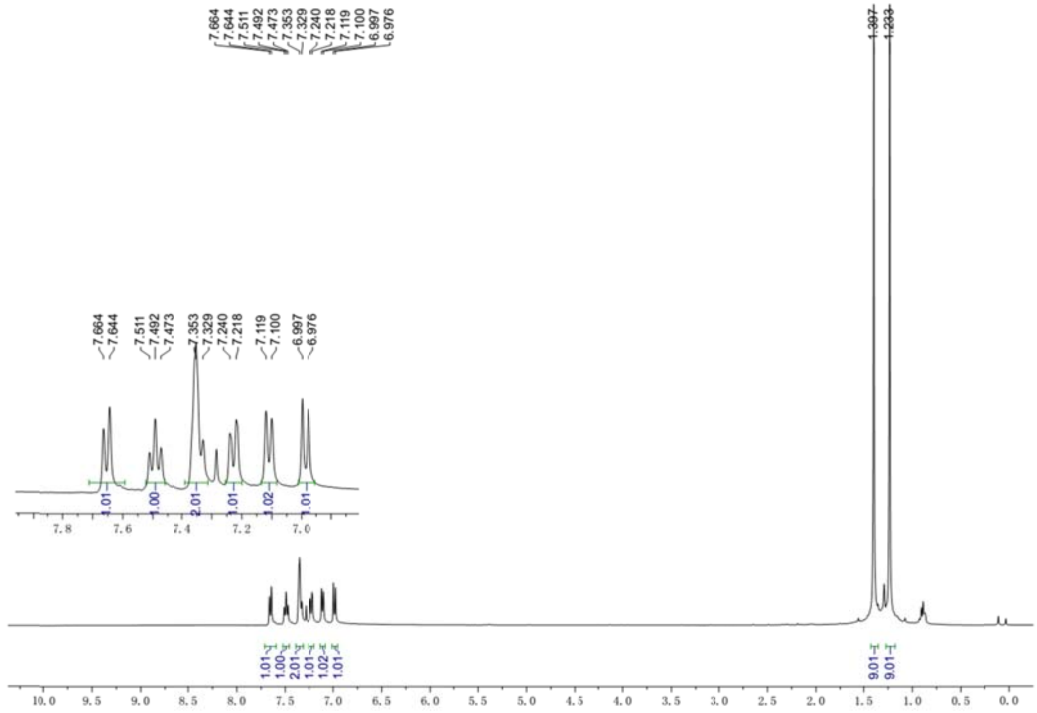

Supplementary Figure 18. ^{1}H NMR of the **3i**

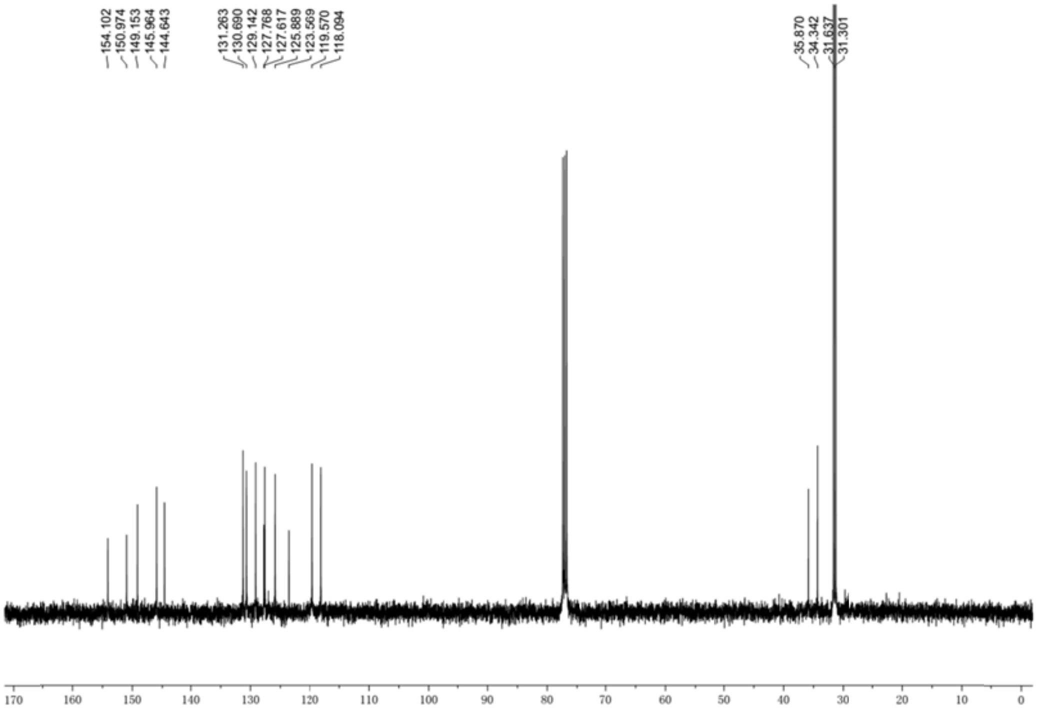

Supplementary Figure 19. ^{13}C NMR of the **3i**

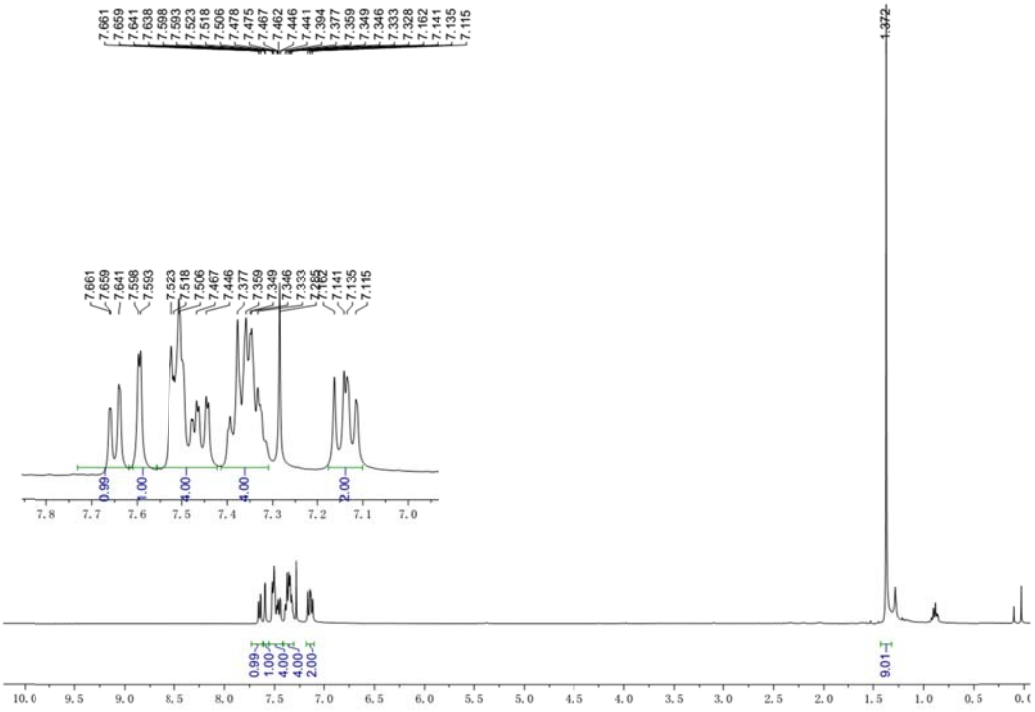

Supplementary Figure 20. ^{1}H NMR of the **3j**

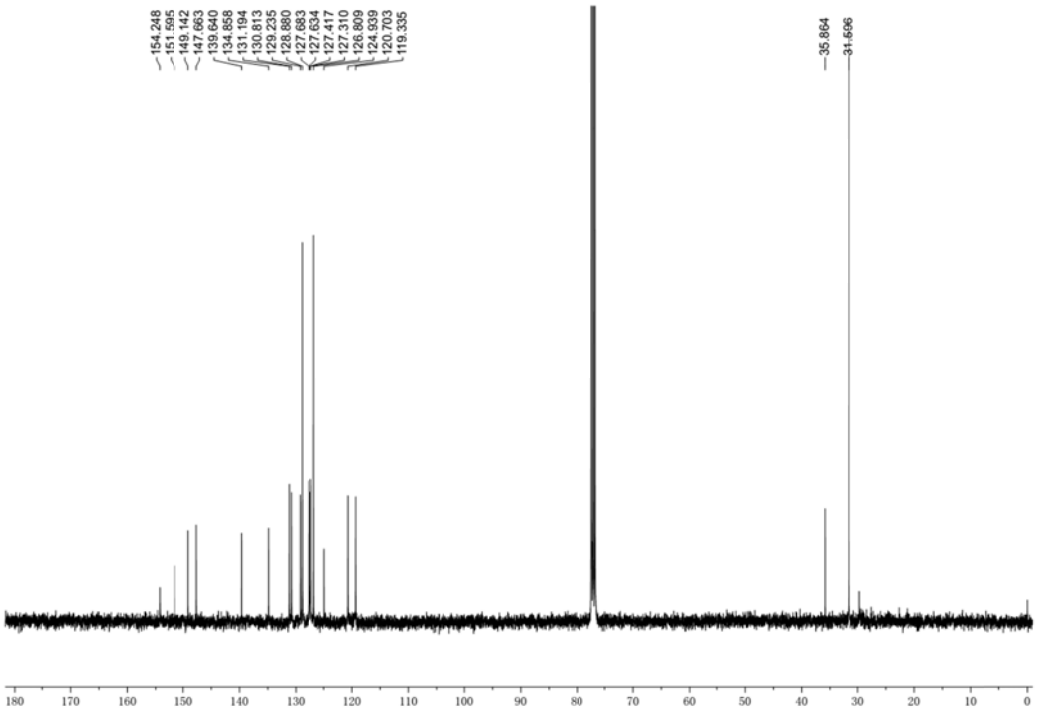

Supplementary Figure 21. ^{13}C NMR of the **3j**

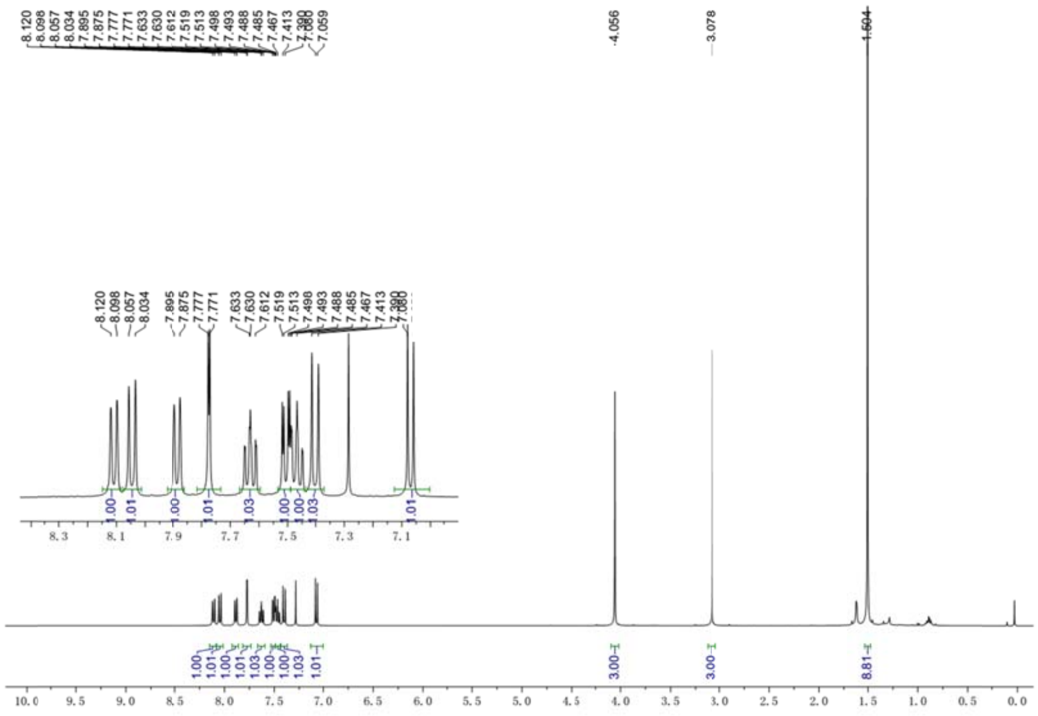

Supplementary Figure 22. ^{1}H NMR of the **6k**

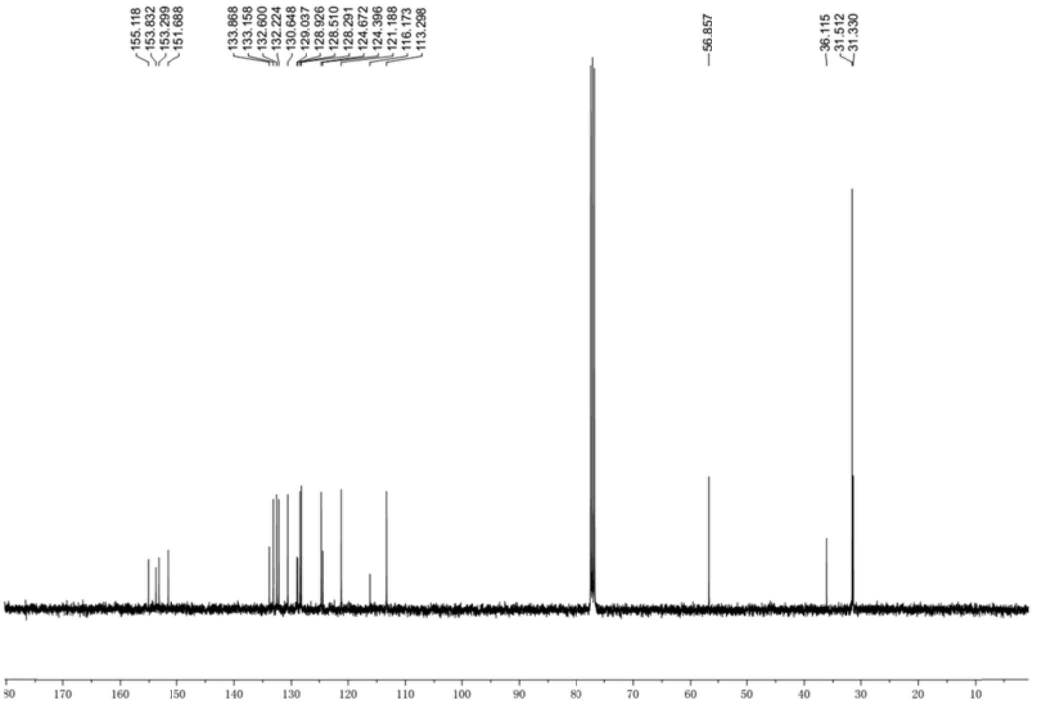

Supplementary Figure 23. ^{13}C NMR of the **6k**

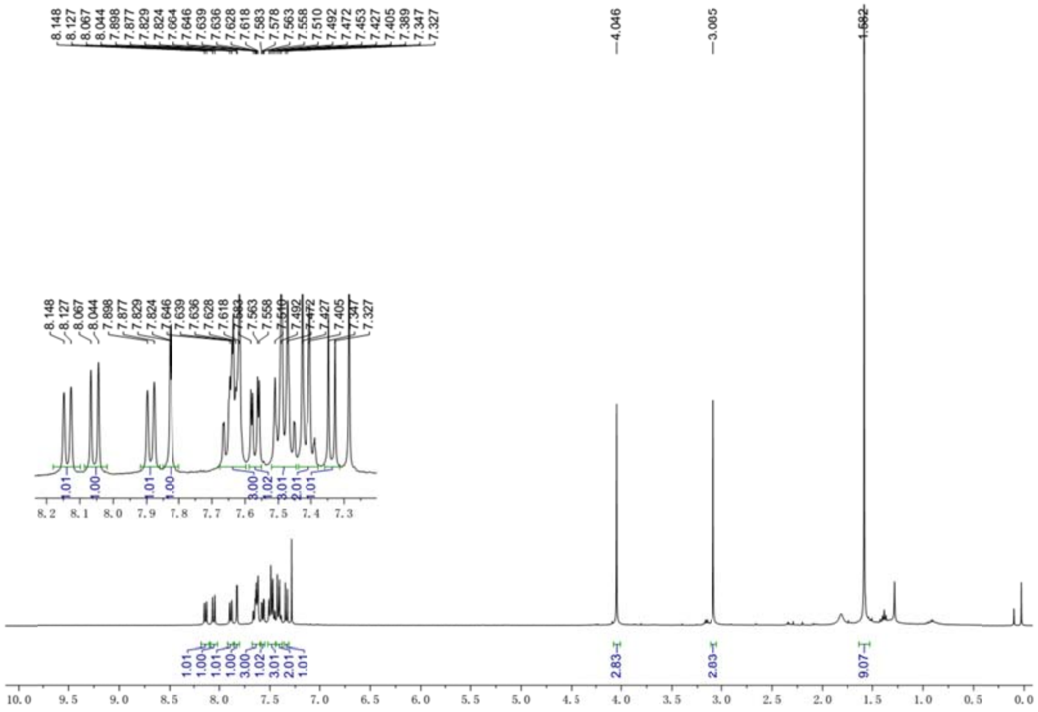

Supplementary Figure 24. ^{1}H NMR of the **6l**

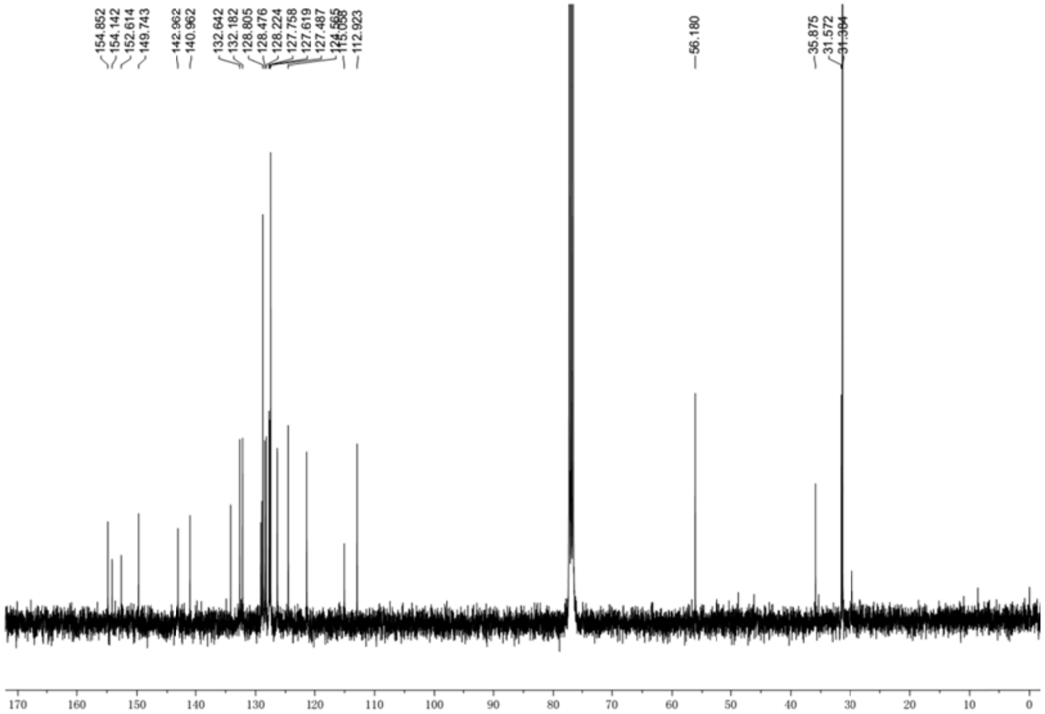

Supplementary Figure 25. ^{13}C NMR of the **6l**

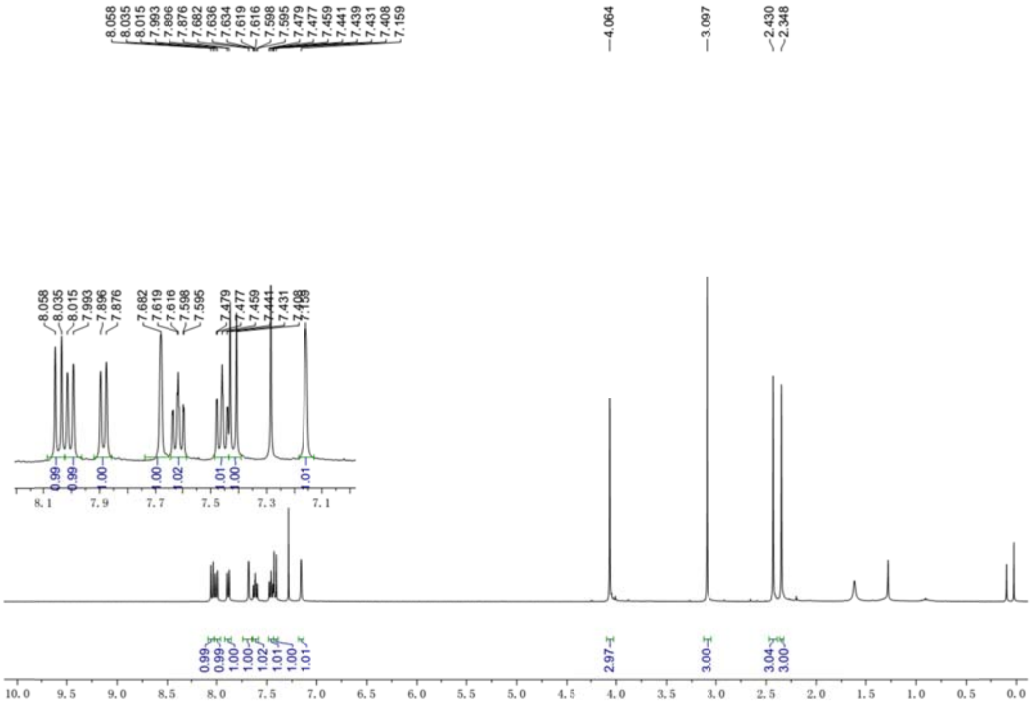

Supplementary Figure 26. ^{1}H NMR of the **6m**

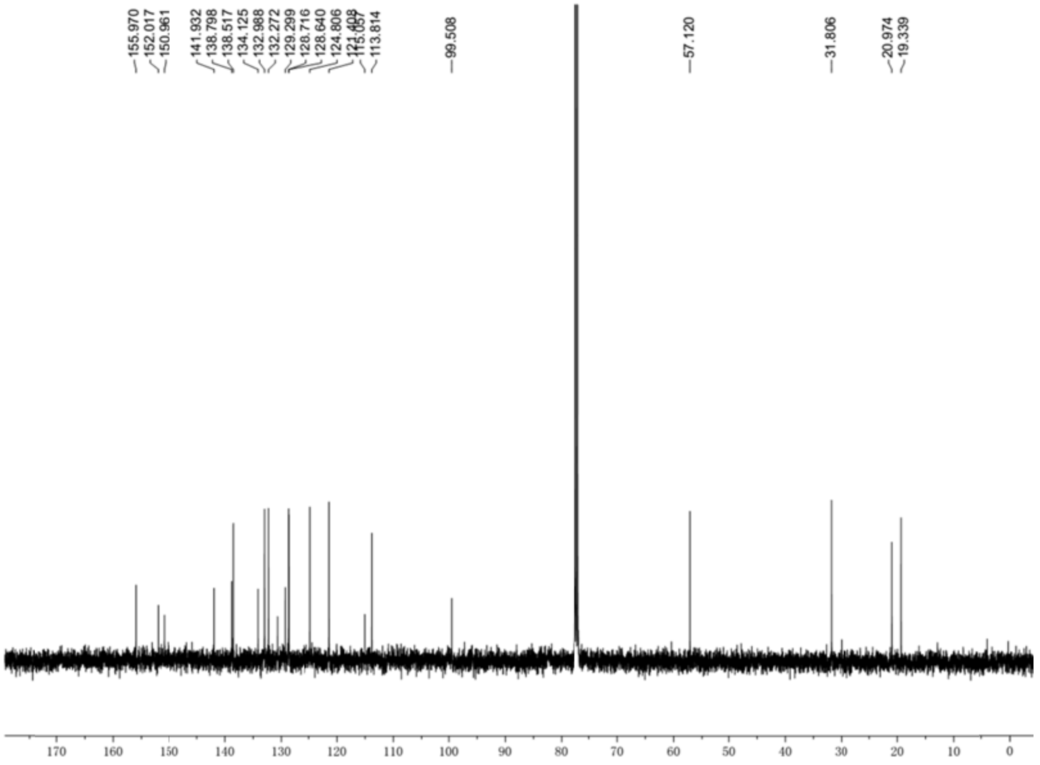

Supplementary Figure 27. ^{13}C NMR of the **6m**

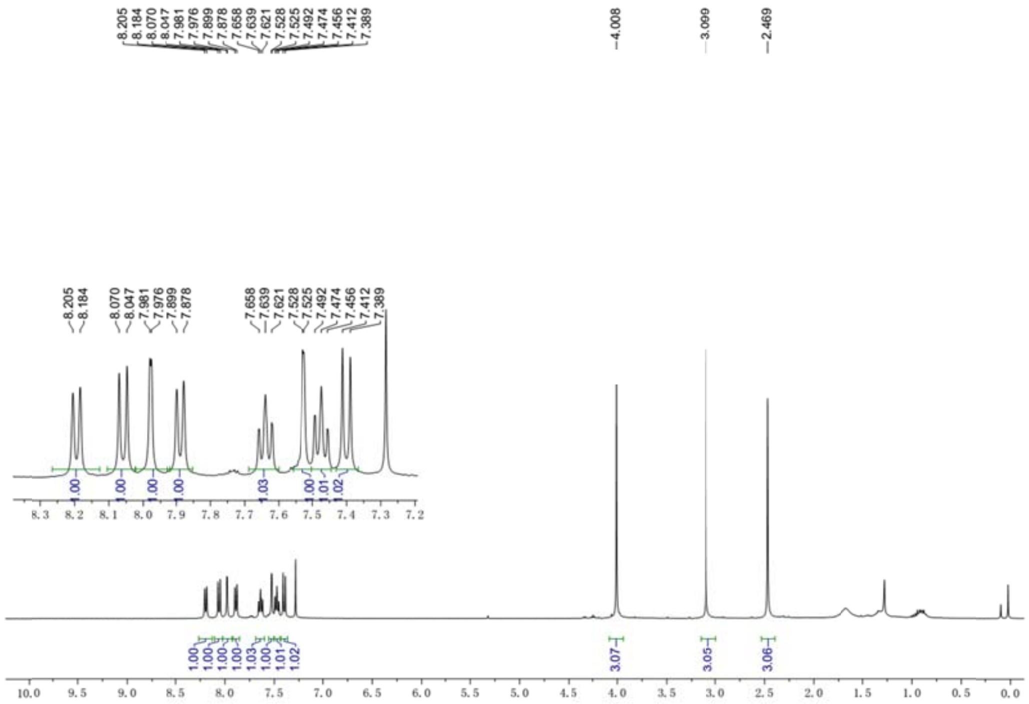

Supplementary Figure 28. ^{1}H NMR of the **6n**

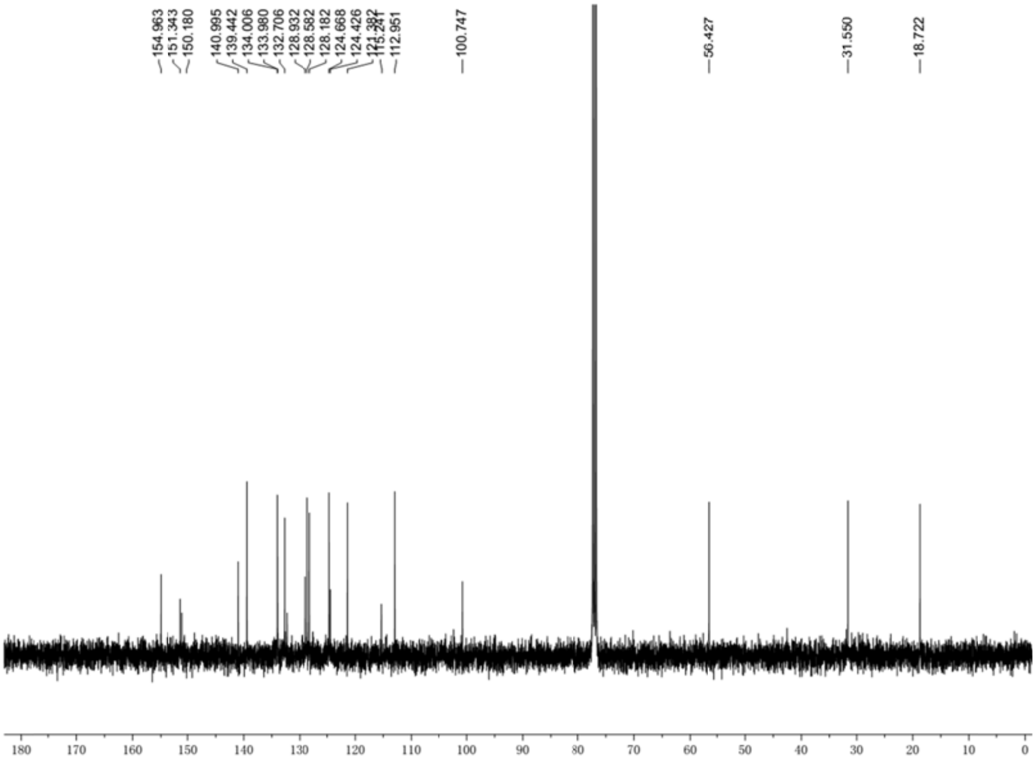

Supplementary Figure 29. ^{13}C NMR of the **6n**

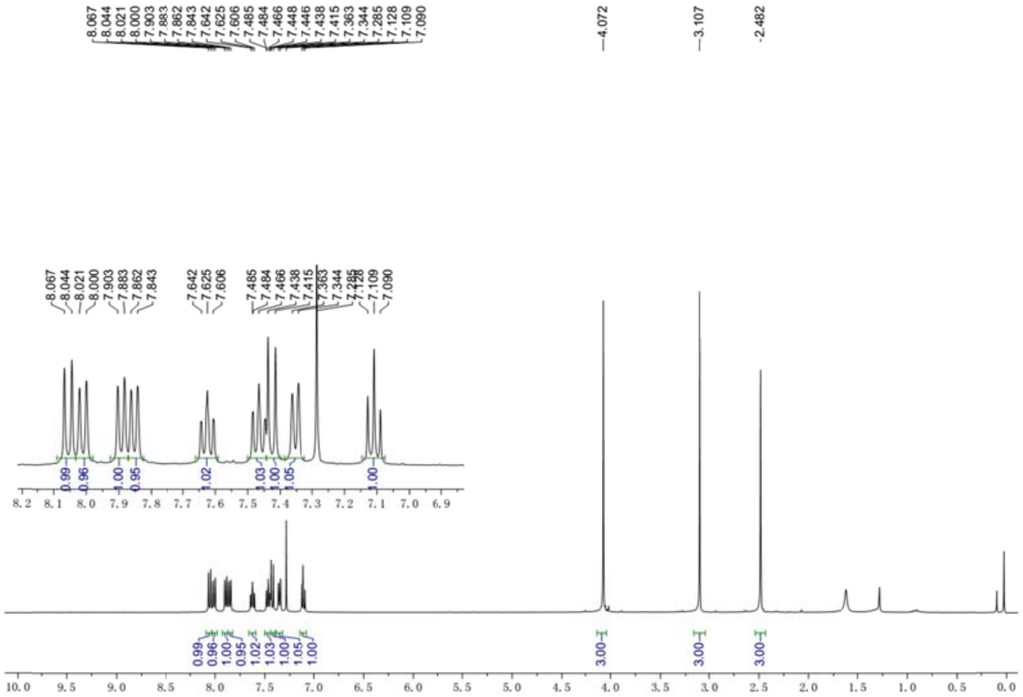

Supplementary Figure 30. ^{1}H NMR of the **6o**

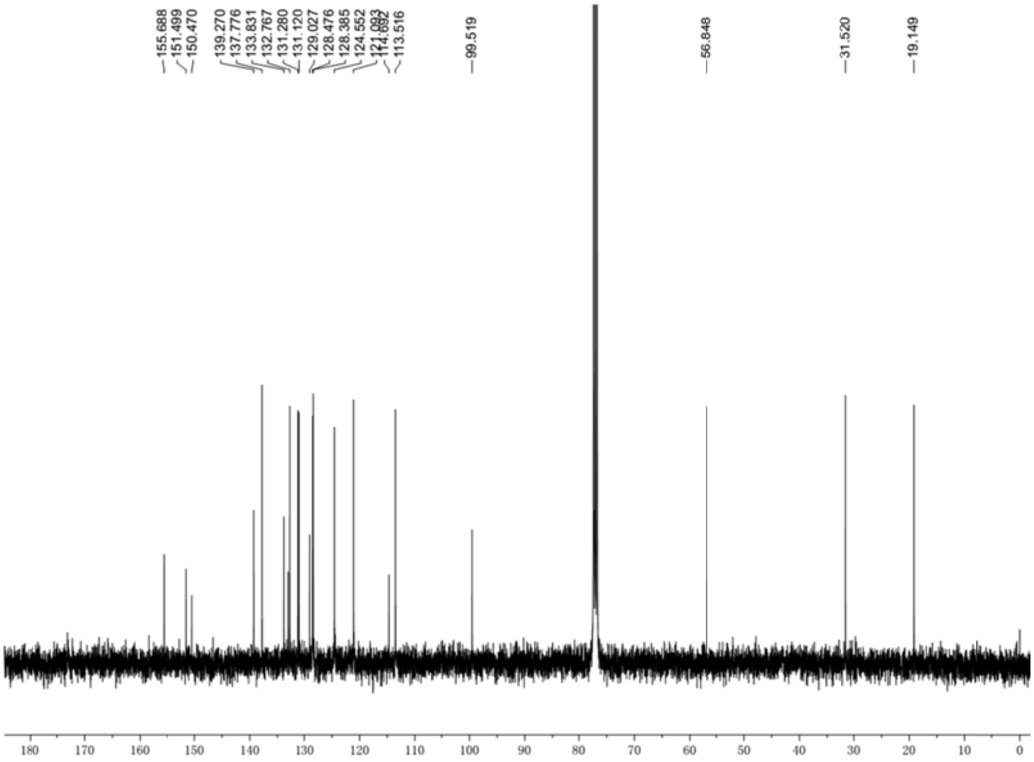

Supplementary Figure 31. ^{13}C NMR of the **6o**

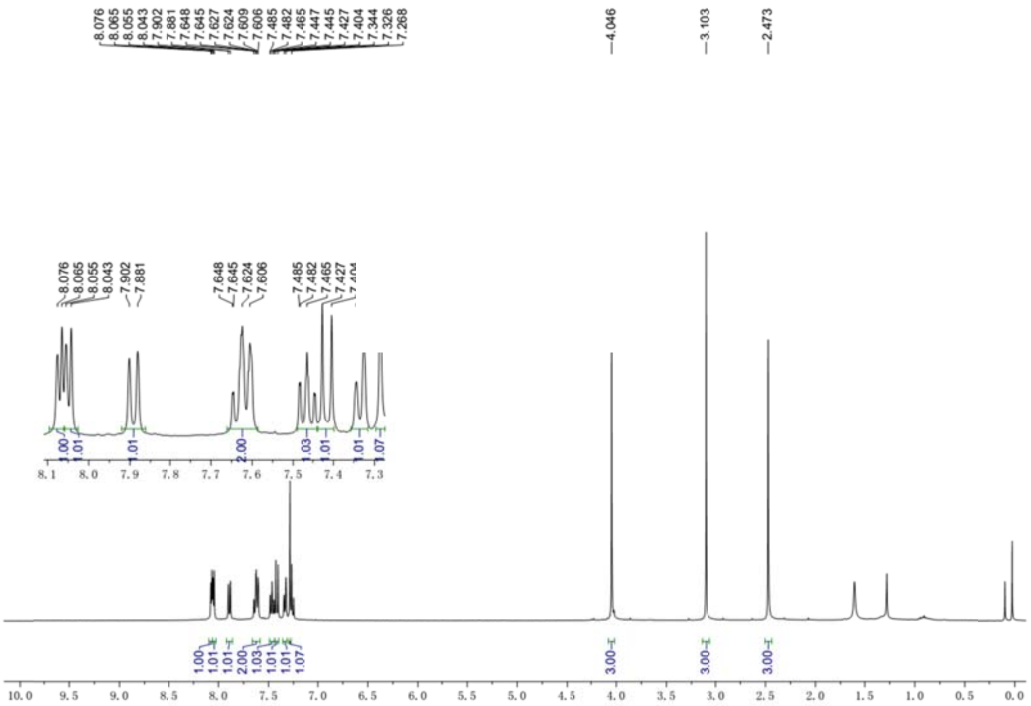

Supplementary Figure 32. ^{1}H NMR of the **6p**

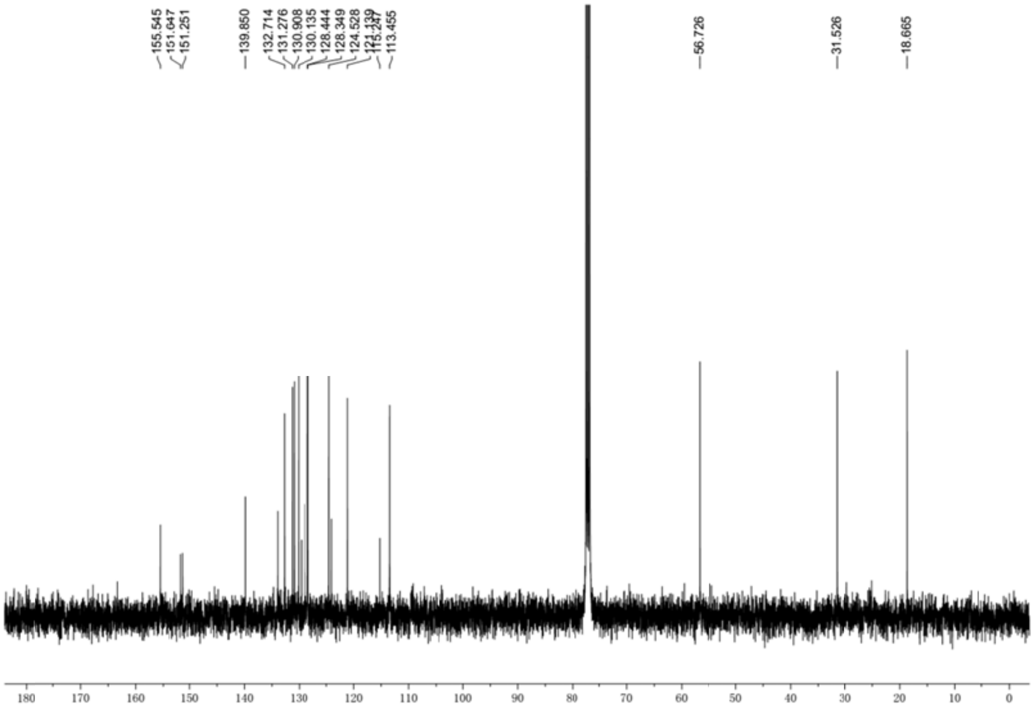

Supplementary Figure 33. ^{13}C NMR of the **6p**

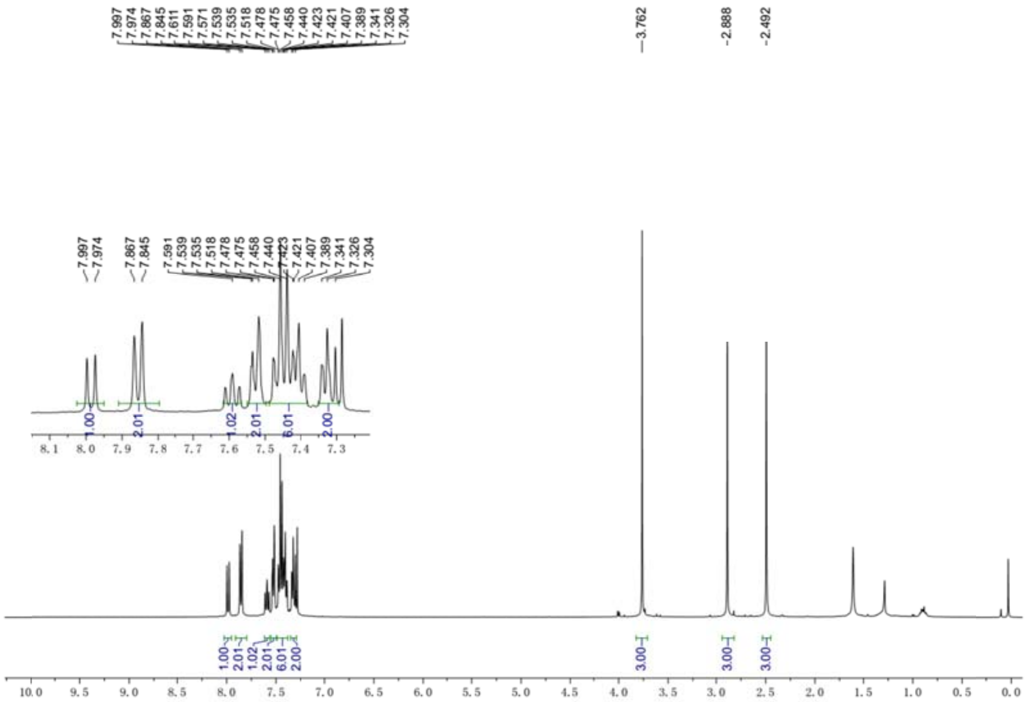

Supplementary Figure 34. ^{1}H NMR of the **6q**

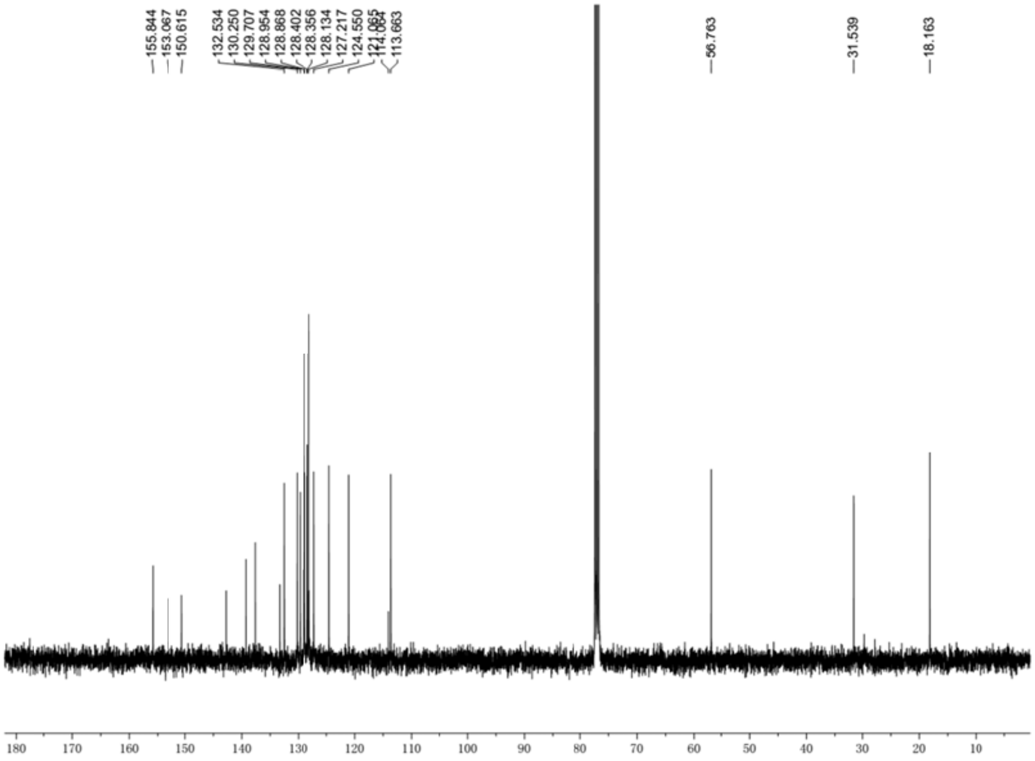

Supplementary Figure 35. ^{13}C NMR of the **6q**

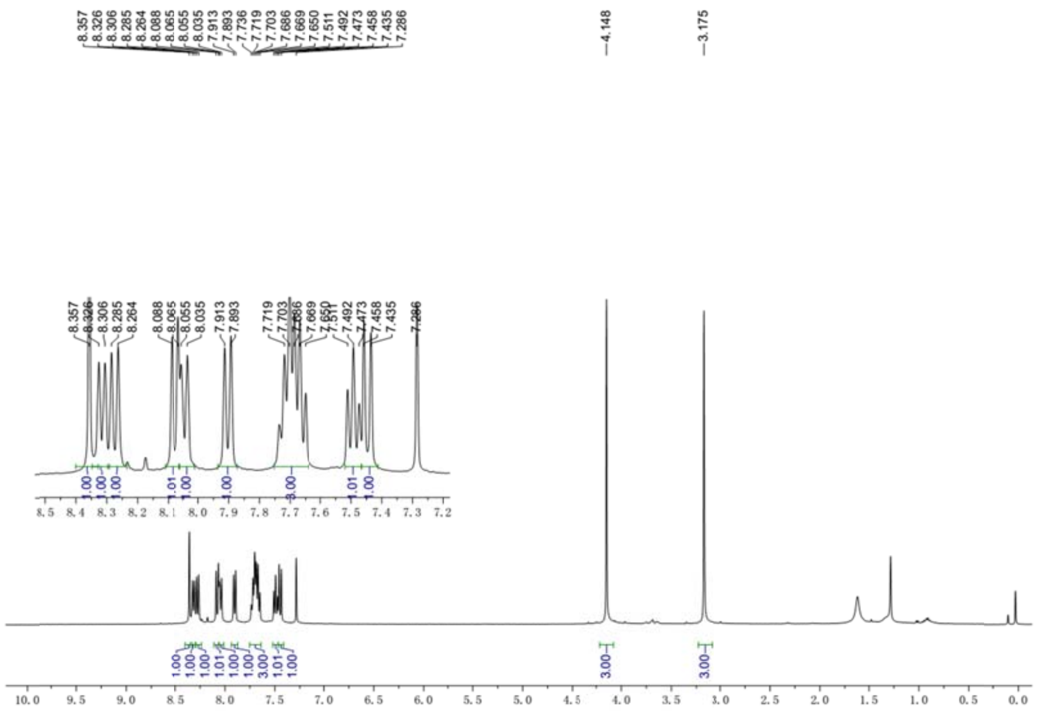

Supplementary Figure 36. ^{1}H NMR of the **6r**

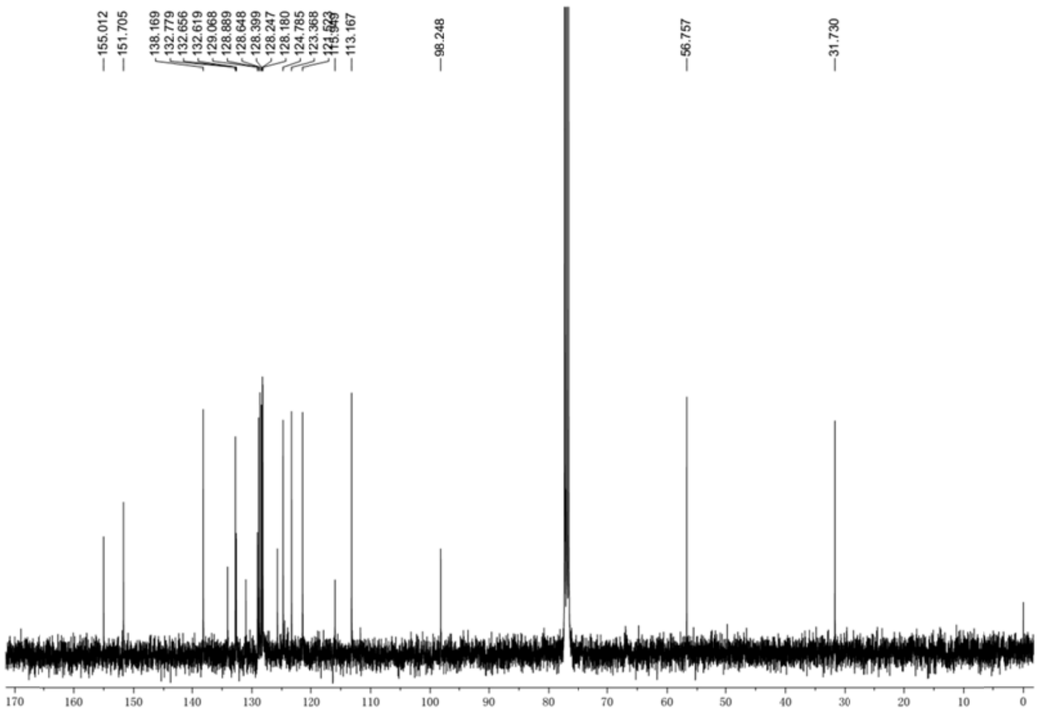

Supplementary Figure 37. ^{13}C NMR of the **6r**

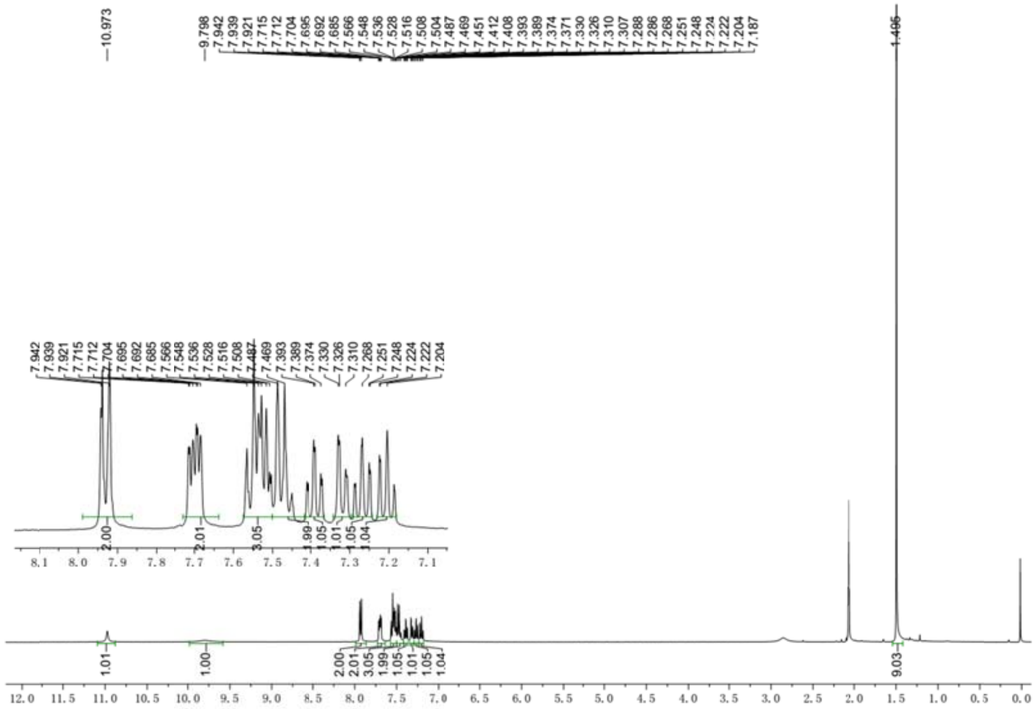

Supplementary Figure 38. ^{1}H NMR of the **5a**

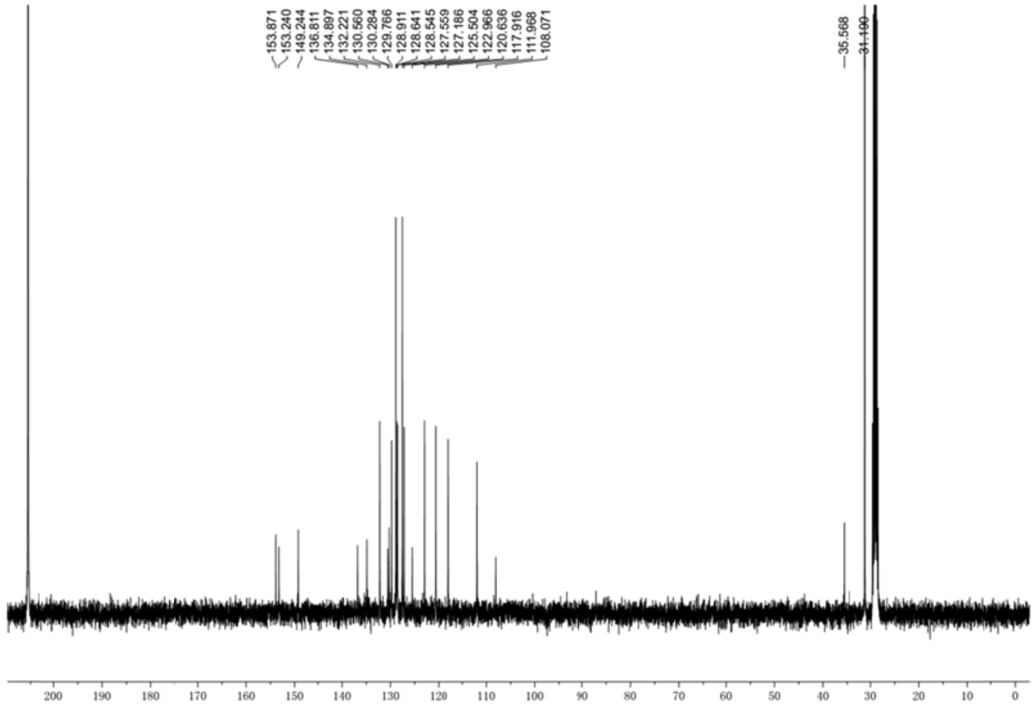

Supplementary Figure 39. ^{13}C NMR of the **5a**

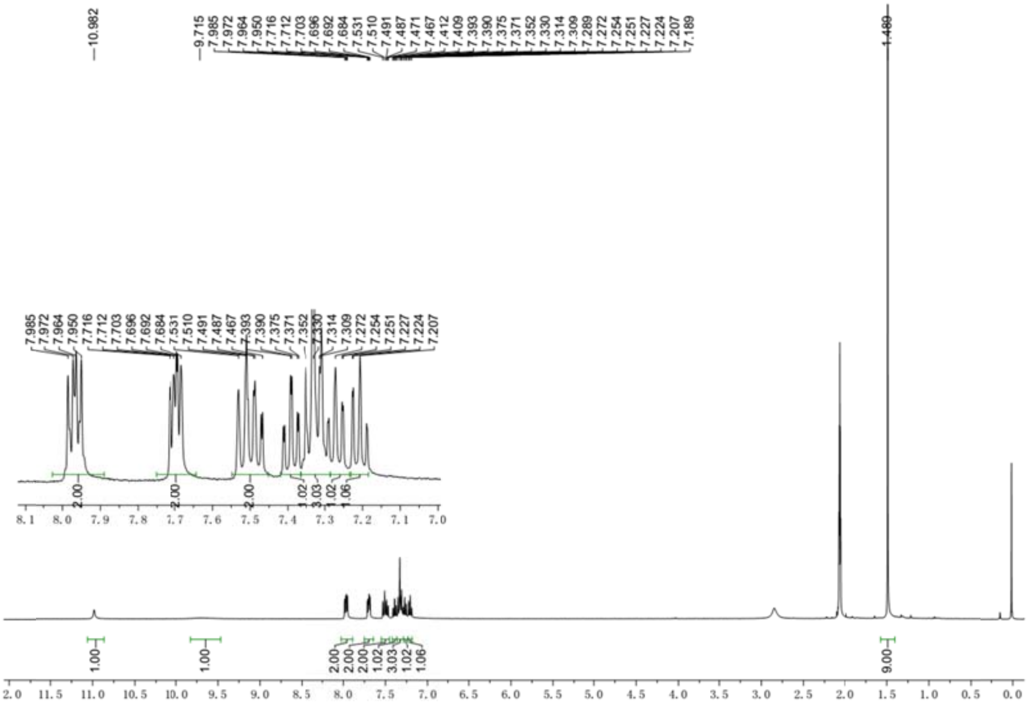

Supplementary Figure 40. ^{1}H NMR of the **5b**

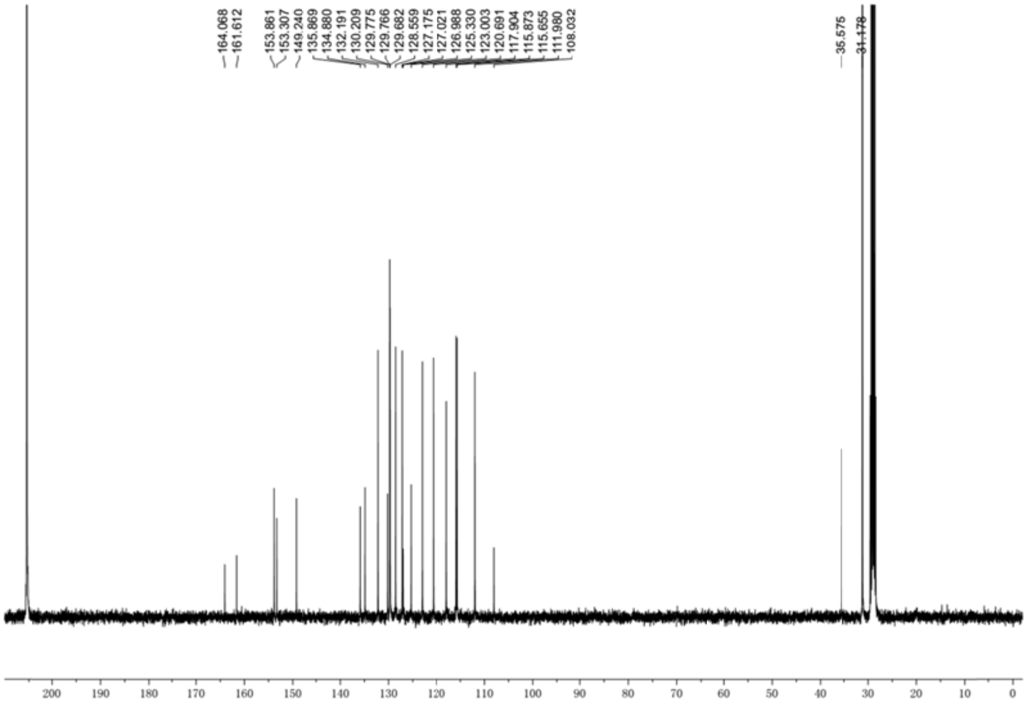

Supplementary Figure 41. ^{13}C NMR of the **5b**

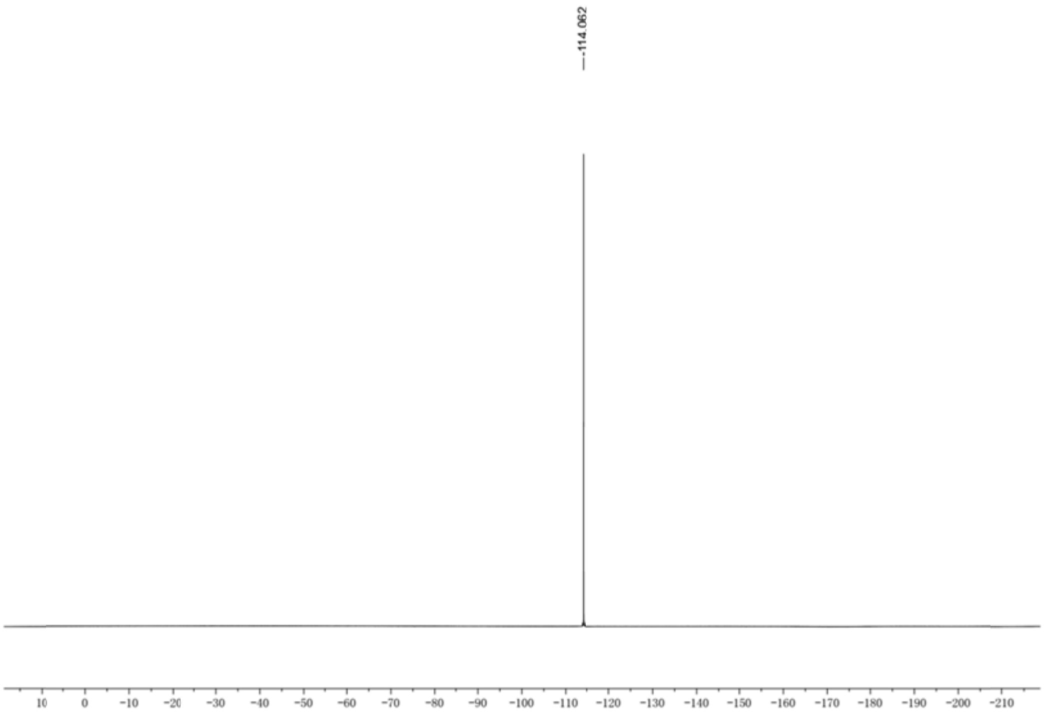

Supplementary Figure 42. ^{19}F NMR of the **5b**

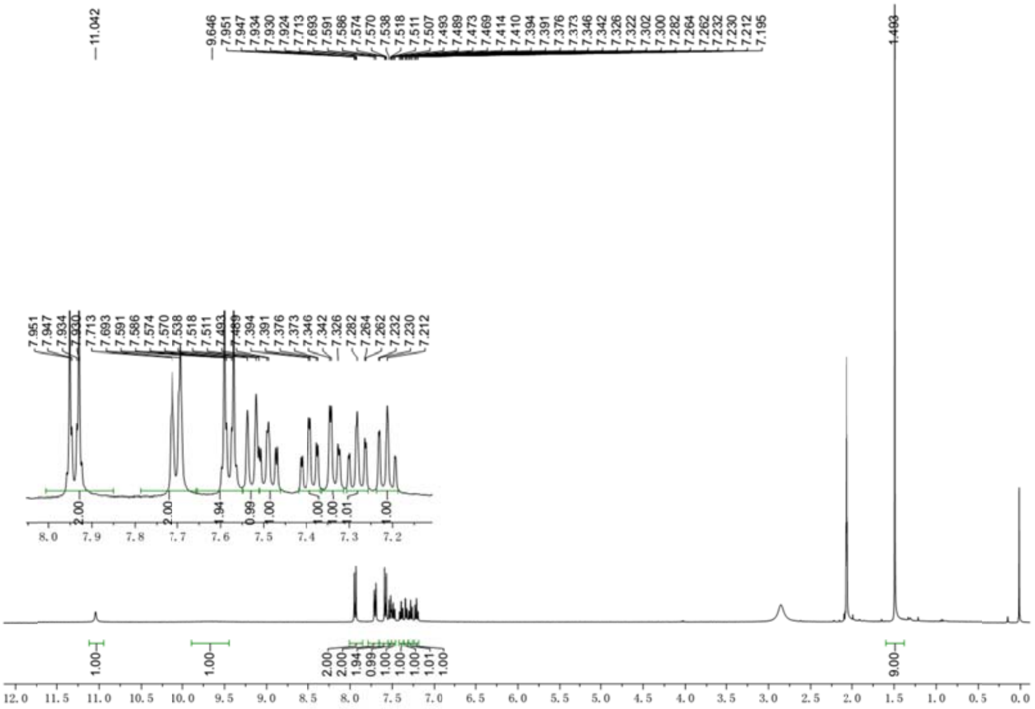

Supplementary Figure 43. ^{1}H NMR of the **5c**

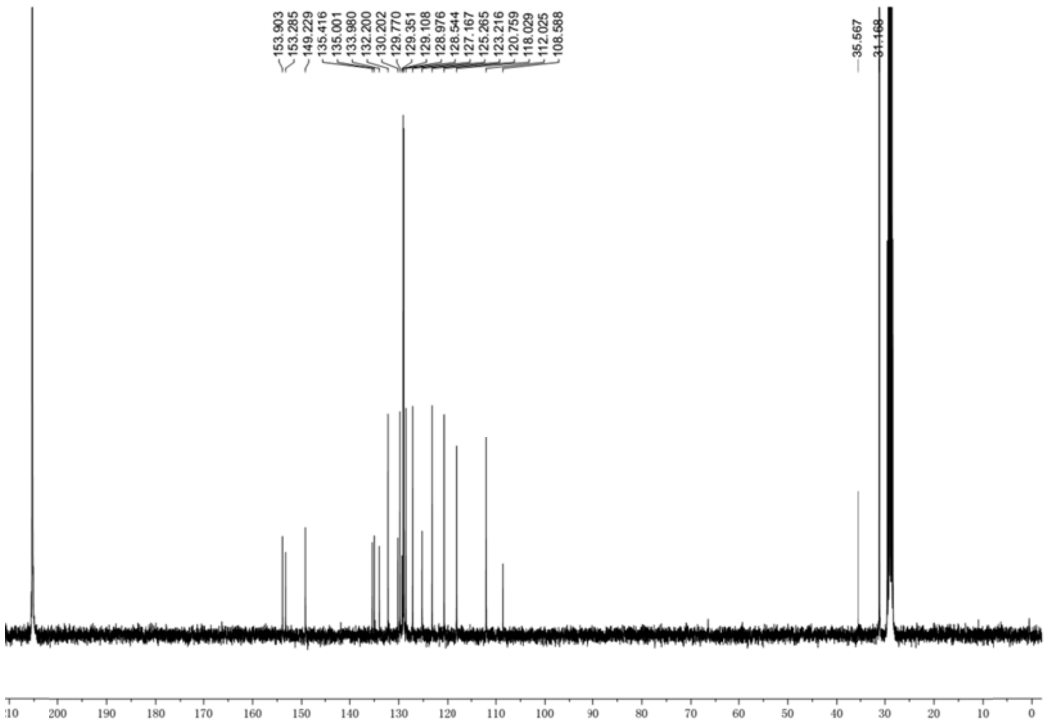

Supplementary Figure 44. ^{13}C NMR of the **5c**

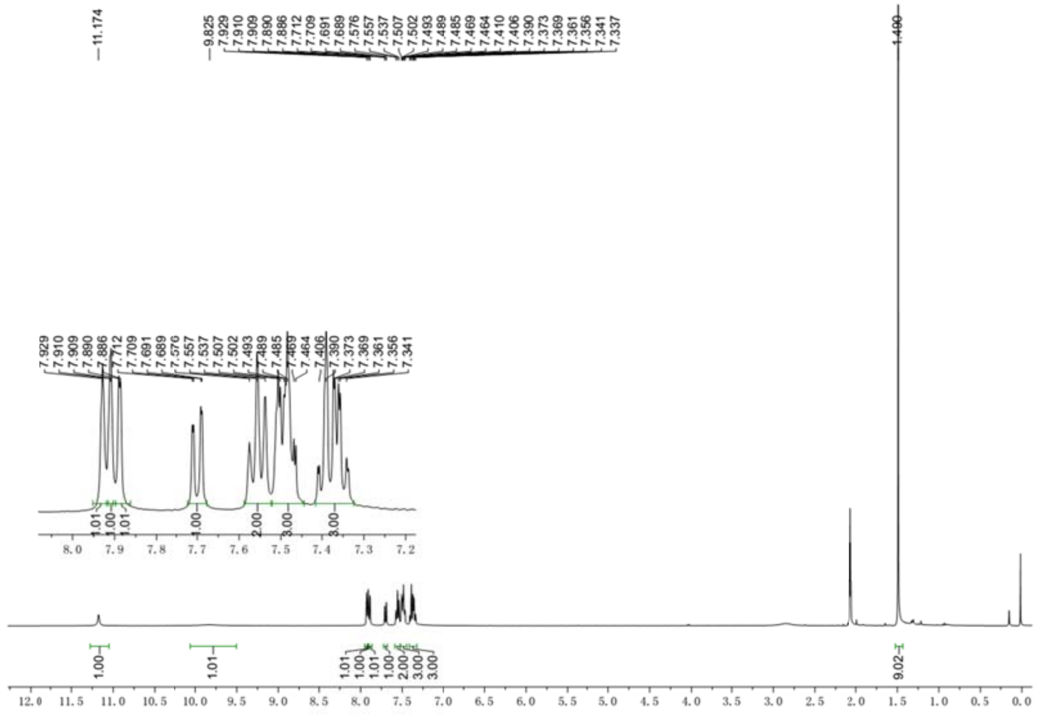

Supplementary Figure 45. ^{1}H NMR of the **5d**

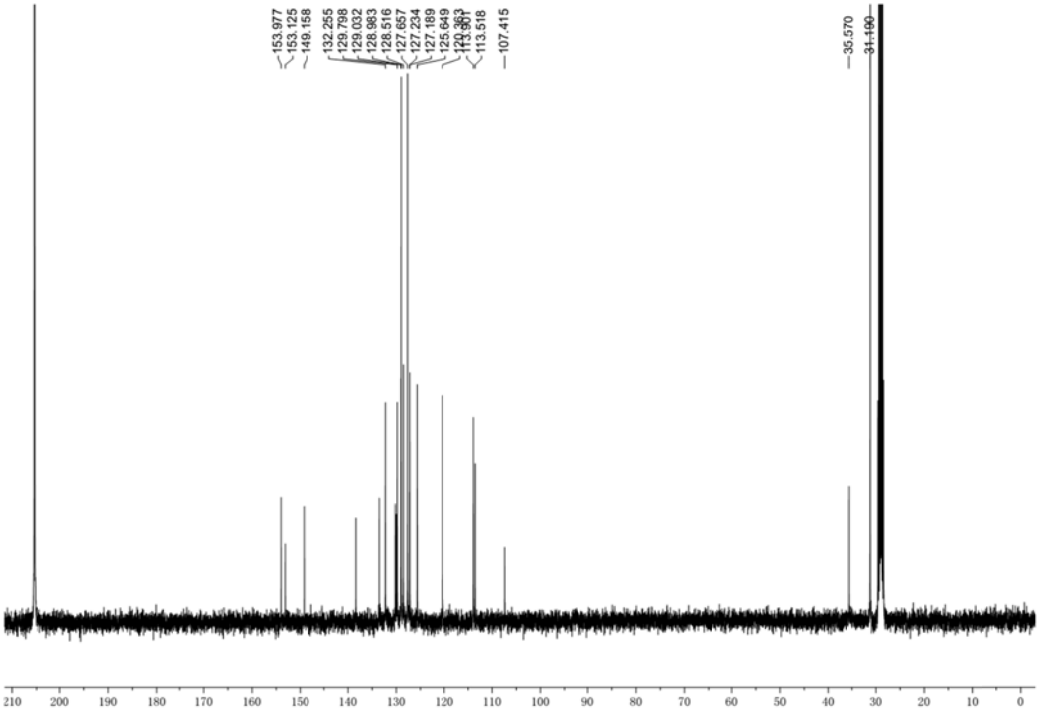

Supplementary Figure 46. ^{13}C NMR of the **5d**

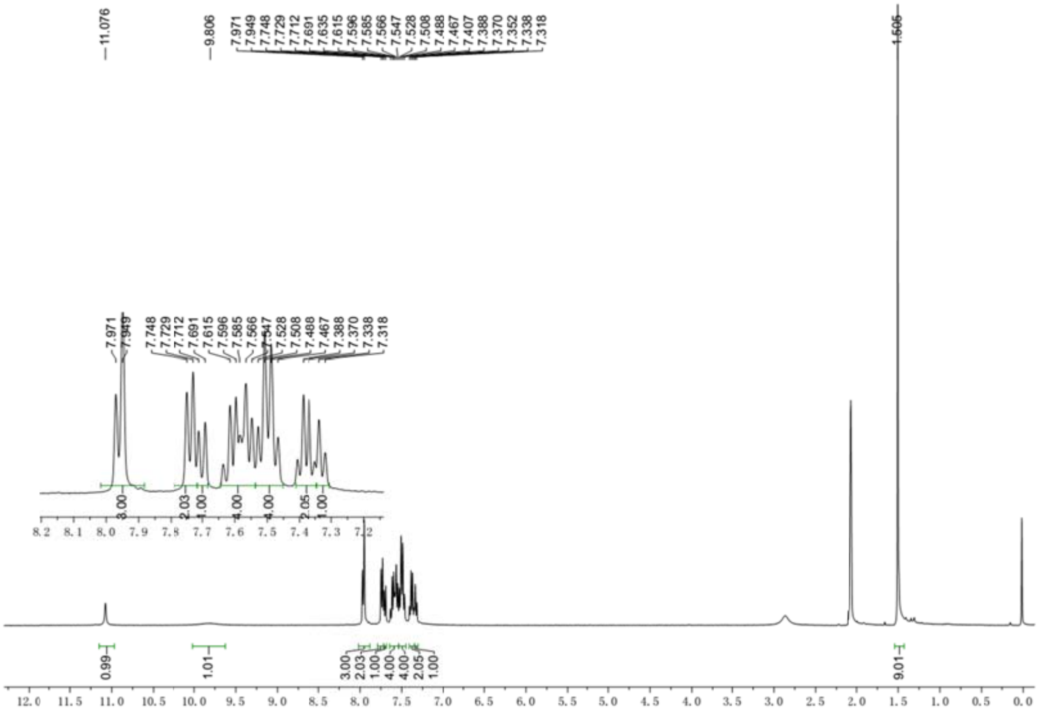

Supplementary Figure 47. ^{1}H NMR of the **5e**

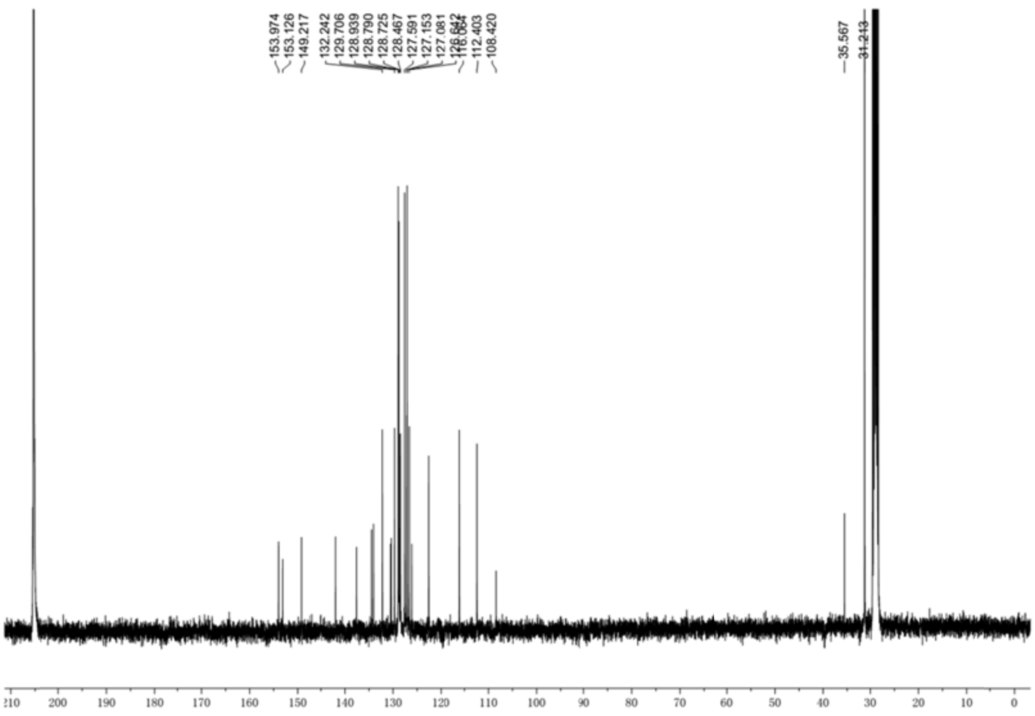

Supplementary Figure 48. ^{13}C NMR of the **5e**

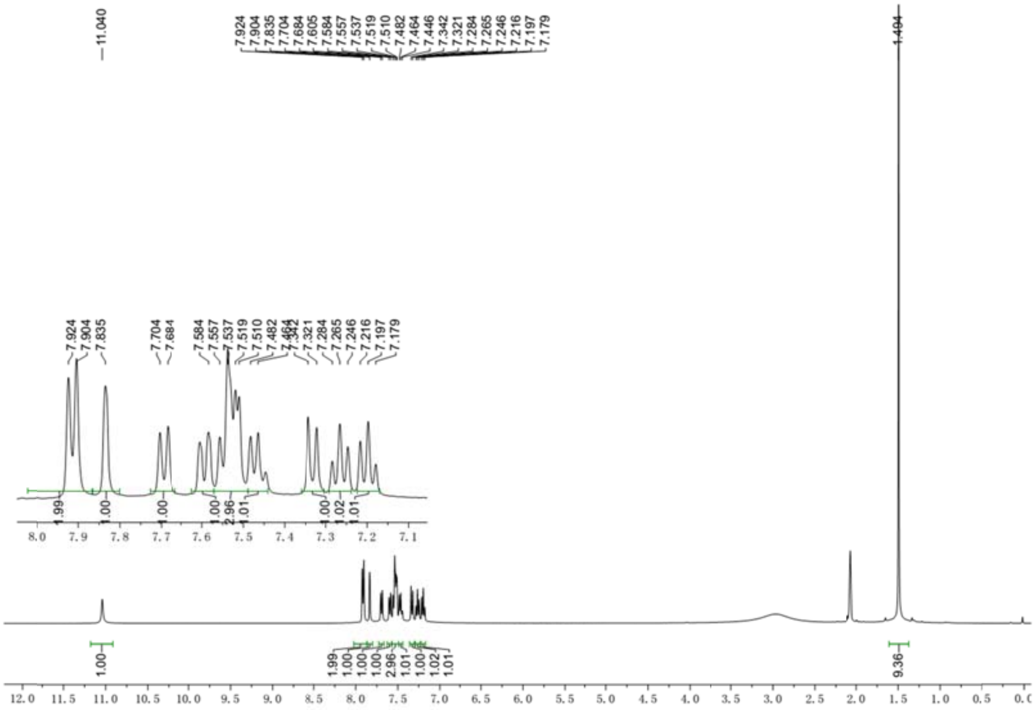

Supplementary Figure 49. ^{1}H NMR of the **5f**

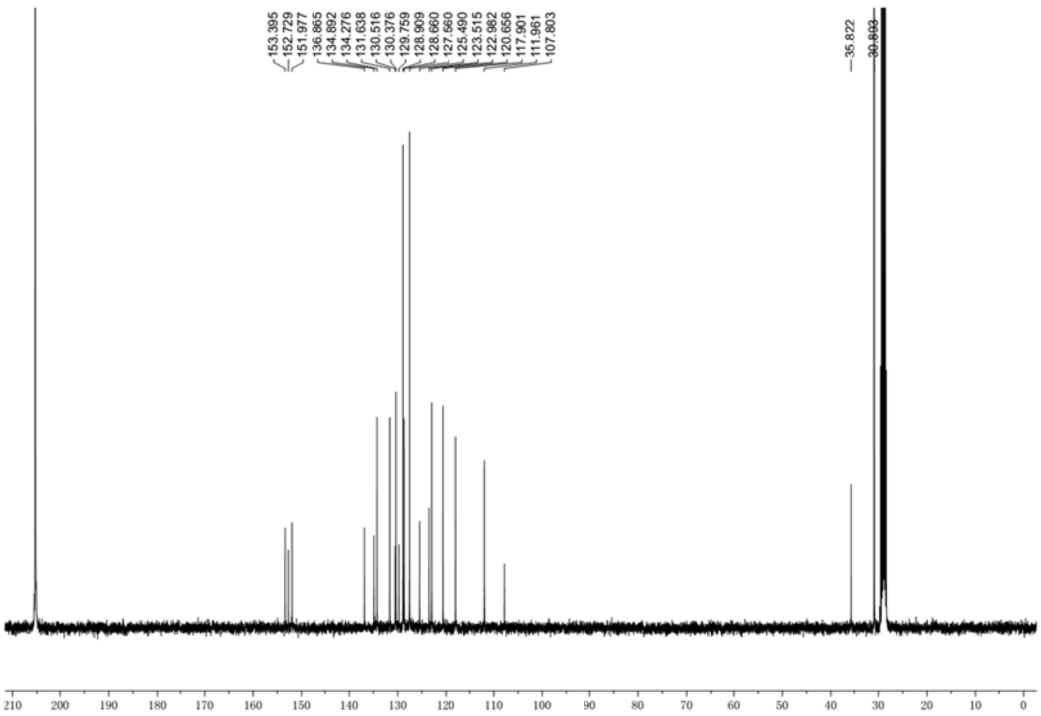

Supplementary Figure 50. ^{13}C NMR of the **5f**

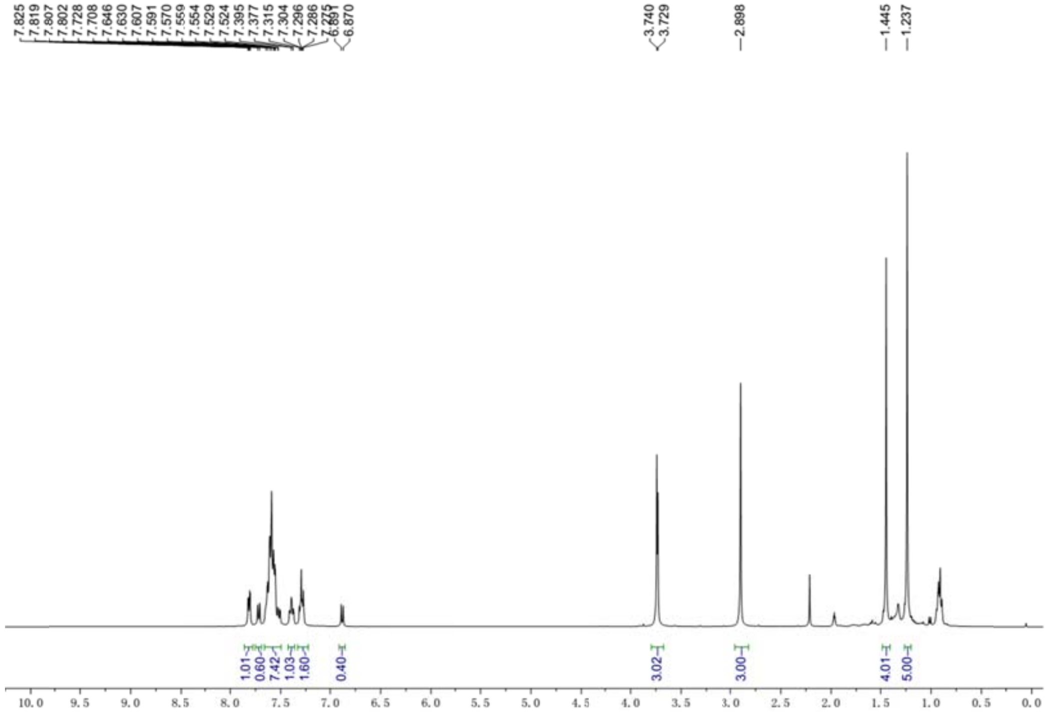

Supplementary Figure 51. ^{1}H NMR of the **7f**

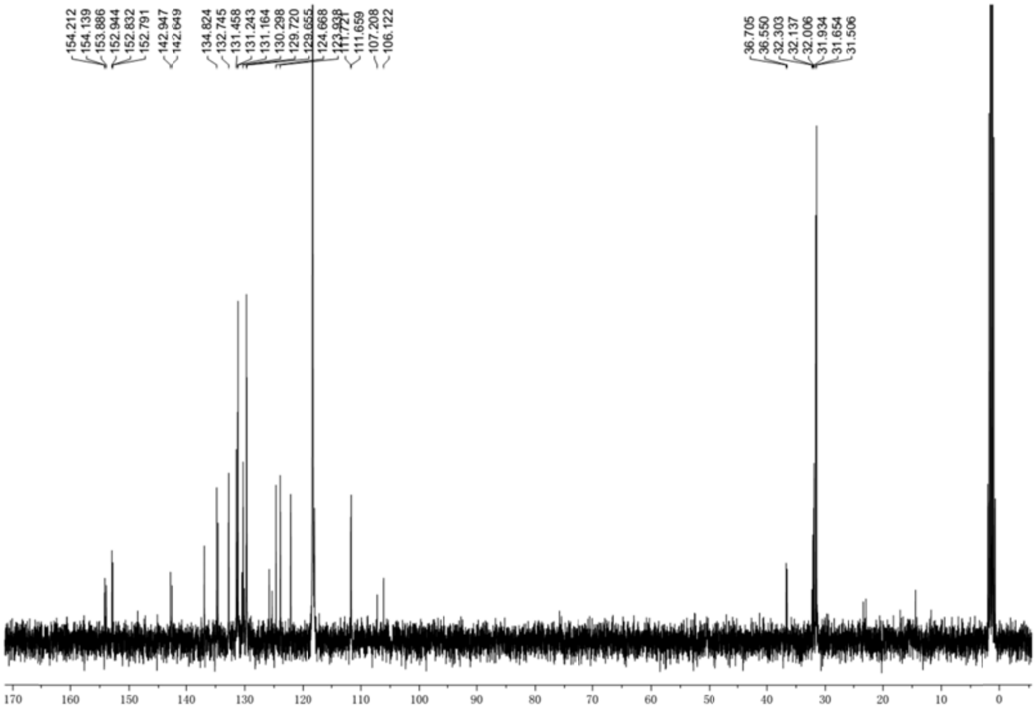

Supplementary Figure 52. ^{13}C NMR of the **7f**

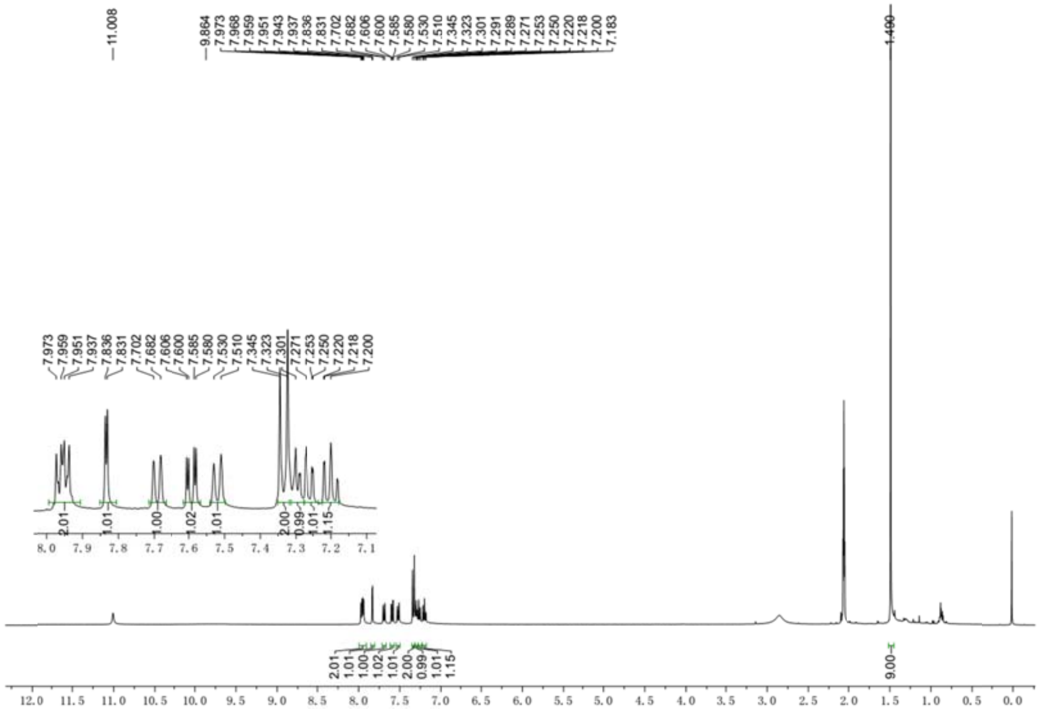

Supplementary Figure 53. ^{1}H NMR of the **5g**

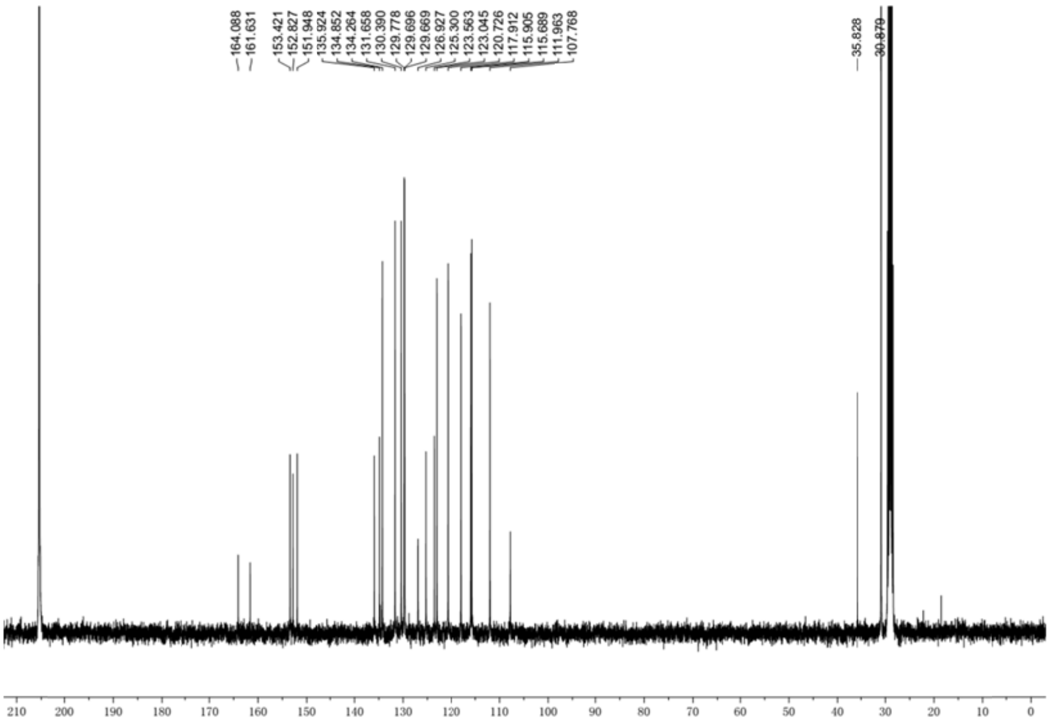

Supplementary Figure 54. ^{13}C NMR of the **5g**

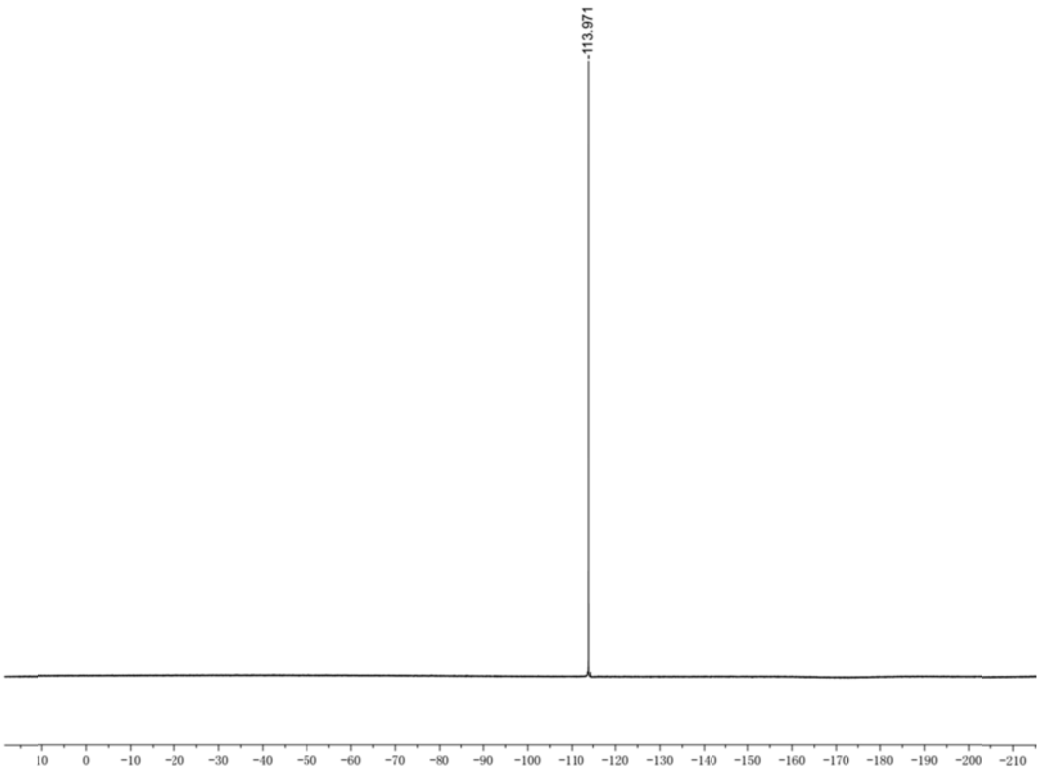

Supplementary Figure 55. ^{19}F NMR of the **5g**

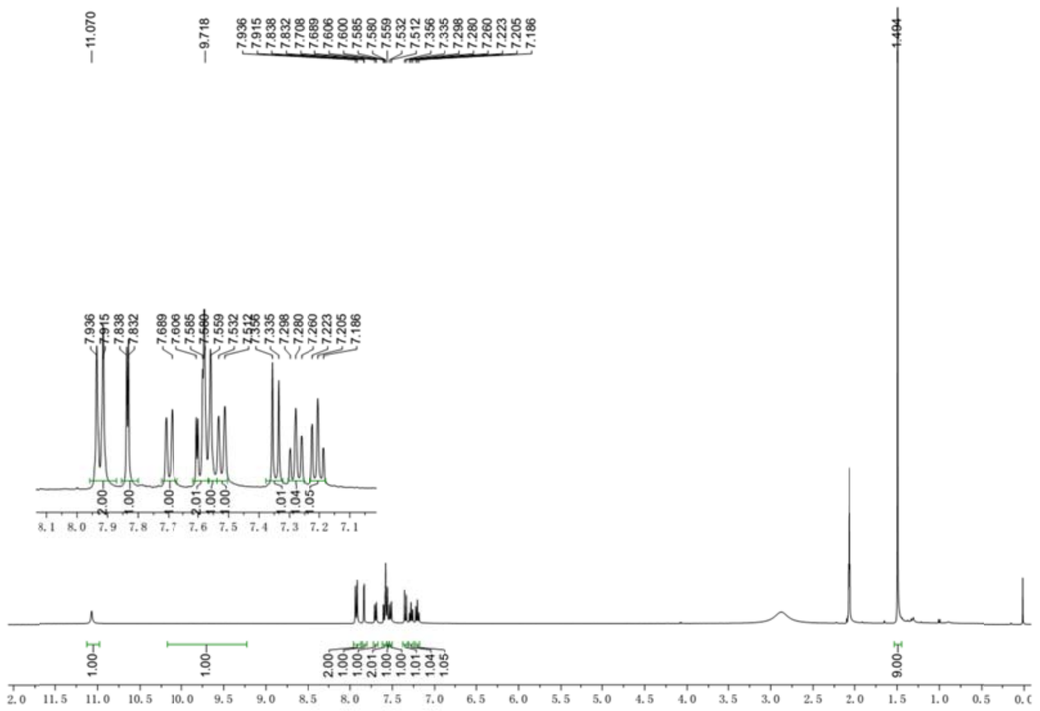

Supplementary Figure 56. ^{1}H NMR of the **5h**

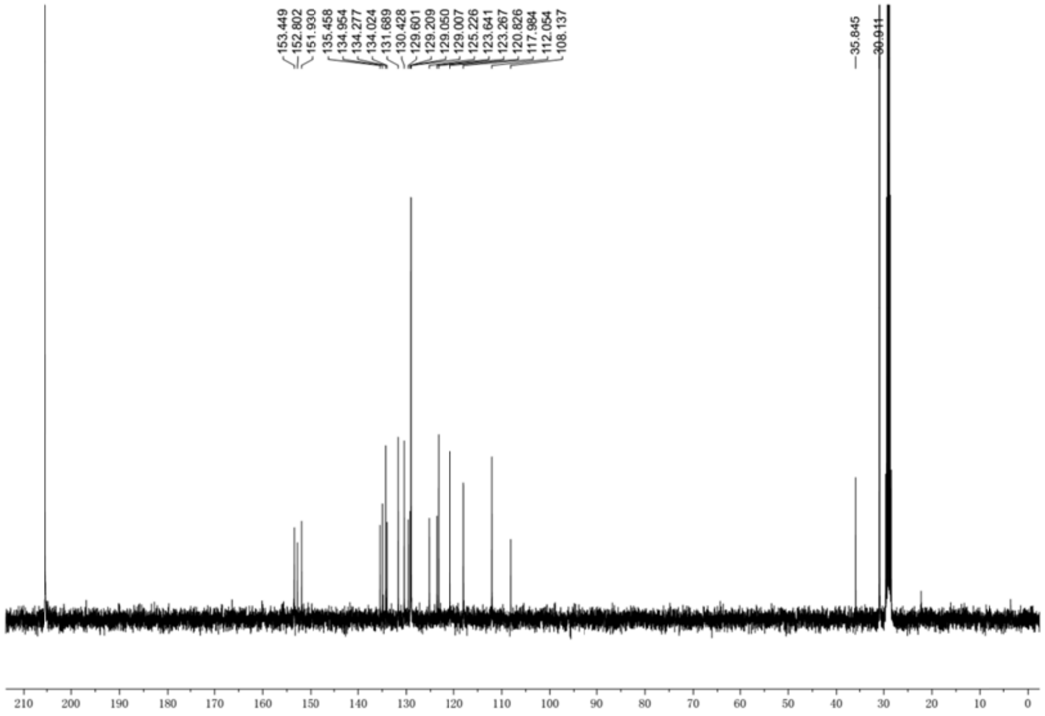

Supplementary Figure 57. ^{13}C NMR of the **5h**

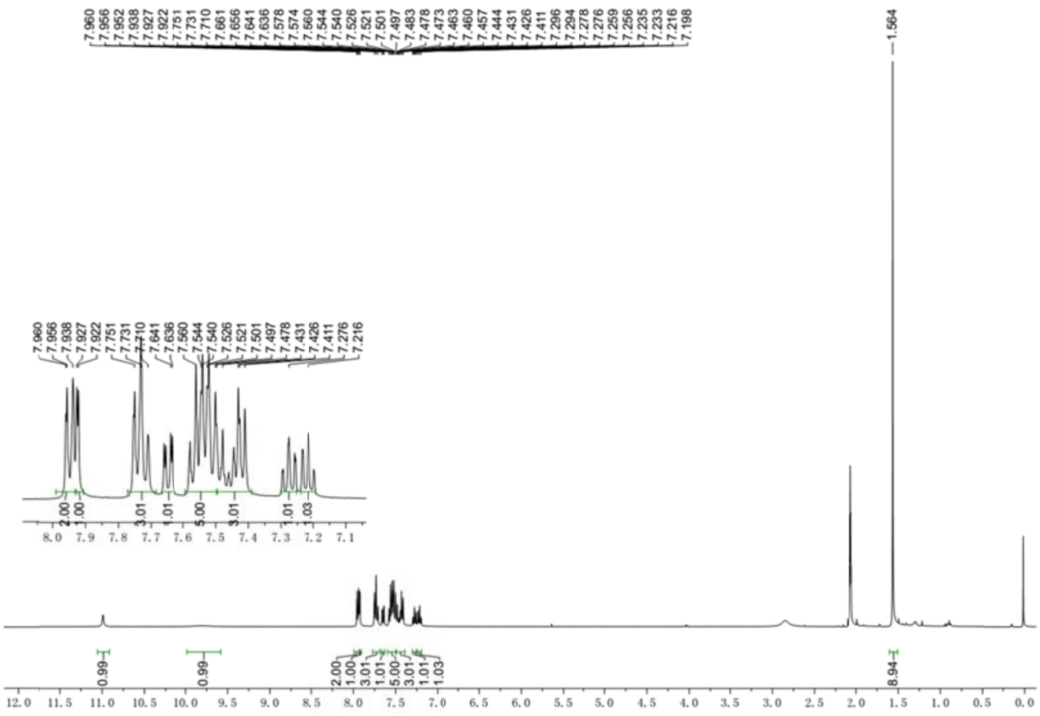

Supplementary Figure 58. ^{1}H NMR of the **5i**

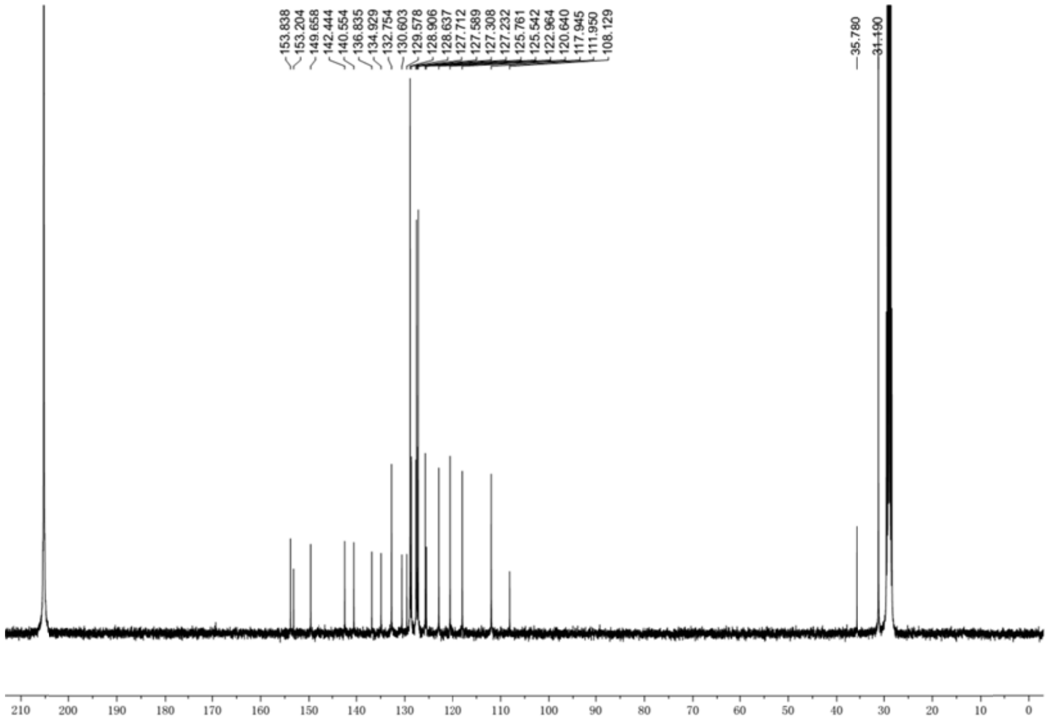

Supplementary Figure 59. ^{13}C NMR of the **5i**

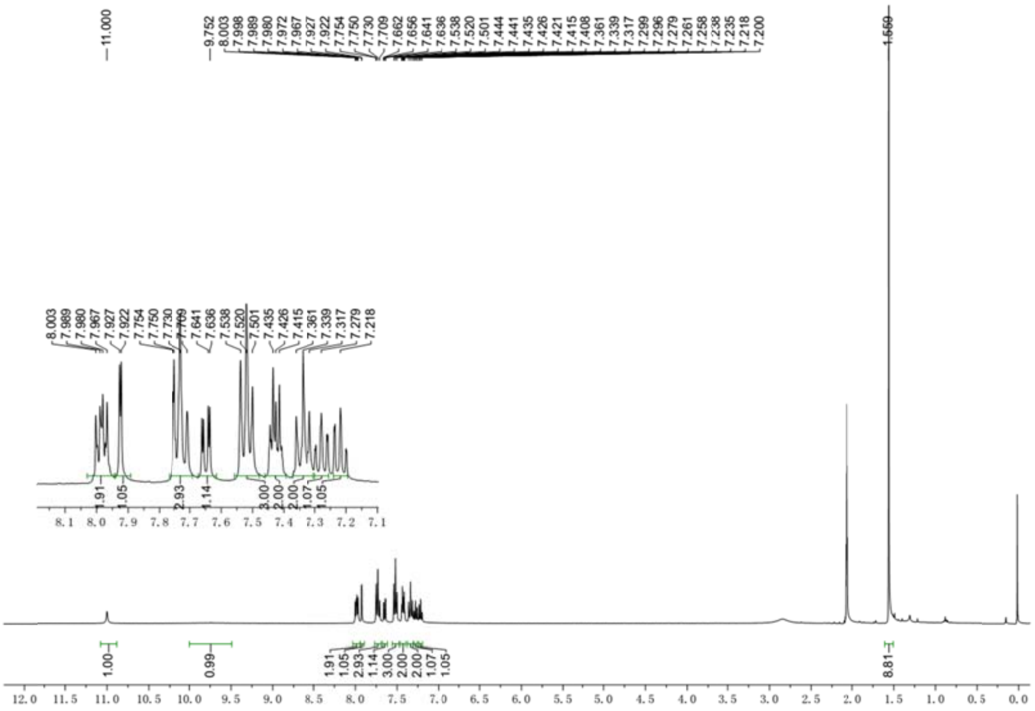

Supplementary Figure 60. ^{1}H NMR of the **5j**

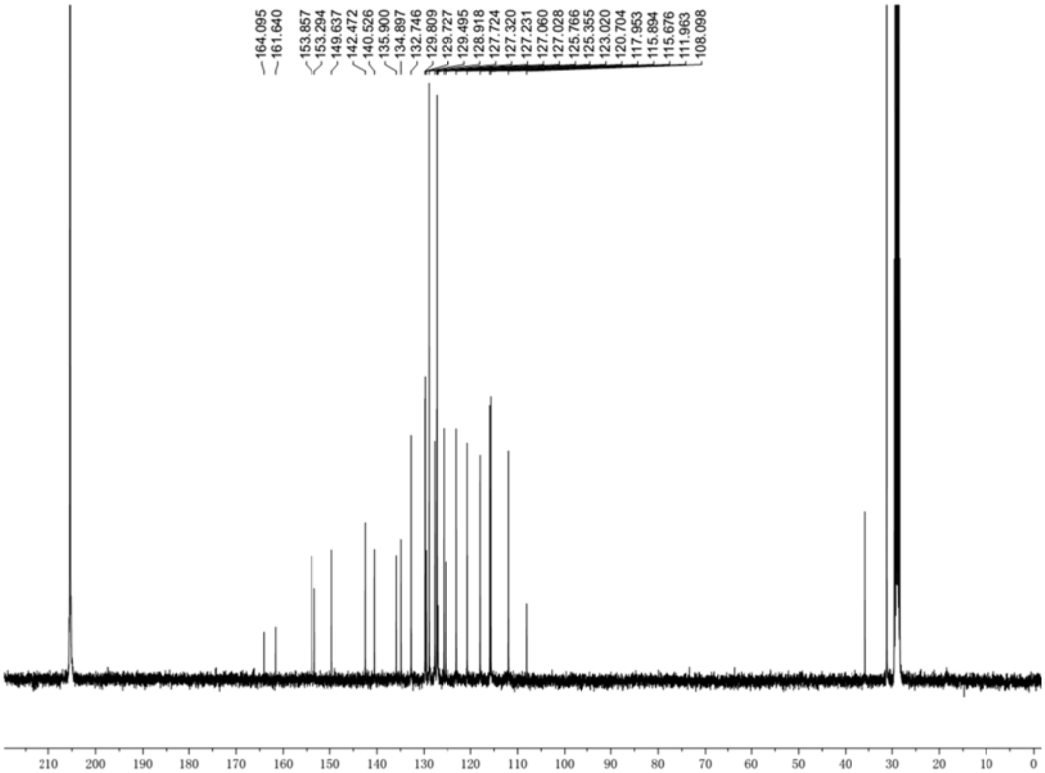

Supplementary Figure 61. ^{13}C NMR of the **5j**

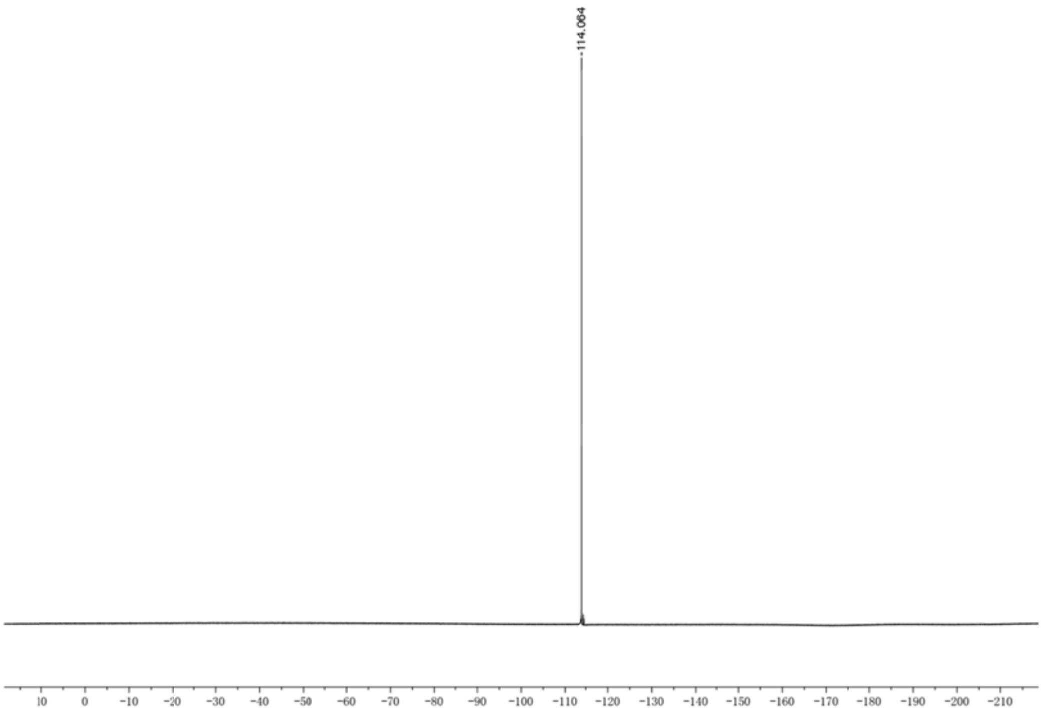

Supplementary Figure 62. ^{19}F NMR of the **5j**

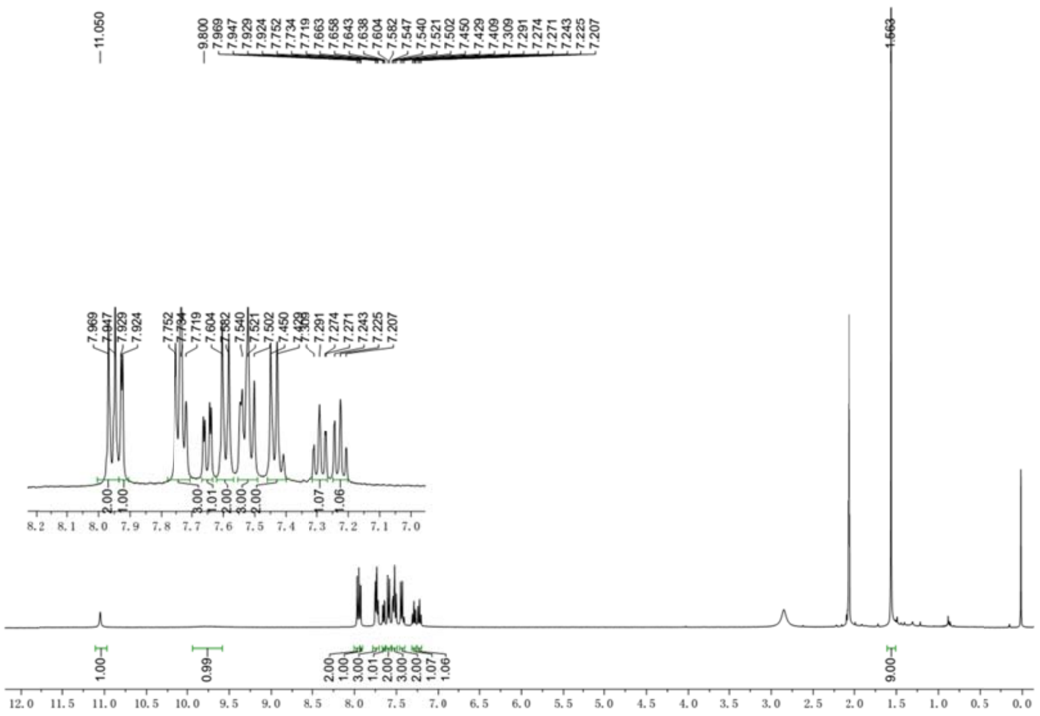

Supplementary Figure 63. ^{1}H NMR of the **5k**

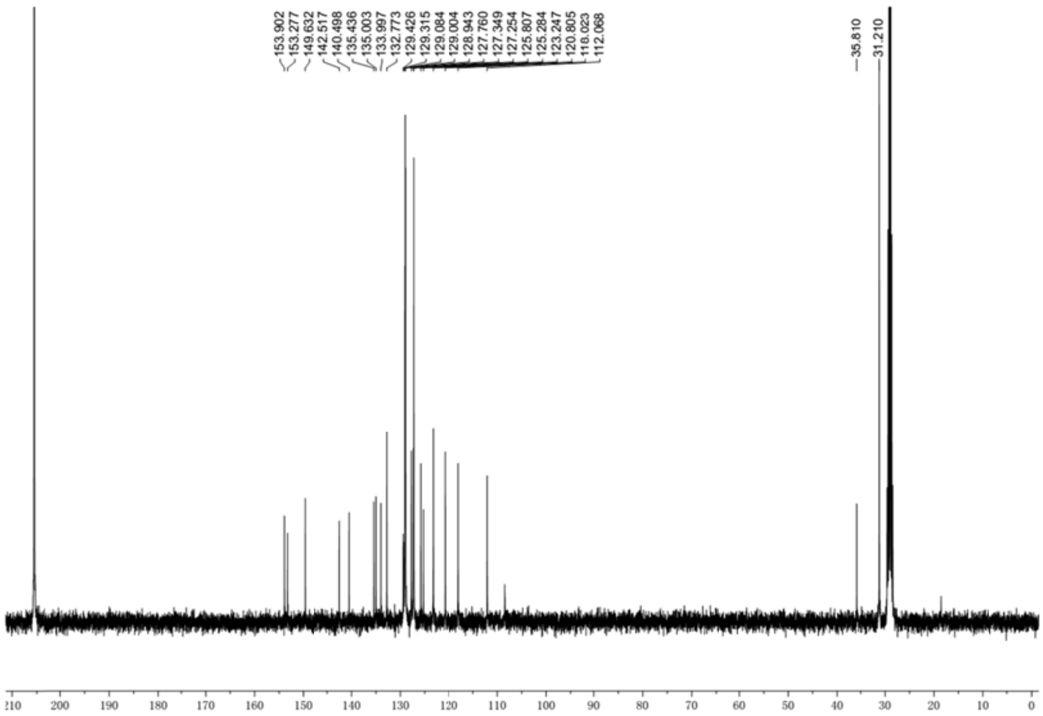

Supplementary Figure 64. ^{13}C NMR of the **5k**

In8

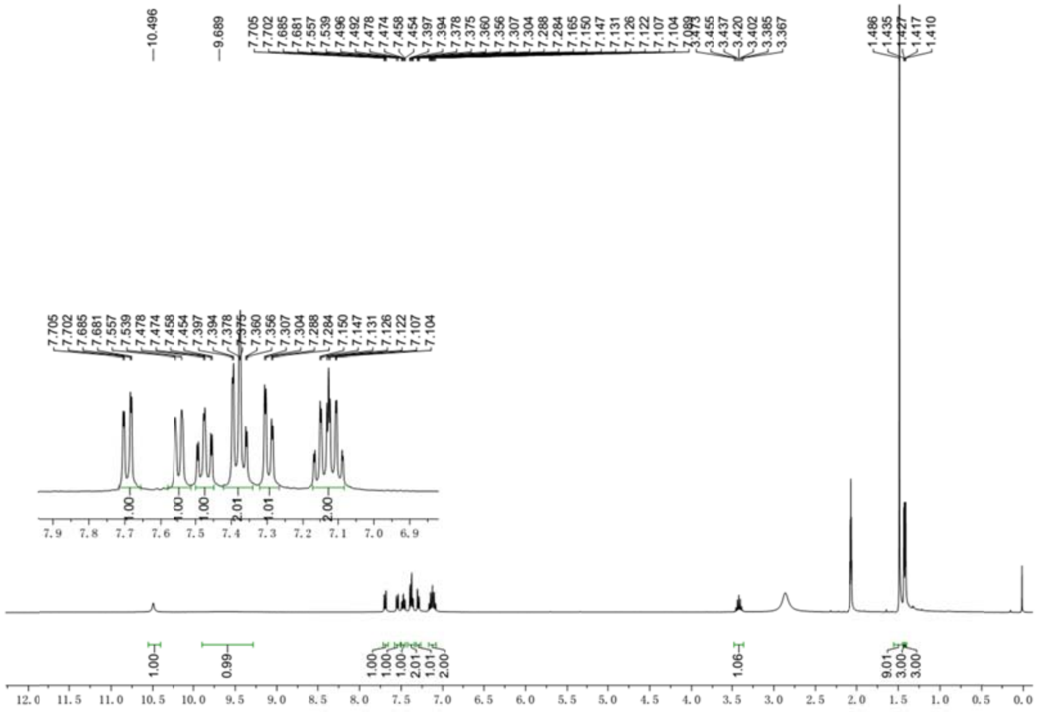

Supplementary Figure 65. ^{1}H NMR of the **5l**

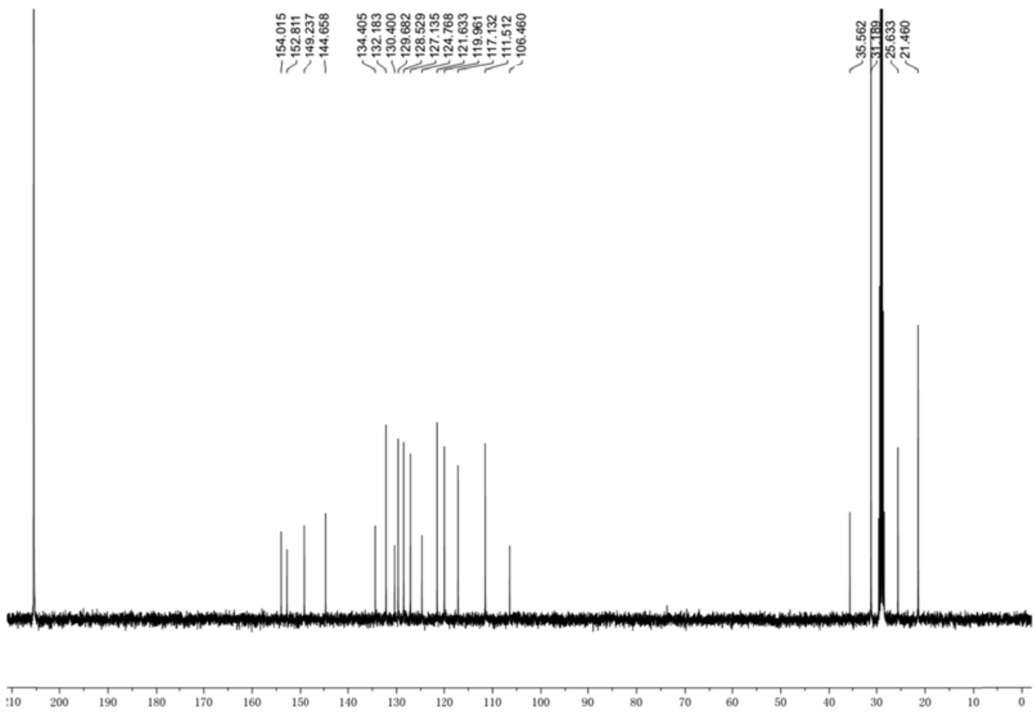

Supplementary Figure 66. ^{13}C NMR of the **5l**

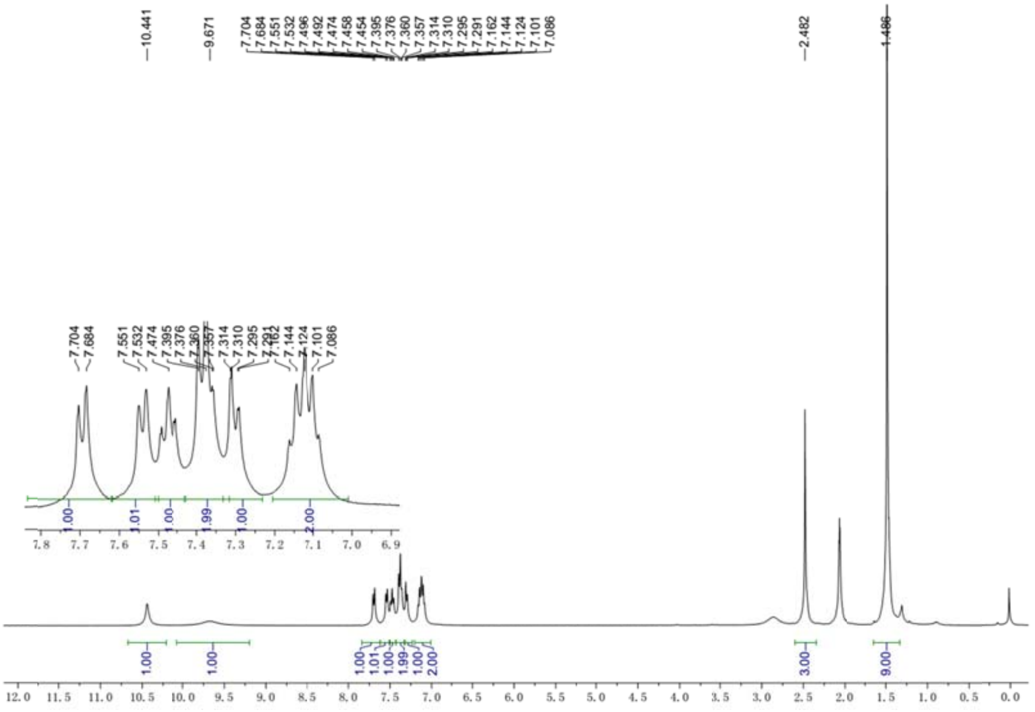

Supplementary Figure 67. ^{1}H NMR of the **5m**

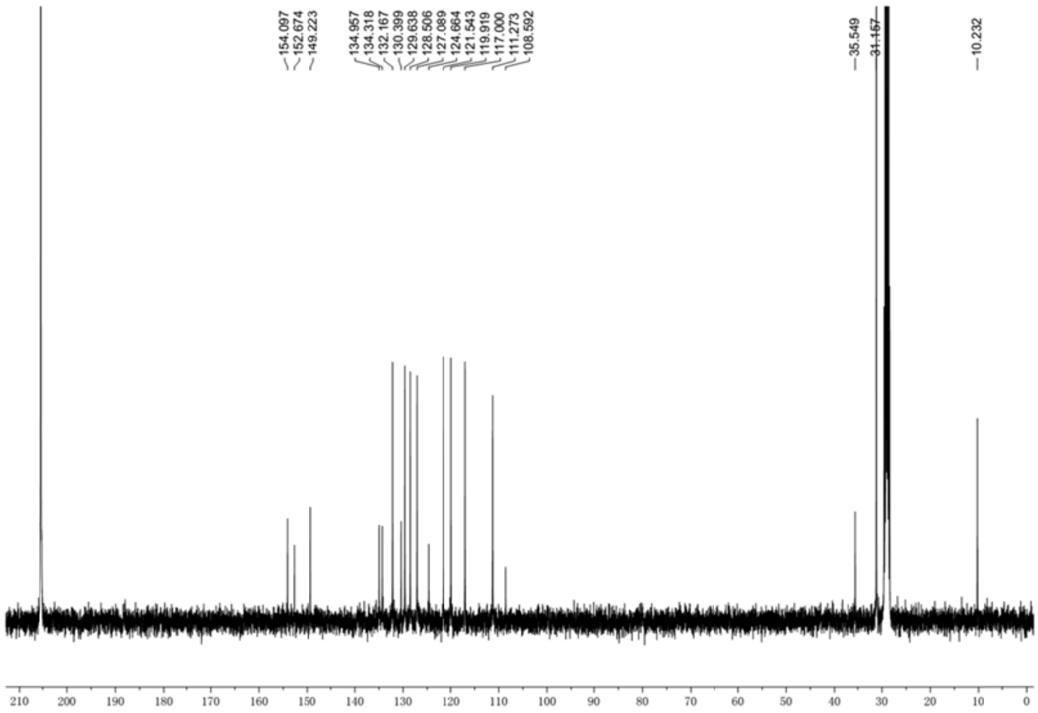

Supplementary Figure 68. ^{13}C NMR of the **5m**

Supplementary Figures 69-101 (HPLC traces)

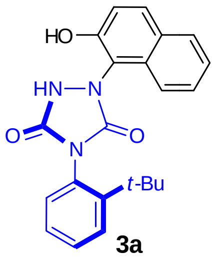

3a

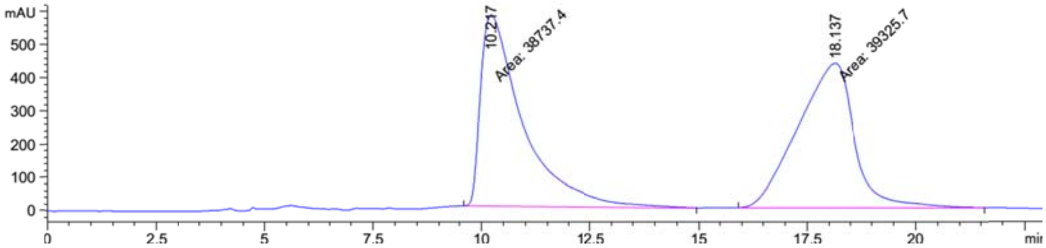

Signal 2: DAD1 B, Sig=230,4 Ref=360,100

Peak #	RetTime [min]	Type	Width [min]	Area [mAU*s]	Height [mAU]	Area %
1	10.217	MM	1.1214	3.87374e4	575.73523	49.6232
2	18.137	MM	1.5066	3.93257e4	435.04480	50.3768

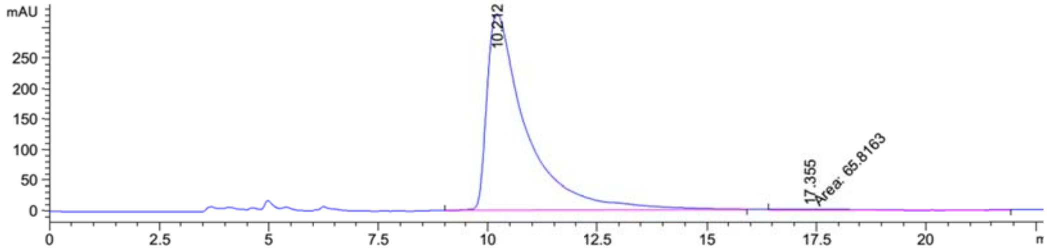

Signal 2: DAD1 B, Sig=230,4 Ref=360,100

Peak #	RetTime [min]	Type	Width [min]	Area [mAU*s]	Height [mAU]	Area %
1	10.212	BB	0.8866	1.99480e4	321.17990	99.6711
2	17.355	MM	1.5170	65.81625	7.23091e-1	0.3289

Supplementary Figure 69. HPLC traces for racemic and chiral product **3a**

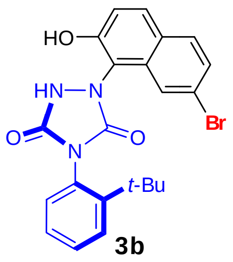

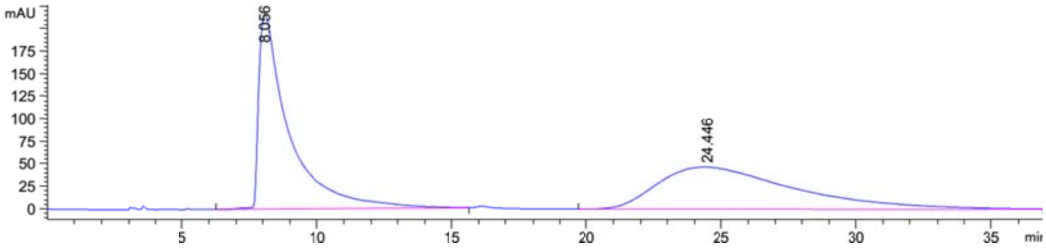

Signal 3: DAD1 D, Sig=230,4 Ref=360,100

Peak #	RetTime [min]	Type	Width [min]	Area [mAU*s]	Height [mAU]	Area %
1	8.056	BB	1.0901	1.69818e4	215.79517	50.7845
2	24.446	BBA	4.1399	1.64571e4	46.89938	49.2155

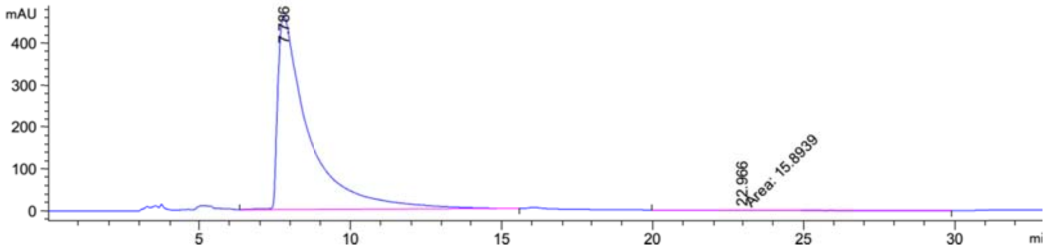

Signal 3: DAD1 D, Sig=230,4 Ref=360,100

Peak #	RetTime [min]	Type	Width [min]	Area [mAU*s]	Height [mAU]	Area %
1	7.786	BB	0.9759	3.31240e4	462.32867	99.9520
2	22.966	MM	0.8189	15.89386	3.23473e-1	0.0480

Supplementary Figure 70. HPLC traces for racemic and chiral product **3b**

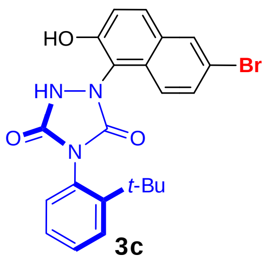

3c

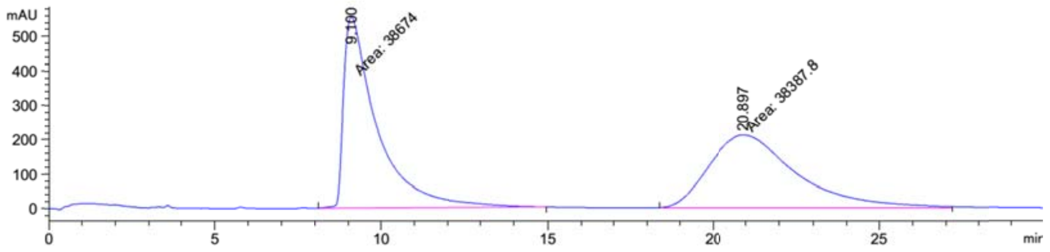

Signal 3: DAD1 D, Sig=230,4 Ref=360,100

Peak #	RetTime [min]	Type	Width [min]	Area [mAU*s]	Height [mAU]	Area %
1	9.100	MM	1.1618	3.86740e4	554.79968	50.1857
2	20.897	MM	3.0355	3.83878e4	210.77226	49.8143

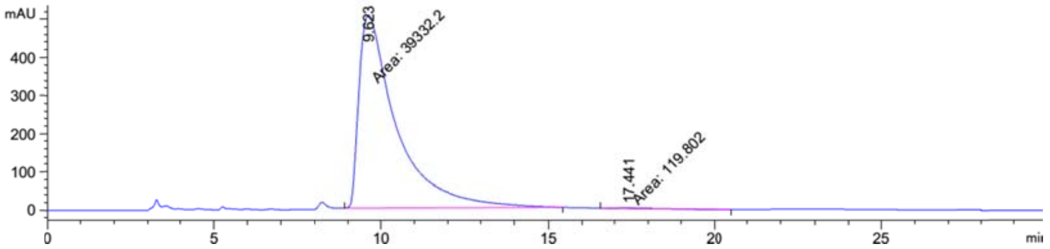

Signal 3: DAD1 D, Sig=230,4 Ref=360,100

Peak #	RetTime [min]	Type	Width [min]	Area [mAU*s]	Height [mAU]	Area %
1	9.623	MM	1.2974	3.93322e4	505.28503	99.6963
2	17.441	MM	1.2452	119.80234	1.60359	0.3037

Supplementary Figure 71. HPLC traces for racemic and chiral product **3c**

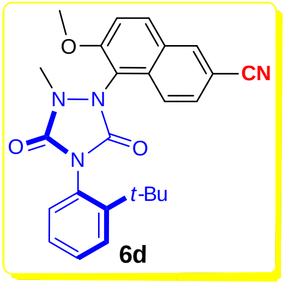

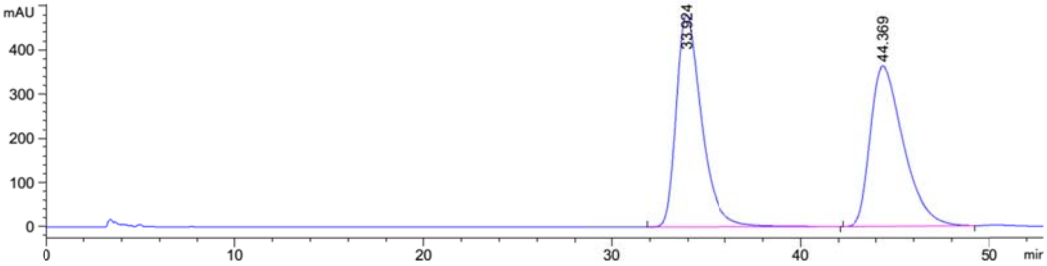

Signal 5: DAD1 E, Sig=240,4 Ref=360,100

Peak #	RetTime [min]	Type	Width [min]	Area [mAU*s]	Height [mAU]	Area %
1	33.924	BB	1.4242	4.45834e4	479.73822	50.3996
2	44.369	BB	1.8374	4.38764e4	362.17020	49.6004

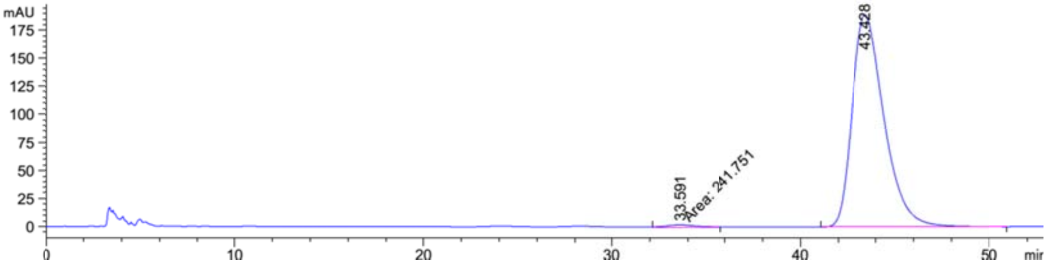

Signal 5: DAD1 E, Sig=240,4 Ref=360,100

Peak #	RetTime [min]	Type	Width [min]	Area [mAU*s]	Height [mAU]	Area %
1	33.591	MM	1.7978	241.75148	2.24116	1.0675
2	43.428	BB	1.7738	2.24057e4	189.44385	98.9325

Supplementary Figure 72. HPLC traces for racemic and chiral product **6d (from 3d)**

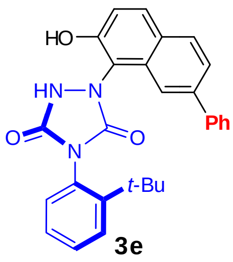

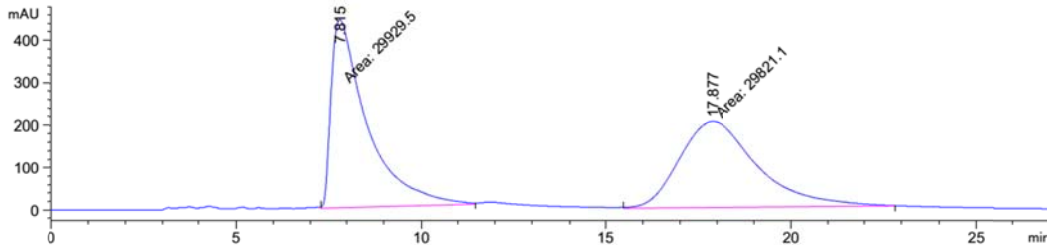

Signal 1: DAD1 A, Sig=254,4 Ref=360,100

Peak #	RetTime [min]	Type	Width [min]	Area [mAU*s]	Height [mAU]	Area %
1	7.815	MM	1.1215	2.99295e4	444.79727	50.0907
2	17.877	MM	2.4381	2.98211e4	203.85718	49.9093

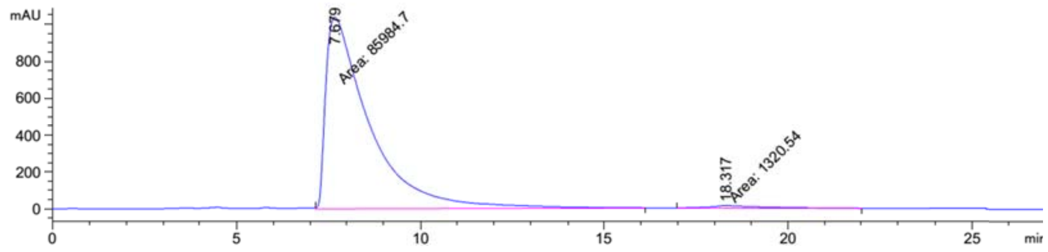

Signal 1: DAD1 A, Sig=254,4 Ref=360,100

Peak #	RetTime [min]	Type	Width [min]	Area [mAU*s]	Height [mAU]	Area %
1	7.679	MM	1.3810	8.59847e4	1037.73962	98.4874
2	18.317	MM	1.8161	1320.54102	12.11875	1.5126

Supplementary Figure 73. HPLC traces for racemic and chiral product **3e**

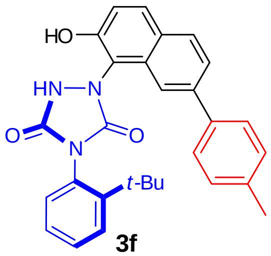

3f

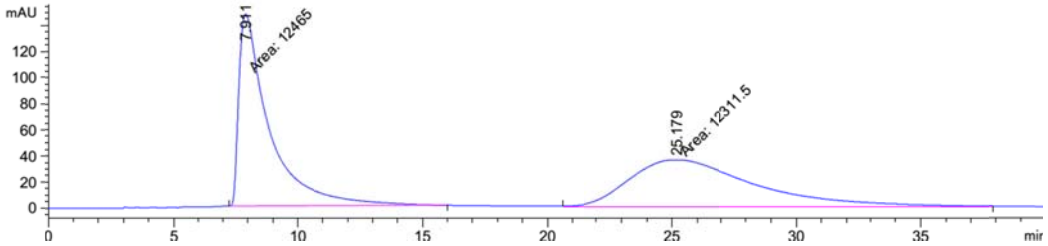

Signal 4: DAD1 F, Sig=260,4 Ref=360,100

Peak #	RetTime [min]	Type	Width [min]	Area [mAU*s]	Height [mAU]	Area %
1	7.911	MM	1.4099	1.24650e4	147.34738	50.3098
2	25.179	MM	5.7460	1.23115e4	35.71004	49.6902

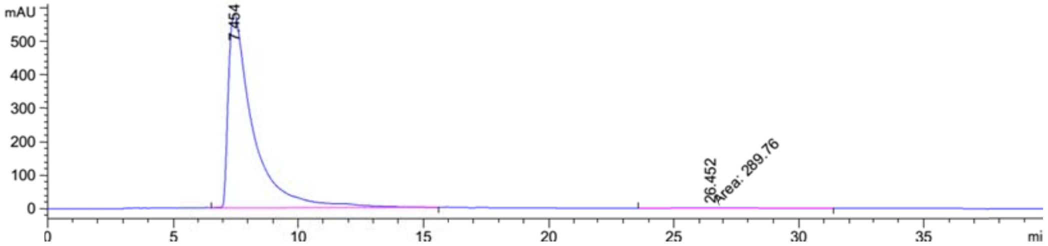

Signal 4: DAD1 F, Sig=260,4 Ref=360,100

Peak #	RetTime [min]	Type	Width [min]	Area [mAU*s]	Height [mAU]	Area %
1	7.454	BB	0.9679	3.93595e4	579.88989	99.2692
2	26.452	MM	4.4040	289.75977	1.09658	0.7308

Supplementary Figure 74. HPLC traces for racemic and chiral product **3f**

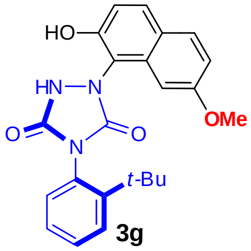

3g

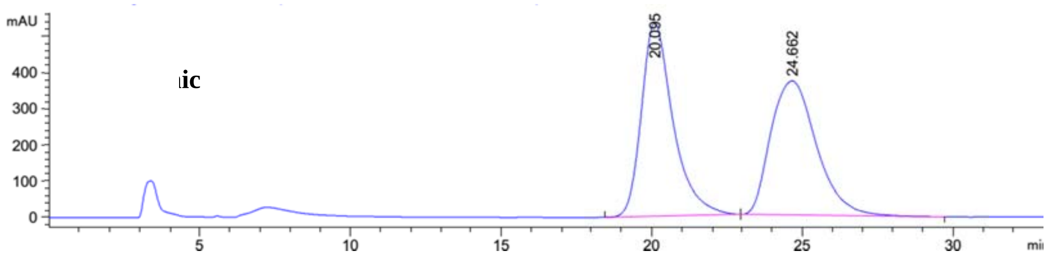

Signal 3: DAD1 D, Sig=230,4 Ref=360,100

Peak #	RetTime [min]	Type	Width [min]	Area [mAU*s]	Height [mAU]	Area %
1	20.095	BB	1.0863	3.86933e4	534.69824	49.8405
2	24.662	BB	1.6616	3.89410e4	371.29333	50.1595

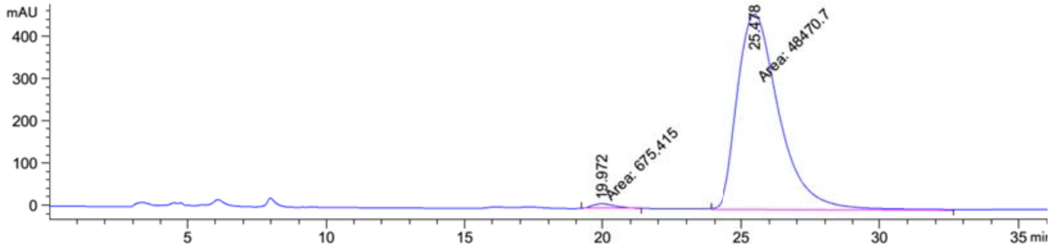

Signal 3: DAD1 D, Sig=230,4 Ref=360,100

Peak #	RetTime [min]	Type	Width [min]	Area [mAU*s]	Height [mAU]	Area %
1	19.972	MM	1.0137	675.41522	11.10443	1.3743
2	25.478	MM	1.7511	4.84707e4	461.33981	98.6257

Supplementary Figure 75. HPLC traces for racemic and chiral product **3g**

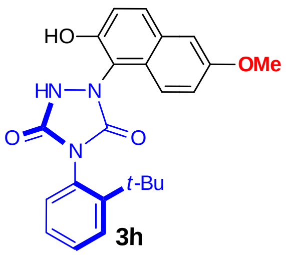

3h

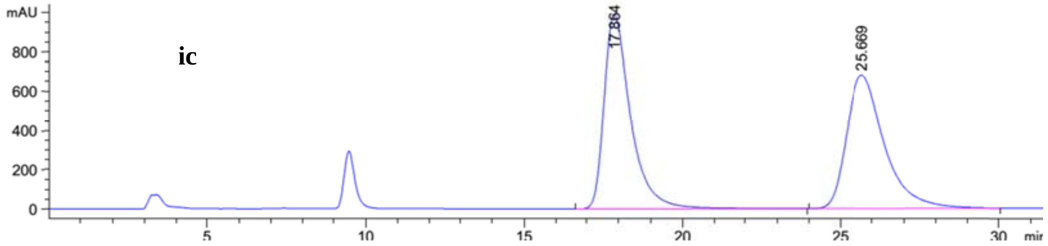

Signal 3: DAD1 D, Sig=230,4 Ref=360,100

Peak #	RetTime [min]	Type	Width [min]	Area [mAU*s]	Height [mAU]	Area %
1	17.864	BB	0.8559	5.68686e4	990.44153	50.2419
2	25.669	BB	1.2432	5.63211e4	679.34686	49.7581

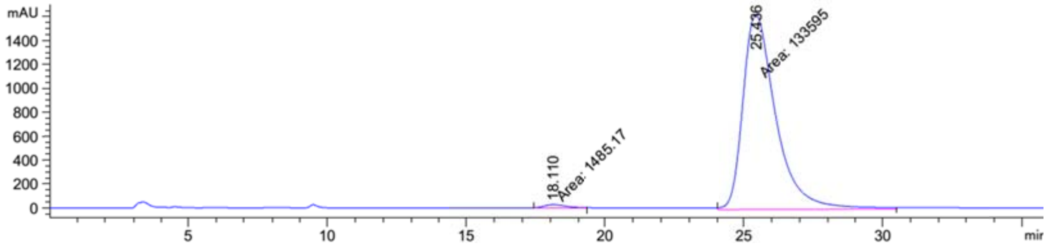

Signal 3: DAD1 D, Sig=230,4 Ref=360,100

Peak #	RetTime [min]	Type	Width [min]	Area [mAU*s]	Height [mAU]	Area %
1	18.110	MM	0.8960	1485.17236	27.62682	1.0995
2	25.436	MM	1.3662	1.33595e5	1629.82275	98.9005

Supplementary Figure 76. HPLC traces for racemic and chiral product **3h**

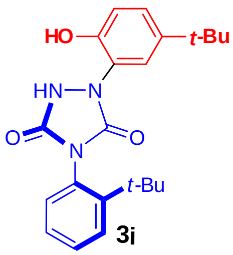

3i

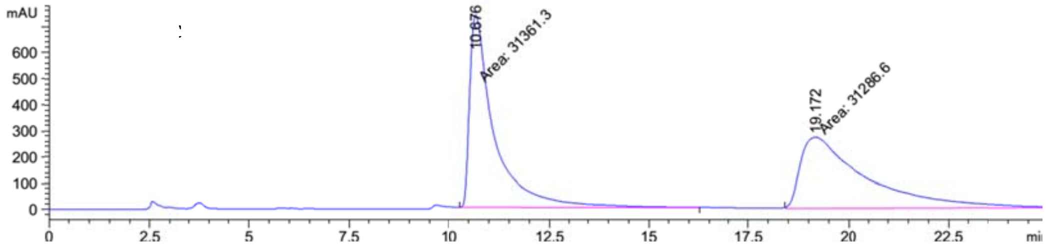

Signal 2: DAD1 B, Sig=214,4 Ref=360,100

Peak #	RetTime [min]	Type	Width [min]	Area [mAU*s]	Height [mAU]	Area %
1	10.676	MM	0.7099	3.13613e4	736.30469	50.0597
2	19.172	MM	1.9173	3.12866e4	271.96204	49.9403

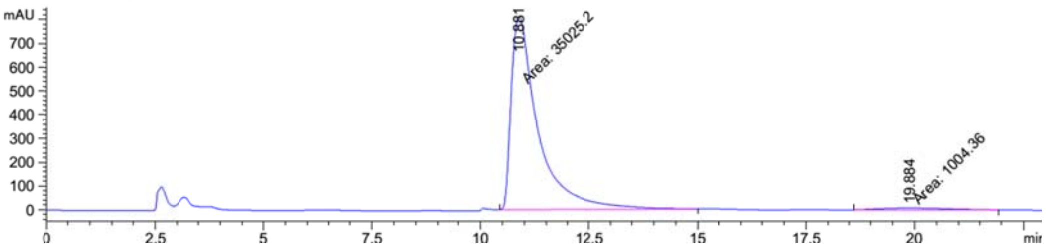

Signal 2: DAD1 B, Sig=214,4 Ref=360,100

Peak #	RetTime [min]	Type	Width [min]	Area [mAU*s]	Height [mAU]	Area %
1	10.881	MM	0.7193	3.50252e4	811.56061	97.2124
2	19.884	MM	1.8358	1004.35553	9.11805	2.7876

Supplementary Figure 77. HPLC traces for racemic and chiral product **3i**

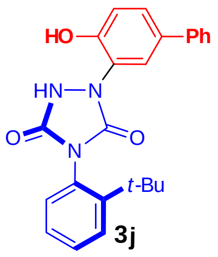

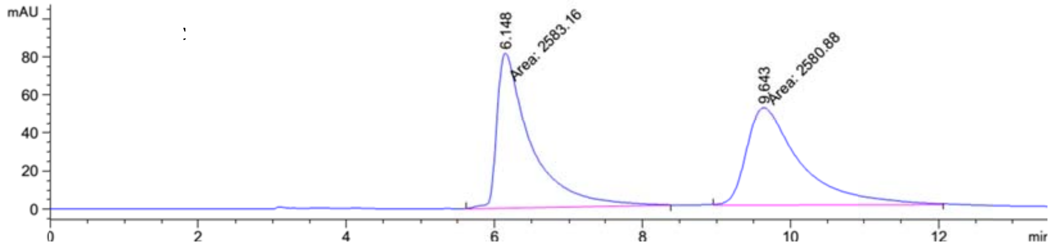

Signal 1: DAD1 A, Sig=254,4 Ref=360,100

Peak #	RetTime [min]	Type	Width [min]	Area [mAU*s]	Height [mAU]	Area %
1	6.148	MM	0.5288	2583.15820	81.42079	50.0220
2	9.643	MM	0.8381	2580.88477	51.32141	49.9780

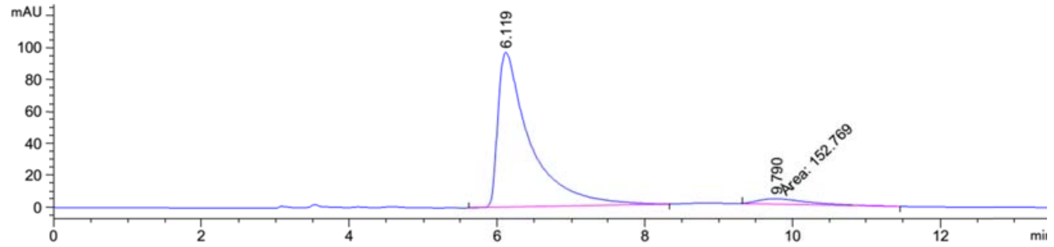

Signal 1: DAD1 A, Sig=254,4 Ref=360,100

Peak #	RetTime [min]	Type	Width [min]	Area [mAU*s]	Height [mAU]	Area %
1	6.119	BB	0.4263	2953.32275	97.25825	95.0816
2	9.790	MM	0.7732	152.76852	3.29316	4.9184

Supplementary Figure 78. HPLC traces for racemic and chiral product **3i**

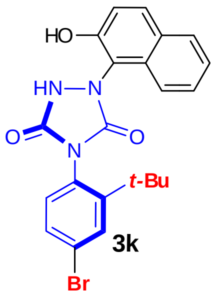

3k

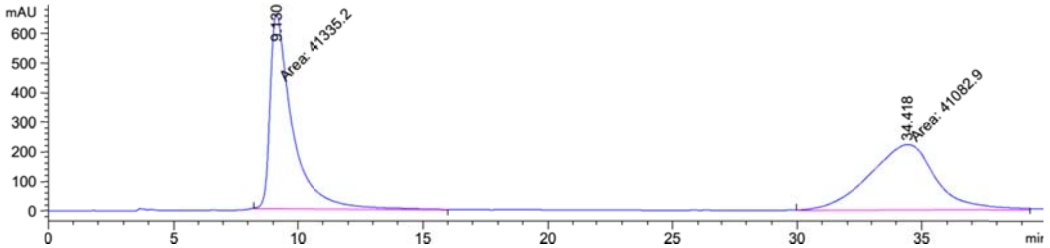

Signal 2: DAD1 B, Sig=230,4 Ref=360,100

Peak #	RetTime [min]	Type	Width [min]	Area [mAU*s]	Height [mAU]	Area %
1	9.130	MM	1.0444	4.13352e4	659.65808	50.1531
2	34.418	MM	3.0861	4.10829e4	221.87022	49.8469

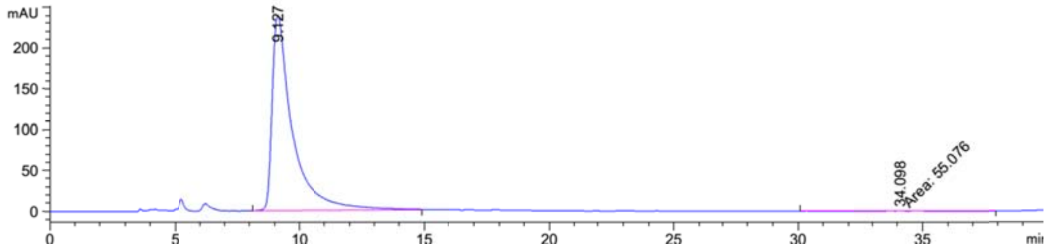

Signal 2: DAD1 B, Sig=230,4 Ref=360,100

Peak #	RetTime [min]	Type	Width [min]	Area [mAU*s]	Height [mAU]	Area %
1	9.127	BB	0.8019	1.34387e4	239.03166	99.5918
2	34.098	MM	2.0607	55.07603	4.45453e-1	0.4082

Supplementary Figure 79. HPLC traces for racemic and chiral product 3**k**

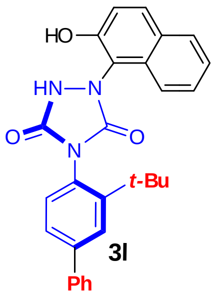

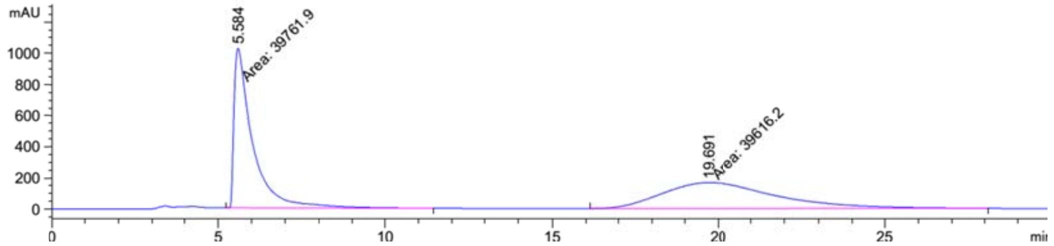

Signal 3: DAD1 D, Sig=230,4 Ref=360,100

Peak #	RetTime [min]	Type	Width [min]	Area [mAU*s]	Height [mAU]	Area %
1	5.584	MM	0.6474	3.97619e4	1023.63043	50.0918
2	19.691	MM	3.9670	3.96162e4	166.44264	49.9082

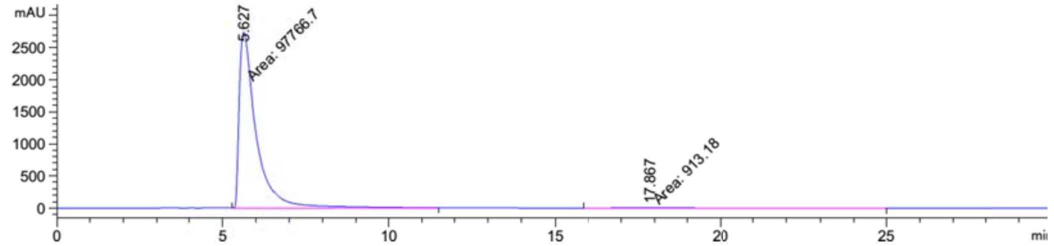

Signal 3: DAD1 D, Sig=230,4 Ref=360,100

Peak #	RetTime [min]	Type	Width [min]	Area [mAU*s]	Height [mAU]	Area %
1	5.627	MM	0.5936	9.77667e4	2745.20557	99.0746
2	17.867	MM	3.7669	913.17957	4.04036	0.9254

Supplementary Figure 80. HPLC traces for racemic and chiral product **3l**

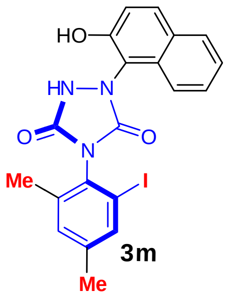

3m

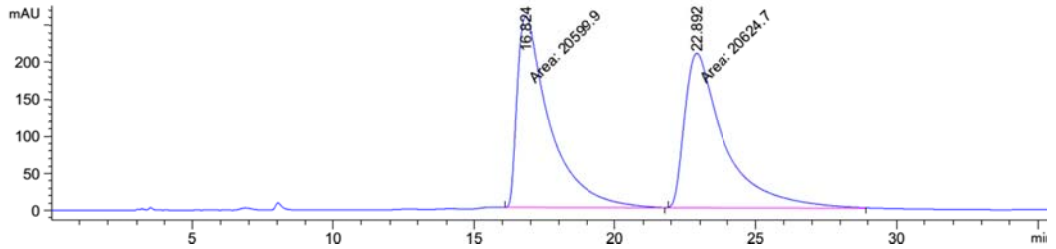

Signal 3: DAD1 D, Sig=230,4 Ref=360,100

Peak #	RetTime [min]	Type	Width [min]	Area [mAU*s]	Height [mAU]	Area %
1	16.824	MM	1.3234	2.05999e4	259.42923	49.9700
2	22.892	MM	1.6454	2.06247e4	208.91164	50.0300

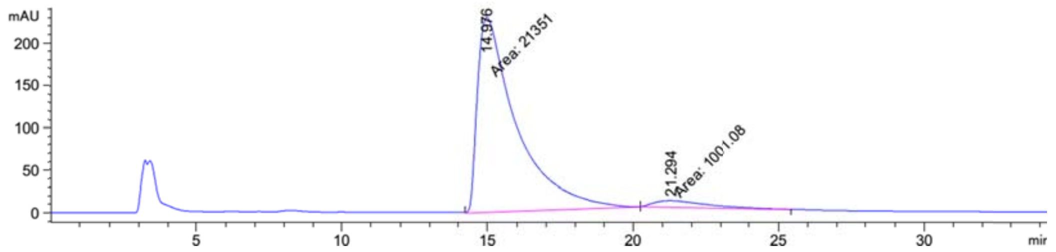

Signal 3: DAD1 D, Sig=230,4 Ref=360,100

Peak #	RetTime [min]	Type	Width [min]	Area [mAU*s]	Height [mAU]	Area %
1	14.976	MM	1.5520	2.13510e4	229.29010	95.5213
2	21.294	MM	2.1174	1001.07733	7.87972	4.4787

Supplementary Figure 81. HPLC traces for racemic and chiral product **3m**

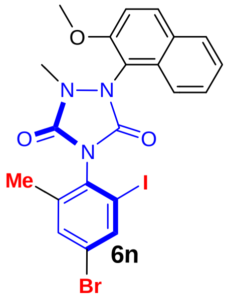

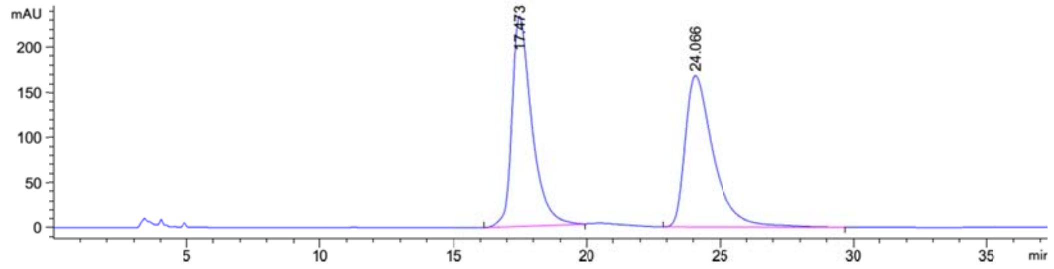

Signal 5: DAD1 E, Sig=240,4 Ref=360,100

Peak #	RetTime [min]	Type	Width [min]	Area [mAU*s]	Height [mAU]	Area %
1	17.473	BB	0.7668	1.19570e4	232.97623	49.1906
2	24.066	BB	1.0996	1.23505e4	168.00992	50.8094

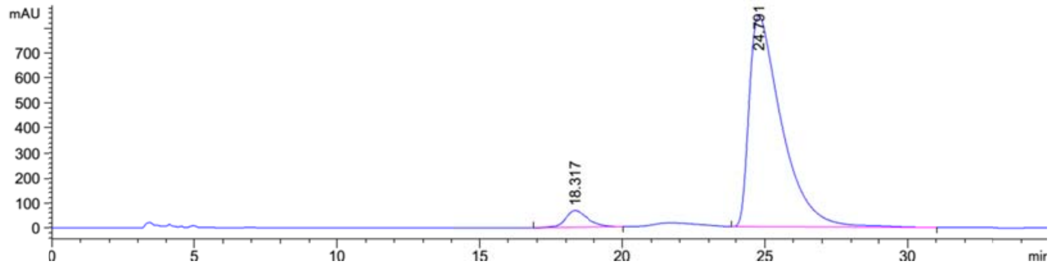

Signal 5: DAD1 E, Sig=240,4 Ref=360,100

Peak #	RetTime [min]	Type	Width [min]	Area [mAU*s]	Height [mAU]	Area %
1	18.317	BB	0.8417	3739.70654	66.74394	5.3217
2	24.791	BB	1.1689	6.65330e4	844.76141	94.6783

Supplementary Figure 82. HPLC traces for racemic and chiral product **3n**

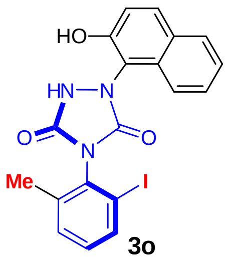

3o

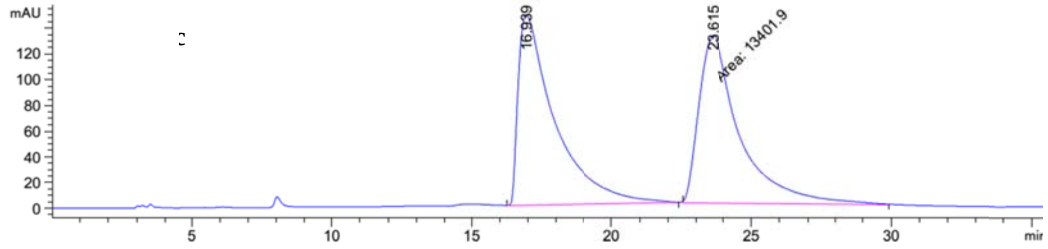

Signal 3: DAD1 D, Sig=230,4 Ref=360,100

Peak #	RetTime [min]	Type	Width [min]	Area [mAU*s]	Height [mAU]	Area %
1	16.939	BB	1.2734	1.34126e4	147.98486	50.0201
2	23.615	MM	1.7101	1.34019e4	130.61610	49.9799

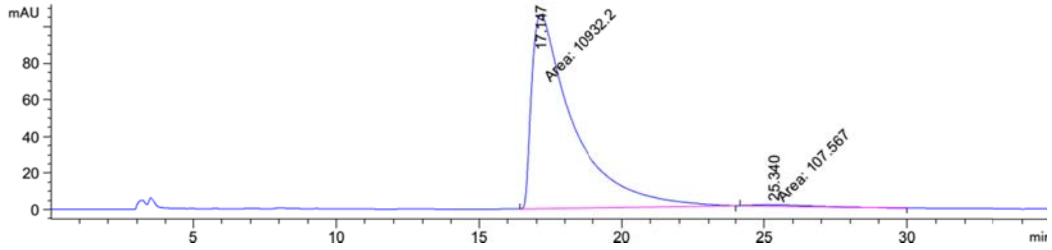

Signal 3: DAD1 D, Sig=230,4 Ref=360,100

Peak #	RetTime [min]	Type	Width [min]	Area [mAU*s]	Height [mAU]	Area %
1	17.147	MM	1.7089	1.09322e4	106.61943	99.0256
2	25.340	MM	2.2101	107.56695	8.11189e-1	0.9744

Supplementary Figure 83. HPLC traces for racemic and chiral product **3o**

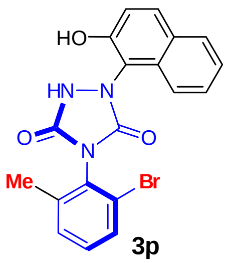

3p

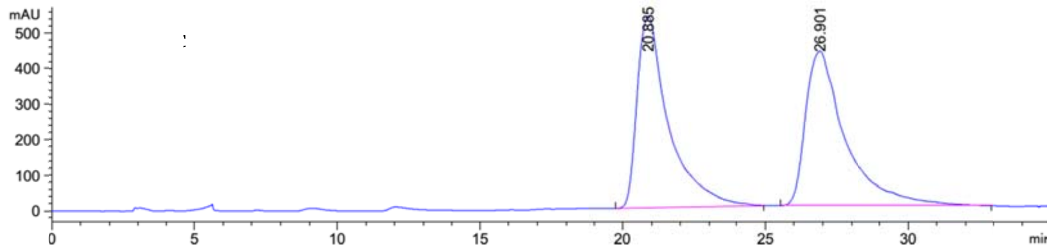

Signal 2: DAD1 B, Sig=230,4 Ref=360,100

Peak #	RetTime [min]	Type	Width [min]	Area [mAU*s]	Height [mAU]	Area %
1	20.885	BB	1.1082	4.10632e4	538.15417	49.3005
2	26.901	BB	1.3654	4.22285e4	433.90631	50.6995

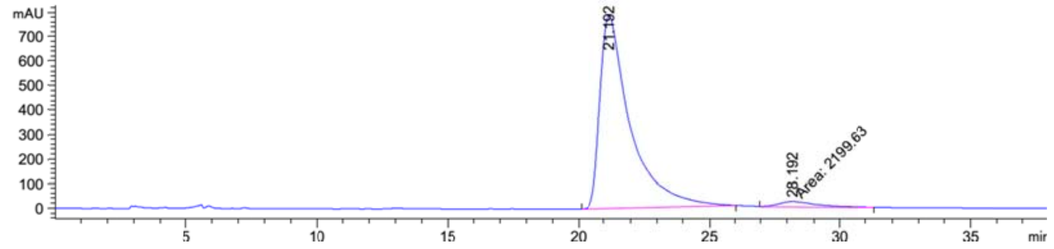

Signal 2: DAD1 B, Sig=230,4 Ref=360,100

Peak #	RetTime [min]	Type	Width [min]	Area [mAU*s]	Height [mAU]	Area %
1	21.192	BB	1.1455	6.27380e4	789.14203	96.6127
2	28.192	MM	1.6214	2199.63159	22.61013	3.3873

Supplementary Figure 84. HPLC traces for racemic and chiral product **3p**

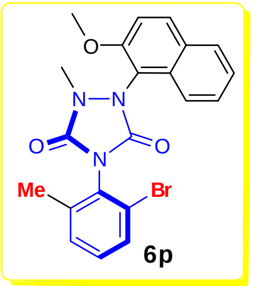

6p

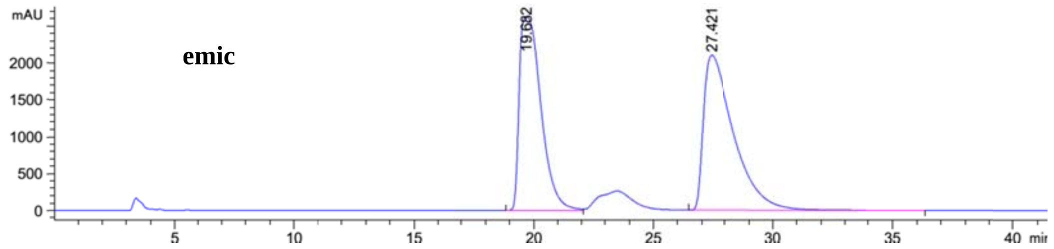

Signal 3: DAD1 D, Sig=230,4 Ref=360,100

Peak #	RetTime [min]	Type	Width [min]	Area [mAU*s]	Height [mAU]	Area %
1	19.683	BB	0.7626	5284.08643	102.98508	49.5047
2	27.420	BB	1.1965	5389.81543	62.72319	50.4953

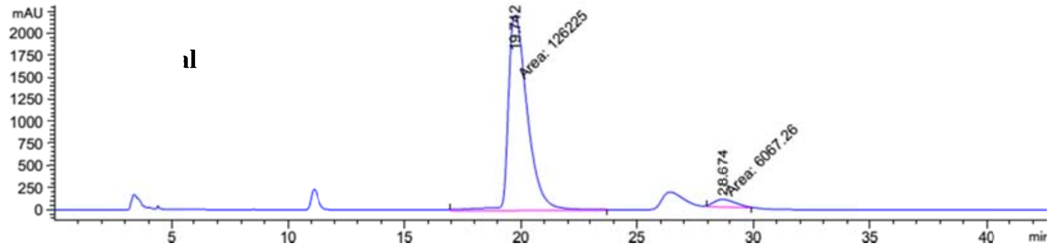

Signal 3: DAD1 D, Sig=230,4 Ref=360,100

Peak #	RetTime [min]	Type	Width [min]	Area [mAU*s]	Height [mAU]	Area %
1	19.742	MM	0.9511	1.26225e5	2211.98633	95.4137
2	28.674	MM	1.1229	6067.25830	90.05204	4.5863

Supplementary Figure 85. HPLC traces for racemic and chiral product **6p (from 3p)**

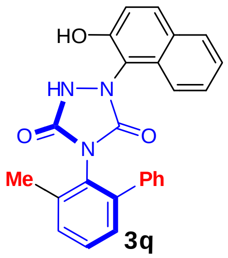

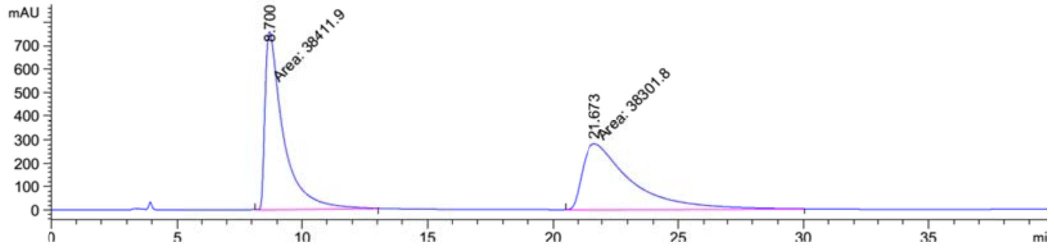

Signal 3: DAD1 D, Sig=230,4 Ref=360,100

Peak #	RetTime [min]	Type	Width [min]	Area [mAU*s]	Height [mAU]	Area %
1	8.700	MM	0.8440	3.84119e4	758.56006	50.0718
2	21.673	MM	2.2634	3.83018e4	282.03787	49.9282

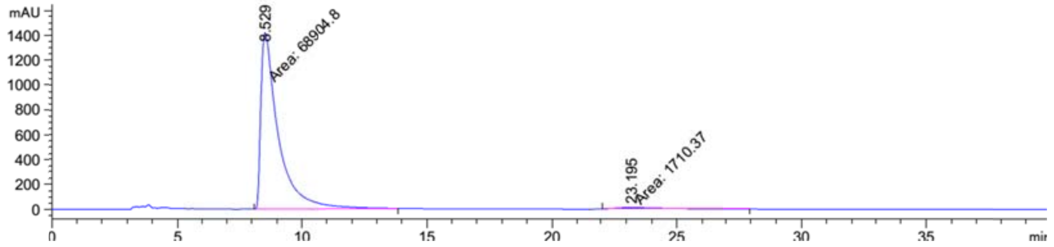

Signal 3: DAD1 D, Sig=230,4 Ref=360,100

Peak #	RetTime [min]	Type	Width [min]	Area [mAU*s]	Height [mAU]	Area %
1	8.529	MM	0.8094	6.89048e4	1418.85254	97.5779
2	23.195	MM	2.4139	1710.36536	11.80903	2.4221

Supplementary Figure 86. HPLC traces for racemic and chiral product **3q**

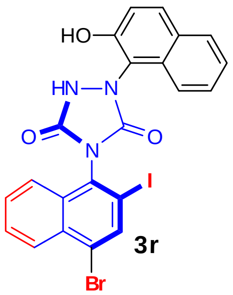

3r

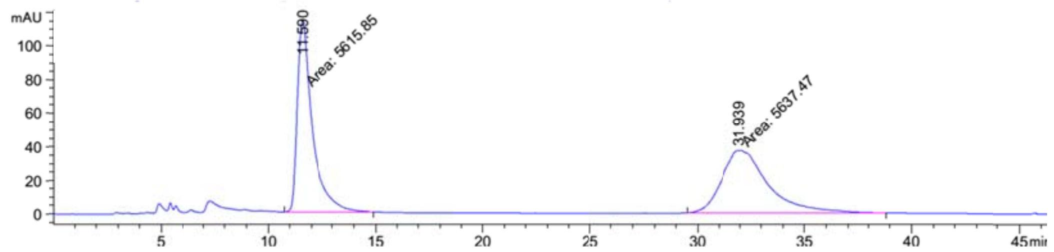

Signal 1: DAD1 A, Sig=254,4 Ref=360,100

Peak #	RetTime [min]	Type	Width [min]	Area [mAU*s]	Height [mAU]	Area %
1	11.590	MM	0.8171	5615.85498	114.55194	49.9040
2	31.939	MM	2.5212	5637.47217	37.26781	50.0960

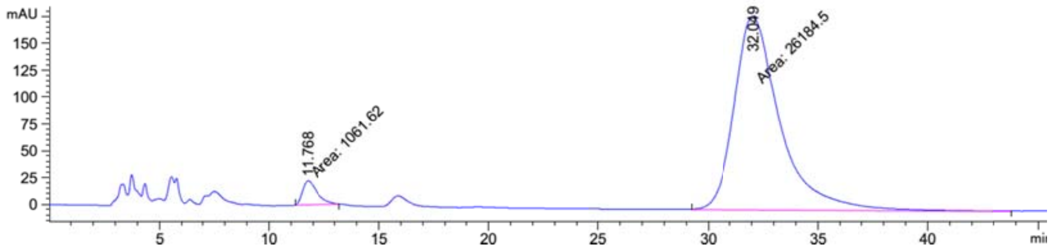

Signal 1: DAD1 A, Sig=254,4 Ref=360,100

Peak #	RetTime [min]	Type	Width [min]	Area [mAU*s]	Height [mAU]	Area %
1	11.768	MM	0.7849	1061.61841	22.54213	3.8964
2	32.049	MM	2.4113	2.61845e4	180.98318	96.1036

Supplementary Figure 87. HPLC traces for racemic and chiral product **3r**

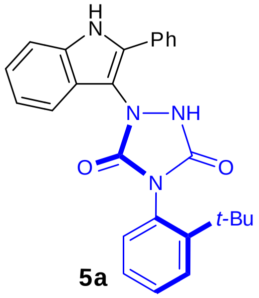

5a

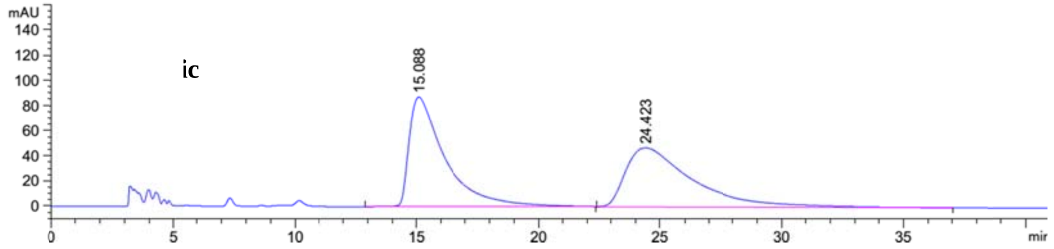

Signal 3: DAD1 D, Sig=230,4 Ref=360,100

Peak #	[illegible] [min]	Type	Width [min]	Area [mAU*s]	Height [mAU]	Area %
1	15.088	BB	1.4570	8977.34961	87.35474	50.3281
2	24.423	BB	2.2962	8860.29297	46.72713	49.6719

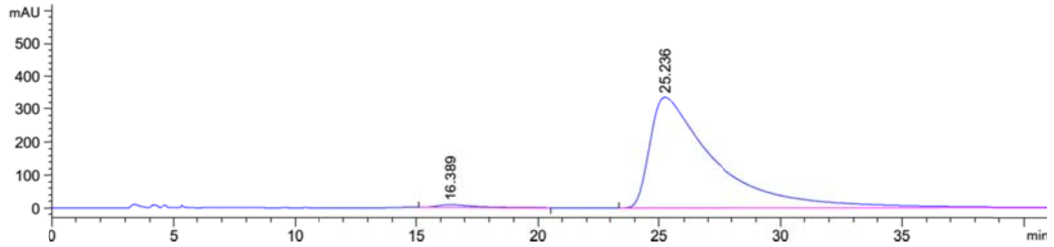

Signal 3: DAD1 D, Sig=230,4 Ref=360,100

Peak #	RetTime [min]	Type	Width [min]	Area [mAU*s]	Height [mAU]	Area %
1	16.389	BB	1.2038	784.34259	7.69092	1.2306
2	25.236	BB	2.5384	6.29546e4	335.92117	98.7694

Supplementary Figure 88. HPLC traces for racemic and chiral product **5a**

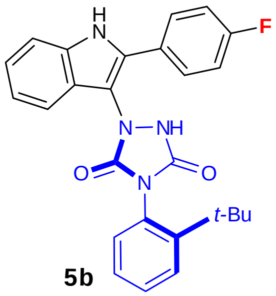

5b

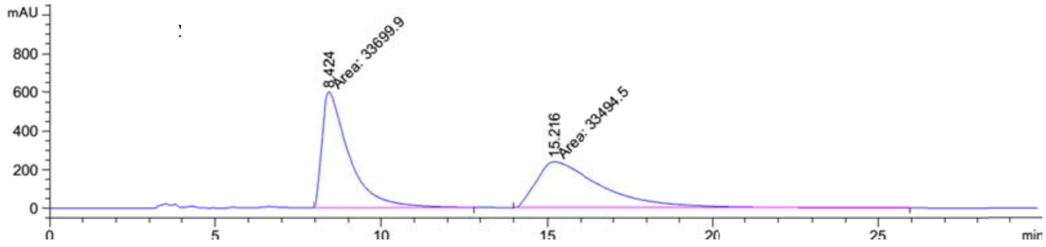

Signal 3: DAD1 D, Sig=230,4 Ref=360,100

Peak #	RetTime [min]	Type	Width [min]	Area [mAU*s]	Height [mAU]	Area %
1	8.424	MM	0.9360	3.36999e4	600.06152	50.1528
2	15.216	MM	2.3469	3.34945e4	237.86243	49.8472

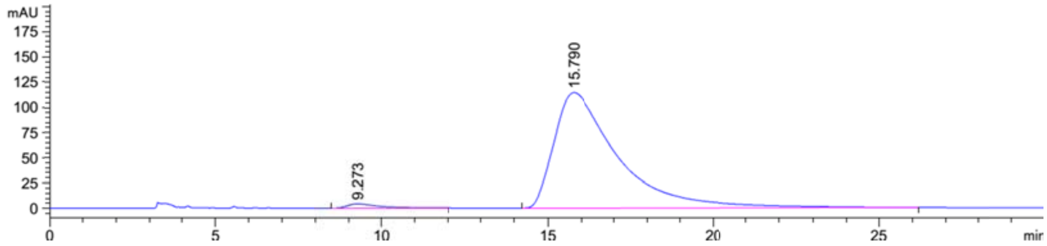

Signal 3: DAD1 D, Sig=230,4 Ref=360,100

Peak #	RetTime [min]	Type	Width [min]	Area [mAU*s]	Height [mAU]	Area %
1	9.273	BB	0.7866	285.08072	4.39594	1.8122
2	15.790	BB	1.8827	1.54458e4	114.86951	98.1878

Supplementary Figure 89. HPLC traces for racemic and chiral product **5b**

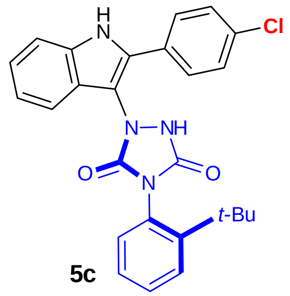

5c

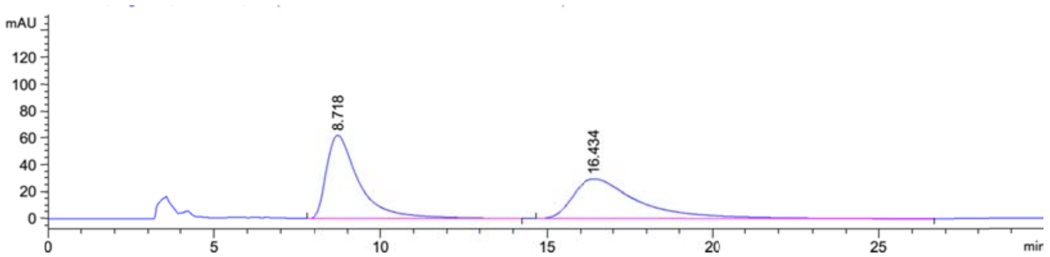

Signal 4: DAD1 E, Sig=240,4 Ref=360,100

Peak #	RetTime [min]	Type	Width [min]	Area [mAU*s]	Height [mAU]	Area %
1	8.718	BB	0.9944	4141.77441	61.44489	50.9331
2	16.434	BB	1.8434	3990.02124	29.06867	49.0669

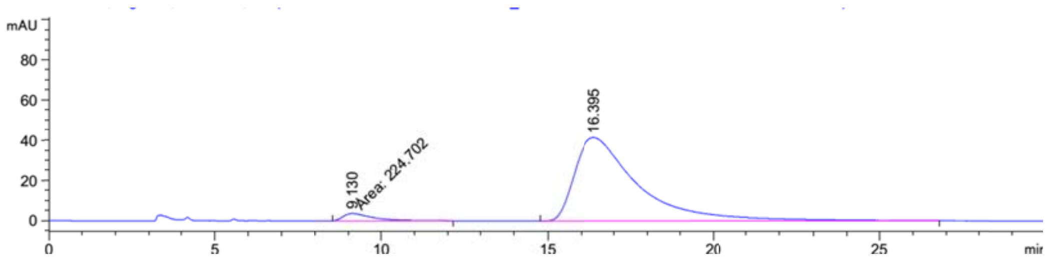

Signal 4: DAD1 E, Sig=240,4 Ref=360,100

Peak #	RetTime [min]	Type	Width [min]	Area [mAU*s]	Height [mAU]	Area %
1	9.130	MM	1.0208	224.70242	3.66856	3.9330
2	16.395	BB	1.8487	5488.61377	41.34760	96.0670

Supplementary Figure 90. HPLC traces for racemic and chiral product **5c**

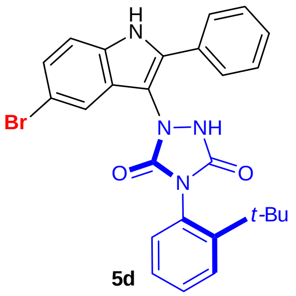

5d

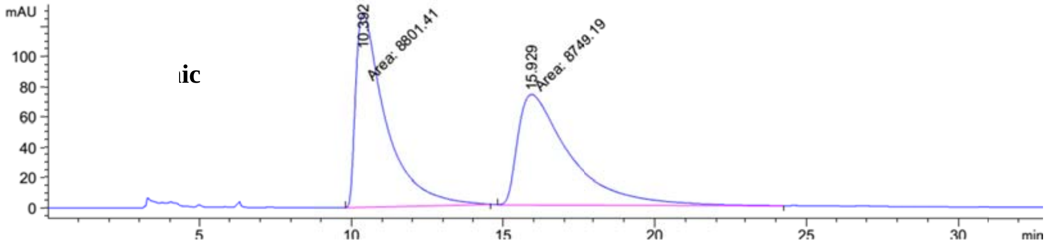

Signal 1: DAD1 A, Sig=254,4 Ref=360,100

Peak #	RetTime [min]	Type	Width [min]	Area [mAU*s]	Height [mAU]	Area %
1	10.392	MM	1.1467	8801.41016	127.92109	50.1488
2	15.929	MM	1.9968	8749.18555	73.02751	49.8512

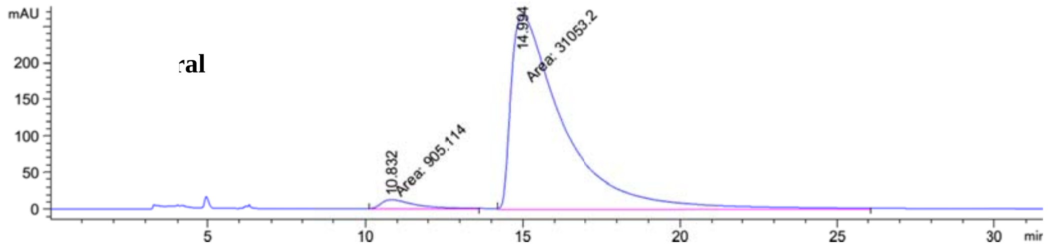

Signal 1: DAD1 A, Sig=254,4 Ref=360,100

Peak #	RetTime [min]	Type	Width [min]	Area [mAU*s]	Height [mAU]	Area %
1	10.832	MM	1.1988	905.11371	12.58342	2.8322
2	14.994	MM	1.9483	3.10532e4	265.64777	97.1678

Supplementary Figure 91. HPLC traces for racemic and chiral product **5d**

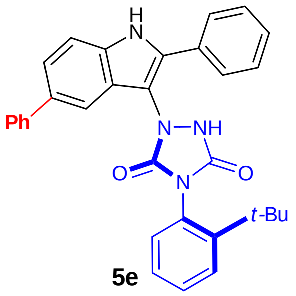

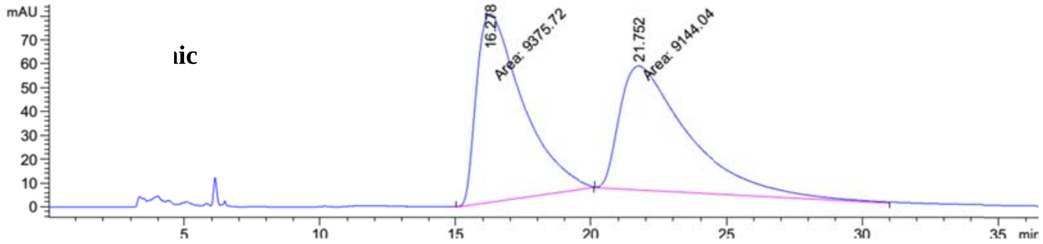

Signal 1: DAD1 A, Sig=254,4 Ref=360,100

Peak #	RetTime [min]	Type	Width [min]	Area [mAU*s]	Height [mAU]	Area %
1	16.278	MM	1.9777	9375.71777	79.00997	50.6255
2	21.752	MM	2.9255	9144.03516	52.09471	49.3745

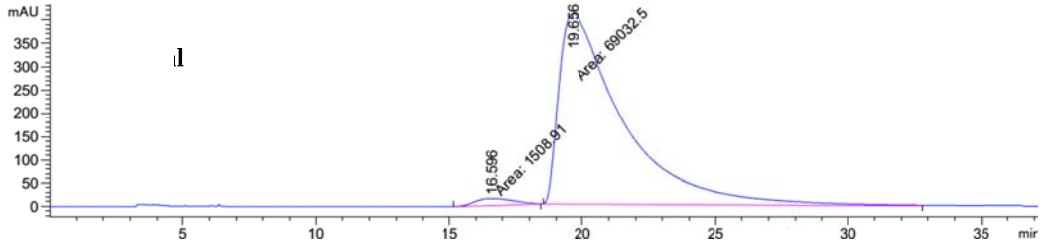

Signal 1: DAD1 A, Sig=254,4 Ref=360,100

Peak #	RetTime [min]	Type	Width [min]	Area [mAU*s]	Height [mAU]	Area %
1	16.596	MM	1.6612	1508.90515	15.13911	2.1390
2	19.656	MM	2.8164	6.90325e4	408.51556	97.8610

Supplementary Figure 92. HPLC traces for racemic and chiral product **5e**

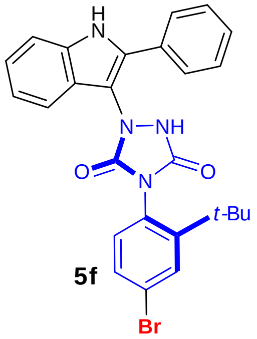

5f

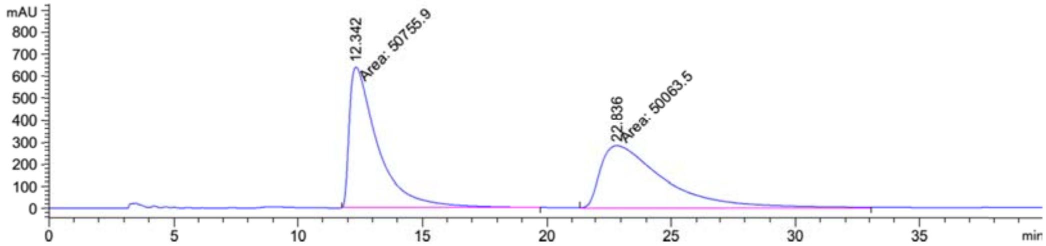

Signal 3: DAD1 D, Sig=230,4 Ref=360,100

Peak #	RetTime [min]	Type	Width [min]	Area [mAU*s]	Height [mAU]	Area %
1	12.342	MM	1.3209	5.07559e4	640.40491	50.3434
2	22.836	MM	2.9573	5.00635e4	282.14417	49.6566

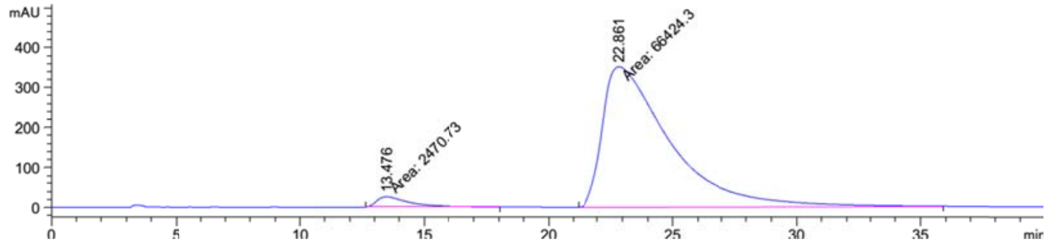

Signal 3: DAD1 D, Sig=230,4 Ref=360,100

Peak #	RetTime [min]	Type	Width [min]	Area [mAU*s]	Height [mAU]	Area %
1	13.476	MM	1.5620	2470.73413	26.36217	3.5862
2	22.861	MM	3.1452	6.64243e4	351.98660	96.4138

Supplementary Figure 93. HPLC traces for racemic and chiral product **5f**

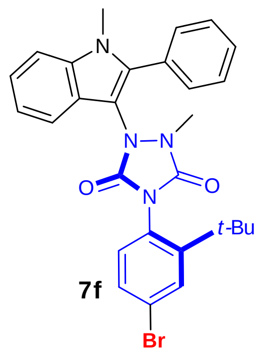

7f

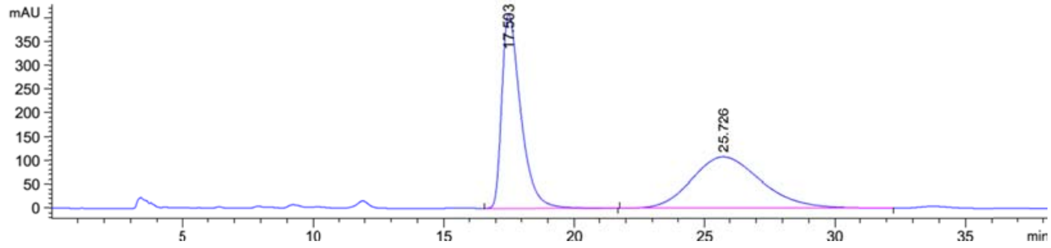

Signal 4: DAD1 D, Sig=230,4 Ref=360,100

Peak #	RetTime [min]	Type	Width [min]	Area [mAU*s]	Height [mAU]	Area %
1	17.503	BB	0.7586	2.03983e4	408.48056	50.3247
2	25.726	BB	2.6591	2.01351e4	107.30966	49.6753

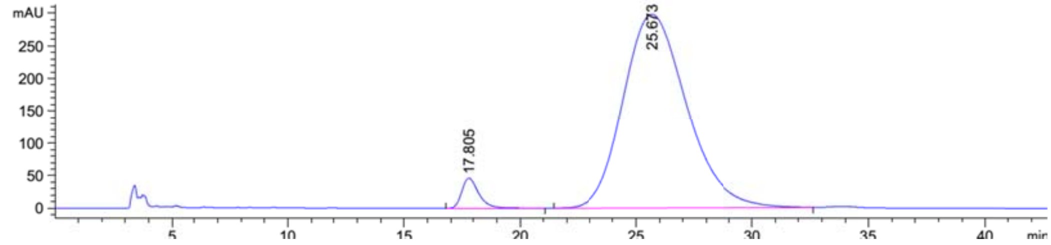

Signal 4: DAD1 D, Sig=230,4 Ref=360,100

Peak #	RetTime [min]	Type	Width [min]	Area [mAU*s]	Height [mAU]	Area %
1	17.805	MM	0.7960	2182.10669	45.68803	3.6734
2	25.673	MM	3.1833	5.72212e4	299.59048	96.3266

Supplementary Figure 94. HPLC traces for racemic and chiral product **7f (from 5f)**

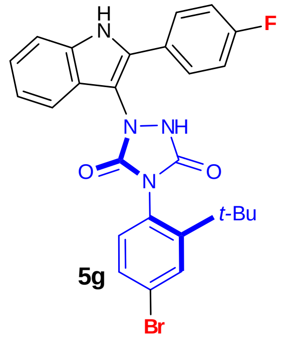

5g

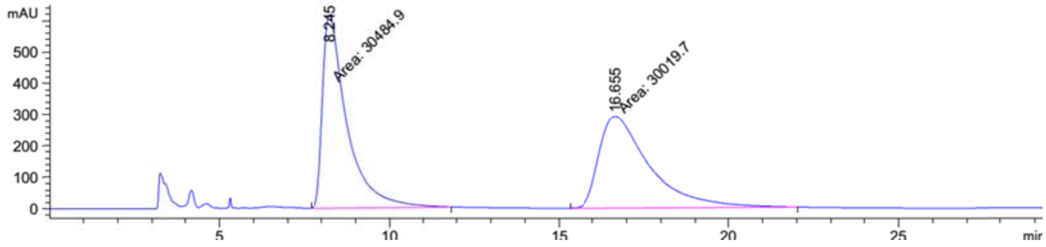

Signal 3: DAD1 D, Sig=230,4 Ref=360,100

Peak #	RetTime [min]	Type	Width [min]	Area [mAU*s]	Height [mAU]	Area %
1	8.245	MM	0.8247	3.04849e4	616.04761	50.3844
2	16.655	MM	1.7141	3.00197e4	291.88635	49.6156

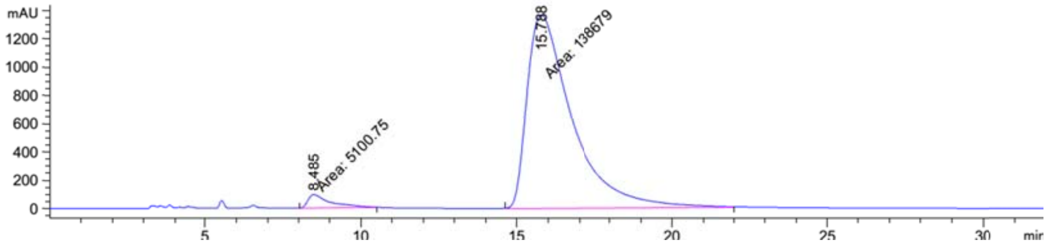

Signal 3: DAD1 D, Sig=230,4 Ref=360,100

Peak #	RetTime [min]	Type	Width [min]	Area [mAU*s]	Height [mAU]	Area %
1	8.485	MM	0.8728	5100.74609	97.39775	3.5476
2	15.788	MM	1.6902	1.38679e5	1367.44885	96.4524

Supplementary Figure 95. HPLC traces for racemic and chiral product **5g**

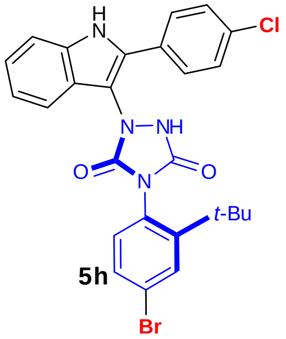

5h

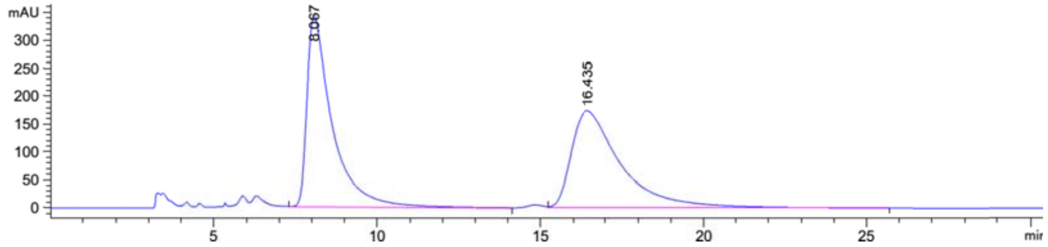

Signal 3: DAD1 D, Sig=230,4 Ref=360,100

Peak #	RetTime [min]	Type	Width [min]	Area [mAU*s]	Height [mAU]	Area %
1	8.067	BB	0.7836	1.86869e4	344.08249	50.0617
2	16.435	VB	1.5657	1.86408e4	174.02386	49.9383

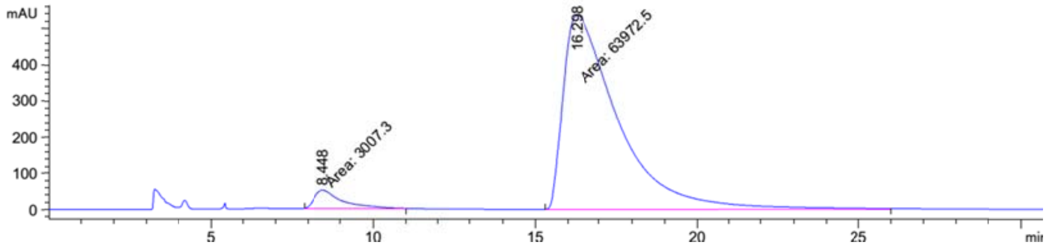

Signal 3: DAD1 D, Sig=230,4 Ref=360,100

Peak #	RetTime [min]	Type	Width [min]	Area [mAU*s]	Height [mAU]	Area %
1	8.448	MM	0.9887	3007.29541	50.69624	4.4899
2	16.298	MM	1.9885	6.39725e4	536.18127	95.5101

Supplementary Figure 96. HPLC traces for racemic and chiral product **5h**

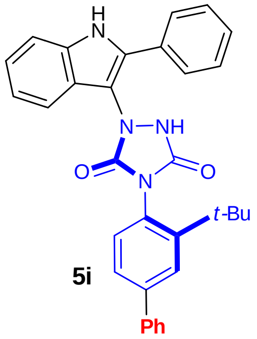

5i

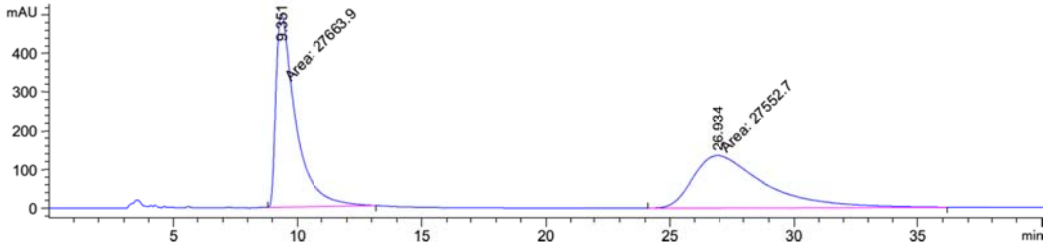

Signal 3: DAD1 D, Sig=230,4 Ref=360,100

Peak #	RetTime [min]	Type	Width [min]	Area [mAU*s]	Height [mAU]	Area %
1	9.351	MM	0.9215	2.76639e4	500.32846	50.1008
2	26.934	MM	3.3790	2.75527e4	135.90034	49.8992

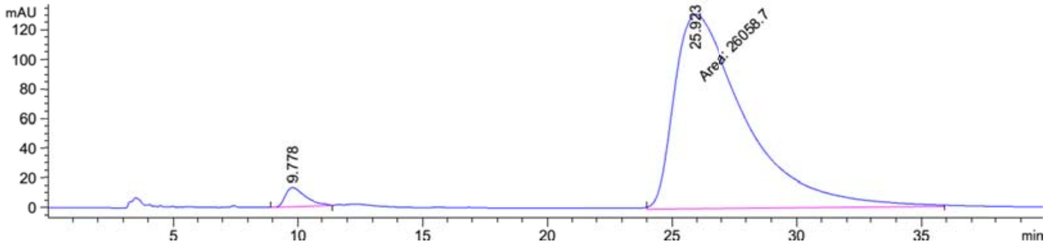

Signal 3: DAD1 D, Sig=230,4 Ref=360,100

Peak #	RetTime [min]	Type	Width [min]	Area [mAU*s]	Height [mAU]	Area %
1	9.778	BB	0.8102	735.93195	13.00519	2.7466
2	25.923	MM	3.3048	2.60587e4	131.41745	97.2534

Supplementary Figure 97. HPLC traces for racemic and chiral product **5i**

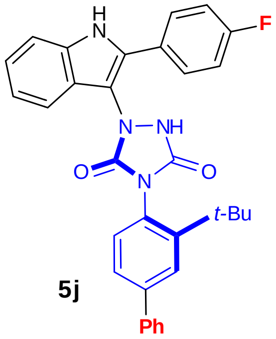

5j

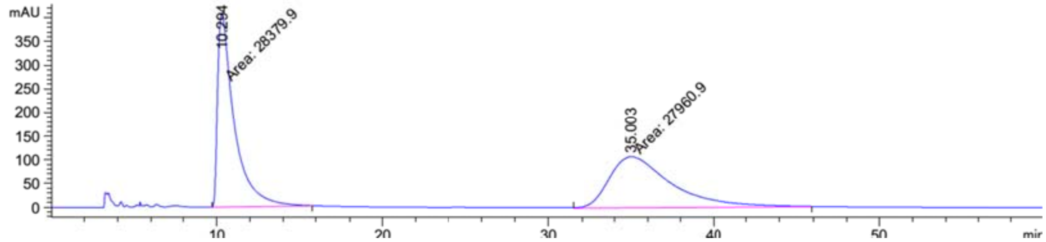

Signal 3: DAD1 D, Sig=230,4 Ref=360,100

Peak #	RetTime [min]	Type	Width [min]	Area [mAU*s]	Height [mAU]	Area %
1	10.294	MM	1.1623	2.83799e4	406.96420	50.3719
2	35.003	MM	4.3351	2.79609e4	107.49846	49.6281

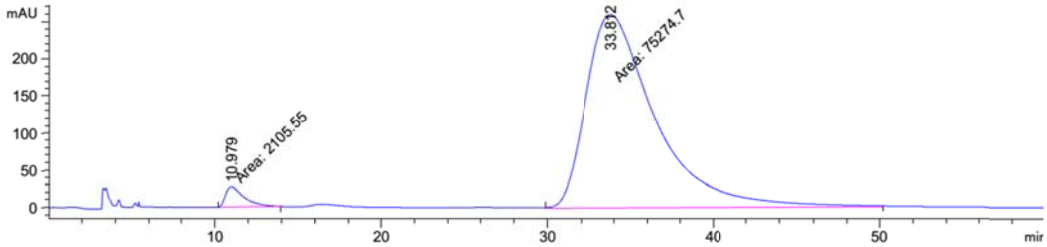

Signal 3: DAD1 D, Sig=230,4 Ref=360,100

Peak #	RetTime [min]	Type	Width [min]	Area [mAU*s]	Height [mAU]	Area %
1	10.979	MM	1.2801	2105.54785	27.41308	2.7210
2	33.812	MM	4.8308	7.52747e4	259.70331	97.2790

Supplementary Figure 98. HPLC traces for racemic and chiral product **5j**

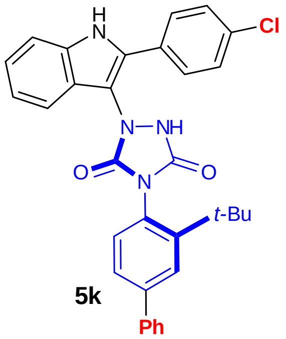

5k

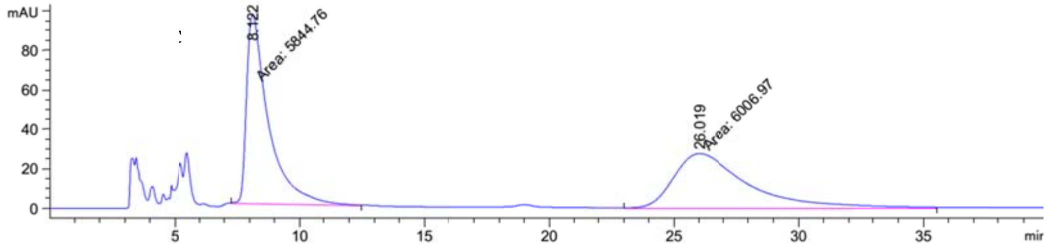

Signal 3: DAD1 D, Sig=230,4 Ref=360,100

Peak #	RetTime [min]	Type	Width [min]	Area [mAU*s]	Height [mAU]	Area %
1	8.122	MM	1.0137	5844.76318	96.09410	49.3157
2	26.019	MM	3.5822	6006.96924	27.94824	50.6843

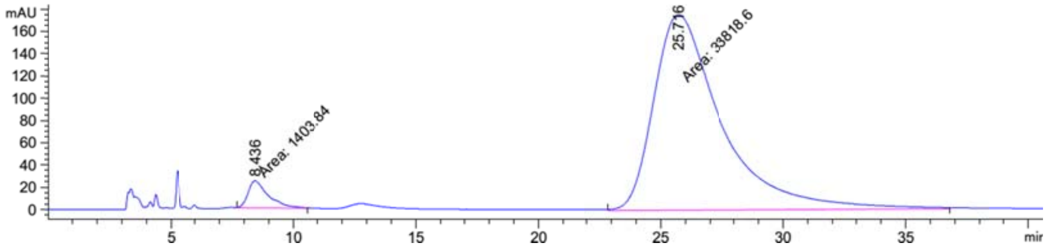

Signal 3: DAD1 D, Sig=230,4 Ref=360,100

Peak #	RetTime [min]	Type	Width [min]	Area [mAU*s]	Height [mAU]	Area %
1	8.436	MM	0.9601	1403.83508	24.36841	3.9856
2	25.716	MM	3.2141	3.38186e4	175.36803	96.0144

Supplementary Figure 99. HPLC traces for racemic and chiral product **5k**

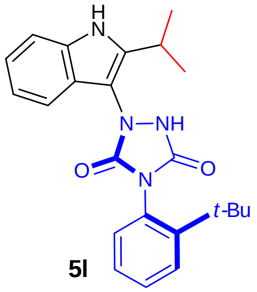

5l

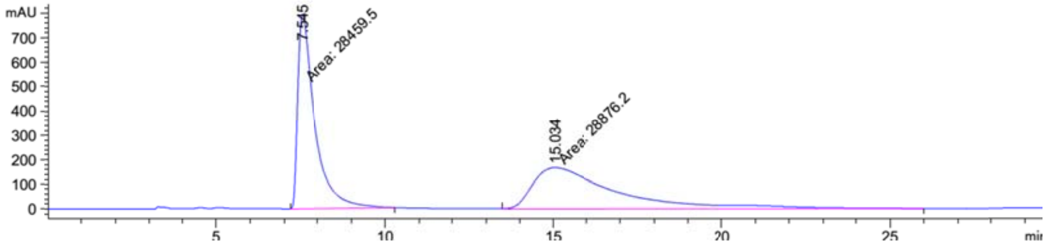

Signal 3: DAD1 D, Sig=230,4 Ref=360,100

Peak #	RetTime [min]	Type	Width [min]	Area [mAU*s]	Height [mAU]	Area %
1	7.545	MM	0.5957	2.84595e4	796.21375	49.6367
2	15.034	MM	2.8738	2.88762e4	167.46518	50.3633

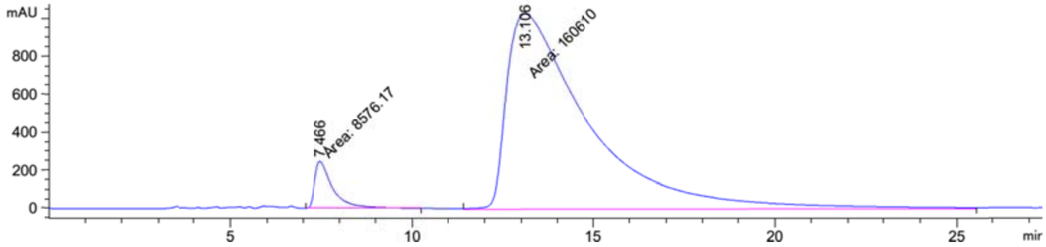

Signal 3: DAD1 D, Sig=230,4 Ref=360,100

Peak #	RetTime [min]	Type	Width [min]	Area [mAU*s]	Height [mAU]	Area %
1	7.466	MM	0.5846	8576.17383	244.49971	5.0691
2	13.106	MM	2.6139	1.60610e5	1024.05859	94.9309

Supplementary Figure 100. HPLC traces for racemic and chiral product **5l**

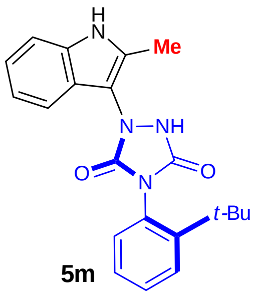

5m

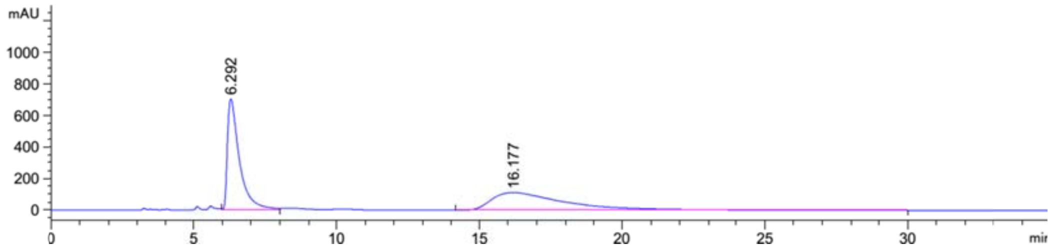

Signal 3: DAD1 D, Sig=230,4 Ref=360,100

Peak #	RetTime [min]	Type	Width [min]	Area [mAU*s]	Height [mAU]	Area %
1	6.292	VV	0.4262	2.03718e4	702.65320	49.8012
2	16.177	BB	2.5069	2.05344e4	111.69337	50.1988

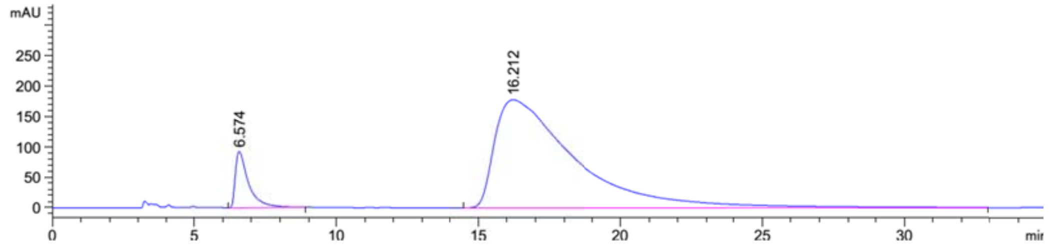

Signal 3: DAD1 D, Sig=230,4 Ref=360,100

Peak #	RetTime [min]	Type	Width [min]	Area [mAU*s]	Height [mAU]	Area %
1	6.574	BB	0.4670	2985.04956	92.86629	7.8843
2	16.212	BB	2.6169	3.48758e4	178.07568	92.1157

Supplementary Figure 101. HPLC traces for racemic and chiral product **5m**

Supplementary Table 1

Optimization of the asymmetric tyrosine click like reaction involving catalyst C6 and C7 as catalysts.[a]

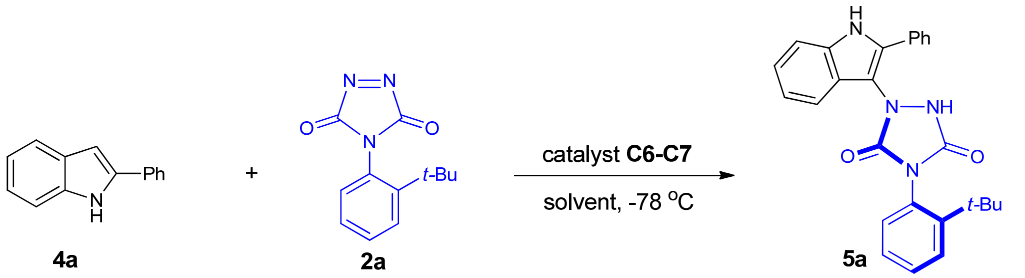

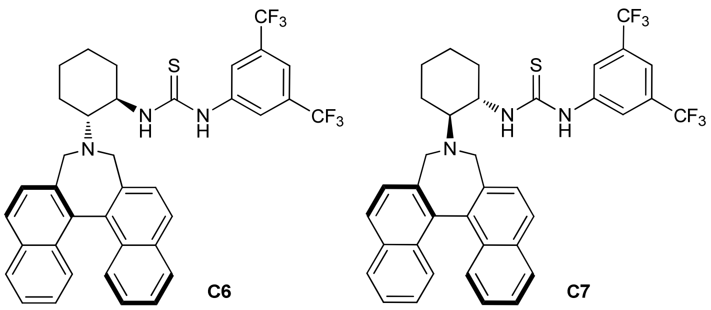

entry	solvent	catalyst (10 mol%)	time	Yield (%)[b]	ee (%)[c]
1	DCM	**C7**	< 5 min	78	-19
2	Et_2O	**C7**	24 h	74	-15
3	DCM /Et_2O=1/1	**C7**	30 min	78	-71
4	DCM	**C6**	< 5 min	76	3
5	Et_2O	**C6**	48 h	69	5
6	DCM /Et_2O=1/1	**C6**	30 min	73	5

[a] Reactions were performed with **2a** (0.12 mmol), **4a** (0.10 mmol), and catalyst (10 mol%) in 2.0 mL solvent. [b] Isolated yield. [c] Determined by HPLC analysis on a chiral stationary phase.

Supplementary Table 2—Application of asymmetric catalysis

$Sc(OTf)_3$ and Ligand (3a) effects for catalytic asymmetric synthesis of substituted 3-Hydroxy-2-Oxindole.[1]

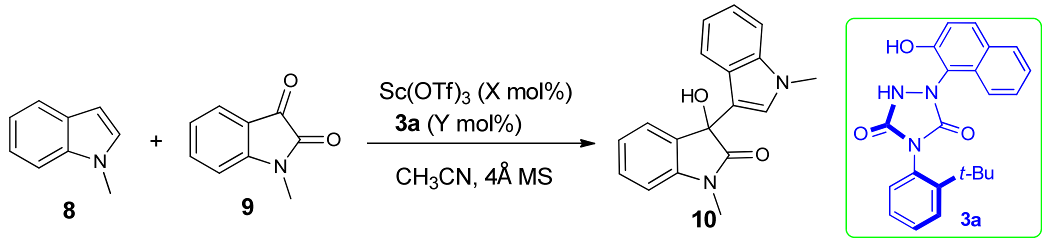

entry	$Sc(OTf)_3$ (X mol%)	**3a** (Y mol%)	T (°C)	time (h)	yield (%)[b]	ee (%)[c]
1	0	12	25	8	26	0
2	10	12	25	2	65	36
3	10	24	5	8	93	59
**4**	**10**	**12**	**5**	**8**	**96**	**62**

[a] Reactions were performed with **9** (0.1 mmol), **8** (0.3 mmol) in 1.0 mL CH_3CN under argon in the presence of 4Å molecular sieves. [b] Determined by ^{1}HNMR analysis using CH_2Br_2 as an internal standard. [c] Determined by HPLC analysis on a chiral stationary phase.

A solution of **3a** (0.012 mmol) and $Sc(OTf)_3$ (0.01 mmol) in dry MeCN (0.1 mL) was stirred at 25 °C for 30 min in an oven-dried and Ar-purged 10 mL flask equipped with a stirring bar and active 4Å MS (100 mg/0.1 mmol). The *N*-Methylisatin **9** (0.1 mmol) was added to the solution, then *N*-methylindole **8** (3.0 equiv., 0.3 mmol) was added as a solution in MeCN (0.1 mL) at the temperature indicated. After completion of reaction (monitored by TLC), the reaction mixture was directly loaded onto flash silica gel column to yield the pure product **10** in 96% yield with 62% ee.
HPLC analysis [Daicel CHIRALPAK AD-H column (250 mm × 4.6mm), hexane/i-PrO H = 70/30, 1.0 mL/min, T = 25 °C, λ = 230nm, t_R(major) = 10.0 min, t_R(minor) = 13.9 min];
^{1}H NMR (400 MHz, $CDCl_3$) δ 7.64 (d, *J* = 8.0 Hz, 1H), 7.49 (d, *J* = 7.6 Hz, 1H), 7.34 (t, *J* = 7.6 Hz, 1H), 7.24 (s, 1H), 7.19 (t, *J* = 7.2 Hz, 1H), 7.06 (t, *J* = 7.6 Hz, 2H), 6.95 (s, 1H), 6.89 (d, *J* = 8.0 Hz, 1H), 3.67 (s, 3H), 3.23 (s, 3H);
^{13}C NMR (100 MHz, $CDCl_3$) δ177.3, 143.2, 137.8, 131.2, 129.8, 127.7, 125.4, 124.9, 123.3, 122.1, 120.8, 119.8, 113.8, 109.6, 108.6, 75.6, 32.8, 26.5.

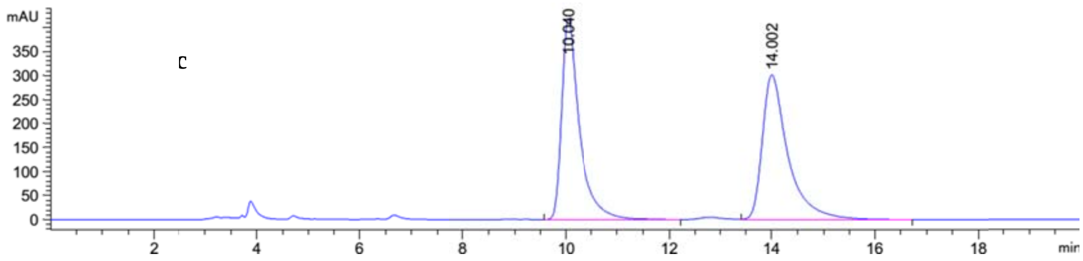

Signal 2: DAD1 B, Sig=260,4 Ref=360,100

Peak #	RetTime [min]	Type	Width [min]	Area [mAU*s]	Height [mAU]	Area %
1	10.040	BB	0.3543	1.01446e4	419.29840	49.3867
2	14.002	VB	0.5058	1.03965e4	301.90424	50.6133

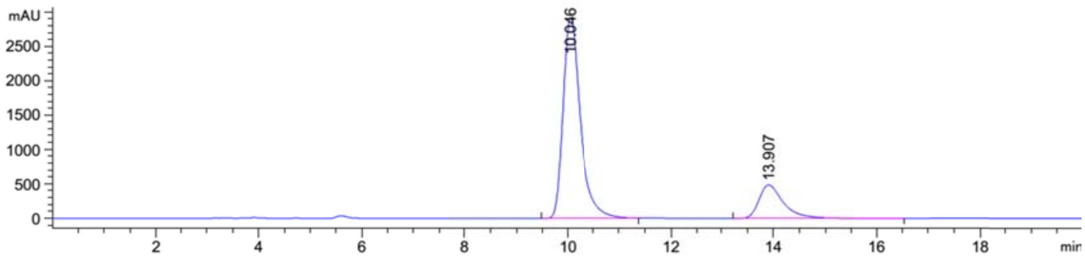

Signal 2: DAD1 B, Sig=260,4 Ref=360,100

Peak #	RetTime [min]	Type	Width [min]	Area [mAU*s]	Height [mAU]	Area %
1	10.046	BV	0.3537	6.89024e4	2916.13452	81.2117
2	13.907	BB	0.4913	1.59405e4	485.18893	18.7883

Supplementary Note 1

(Preparation of 4-aryl-l,2,4-triazoline-3,5-diones)[2-4]

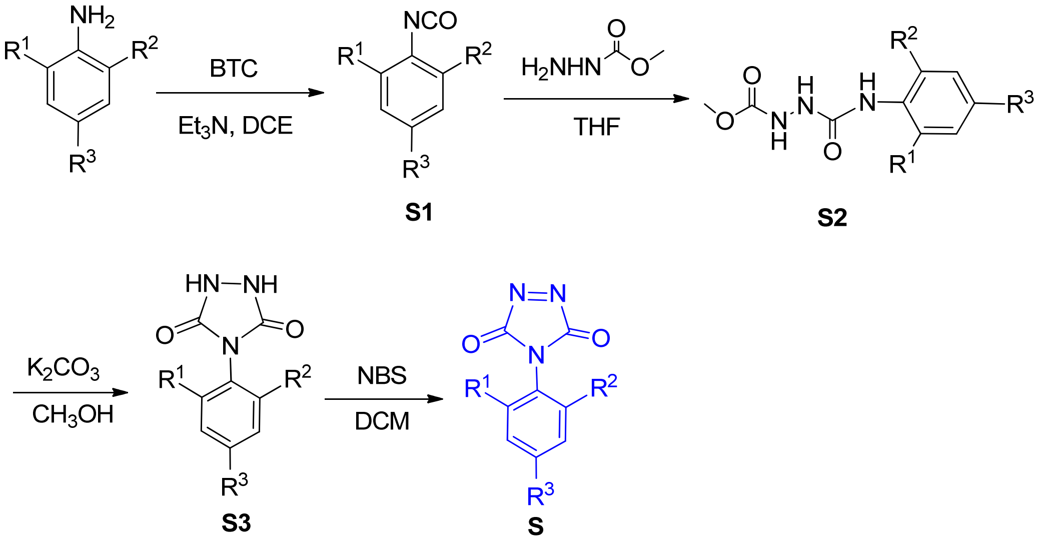

(Aryl isocyanate S1): Et_3N (0.03 mL, 0.01 equiv) was added to a stirring solution of triphosgene (2.97 g, 10.0 mmol, 0.5 equiv) in dry DCE (50 mL) at 0℃. After stirring 5 minutes, aromatic amine (20.0 mmol) in dry DCE (50 mL) was added slowly over 2 h. The reaction mixture was then refluxed and stirred under nitrogen for 4 h. After cooling to room temperature, the mixture was evaporated under reduced pressure to afford **S1** as a yellow liquid.

(4-Ar-1-carbethoxysemicarbazide S2): To a solution of methyl carbazate (1.8 g, 20.0 mmol) in anhydrous THF under argon atmosphere, aryl isocyante **S1** (20.0 mmol, 1.0 equiv.) was added over 1-2 minutes. The resulting mixture was stirred at room temperature for 1 h. After the reaction completion (monitored by TLC), the white solid product was collected by filtration or by simple evaporation to dryness.

(4-Ar-urazole S3): To a solution of intermediate **S2** in methanol, potassium carboxylate (2.0 equiv.) was added and the reaction mixture was refluxed overnight. After the reaction completion (monitored by TLC), the resulting mixture was condensed and re-dissolved with small amount of water. The pH of resulting mixture was adjusted to the range of 3~4 with the drop-wise addition of 1N aqueous HCl. The desired product **S3** was collected by simple filtration and washed with cold deionized water prior to drying.

(4-aryl-l,2,4-triazoline-3,5-diones S): N-Bromosuccinimide (20 mmol) was added to an ice-cold suspension of urazoles (10 mmol) in 150 mL of CH_2Cl_2. After being stirred for 30 min, the resulting red solution was extracted five times with water. The CH_2Cl_2 layer was then dried over

$MgSO_4$, filtered, and concentrated under reduced pressure. Purple or dark red solid of various triazolinediones were obtained.

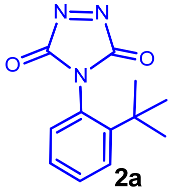
2a

4-(2-*tert*-butylphenyl)-3H-1,2,4-triazole-3,5-dione (**2a**) Yield 71%

^{1}H NMR (400 MHz, $CDCl_3$) δ 7.64 (dd, *J* = 8.4, 1.2 Hz, 1H), 7.49 (td, *J*=7.6, 1.2 Hz, 1H), 7.32 (td, *J*=8.0, 1.2 Hz, 1H), 6.85 (dd, *J* = 7.6, 1.2 Hz, 1H), 1.23 (s, 9H);

^{13}C NMR (100 MHz, $CDCl_3$) δ 158.5, 148.6, 130.9, 129.7, 129.2, 127.9, 127.0, 35.5, 31.5;

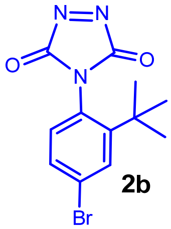
2b

4-(4-bromo-2-*tert*-butylphenyl)-3H-1,2,4-triazole-3,5-dione (**2b**) Yield 61%

^{1}H NMR (400 MHz, $CDCl_3$) δ 7.72 (d, *J* = 2.0 Hz, 1H), 7.47 (dd, *J* = 8.4, 2.0 Hz, 1H), 6.73 (d, *J* = 8.4 Hz, 1H), 1.23 (s, 9H);

^{13}C NMR (100 MHz, $CDCl_3$) δ 158.1, 150.9, 132.7, 131.3, 131.2, 126.2, 125.5, 35.8, 31.3;

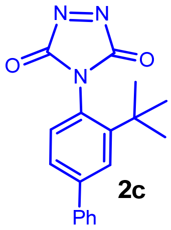
2c

4-(3-*tert*-butyl-[1,1'-biphenyl]-4-yl)-3H-1,2,4-triazole-3,5-dione (**2c**) Yield 40%

^{1}H NMR (400 MHz, $CDCl_3$) δ 7.85 (d, *J* = 2.0 Hz, 1H), 7.63-7.60 (m, 2H), 7.55 (dd, *J* = 8.0, 2.0 Hz, 1H), 7.53-7.49 (m, 2H), 7.46-7.43 (m, 1H), 6.95 (d, *J* = 8.4 Hz, 1H), 1.33 (s, 9H);

^{13}C NMR (100 MHz, $CDCl_3$) δ 158.5, 148.9, 144.0, 139.9, 130.1, 128.9, 128.2, 128.1, 127.3, 126.6, 126.0, 35.7, 31.5;

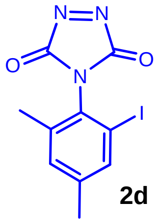
2d

4-(2-iodo-4,6-dimethylphenyl)-3H-1,2,4-triazole-3,5-dione (2d) Yield 64%

^{1}H NMR (400 MHz, $CDCl_3$) δ 7.60 (s, 1H), 7.14 (s, 1H), 2.33 (s, 3H), 2.10 (s, 3H);

^{13}C NMR (100 MHz, $CDCl_3$) δ 156.4, 143.0, 138.2, 137.4, 132.2, 127.6, 97.0, 20.6, 18.5;

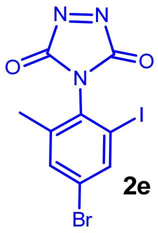

2e

4-(4-bromo-2-iodo-6-methylphenyl)-3H-1,2,4-triazole-3,5-dione (**2e**) Yield 56%

^{1}H NMR (400 MHz, $CDCl_3$) δ 7.92 (s, 1H), 7.50 (s, 1H), 2.13 (s, 3H);

^{13}C NMR (100 MHz, $CDCl_3$) δ 155.8, 139.9, 139.5, 134.4, 129.7, 125.6, 98.0, 18.6;

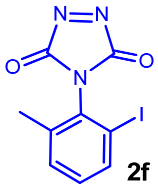

2f

4-(2-iodo-6-methylphenyl)-3H-1,2,4-triazole-3,5-dione (2f) Yield 52%

^{1}H NMR (400 MHz, $CDCl_3$) δ 7.76 (d, J = 7.6 Hz, 1H), 7.33 (d, J = 7.6 Hz, 1H), 7.14 (t, J = 8.0 Hz, 1H), 2.15 (s, 3H);

^{13}C NMR (100 MHz, $CDCl_3$) δ 156.2, 138.2, 137.7, 132.3, 131.4, 130.3, 97.3, 18.6;

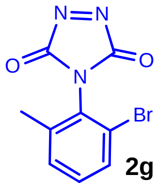

2g

4-(2-bromo-6-methylphenyl)-3H-1,2,4-triazole-3,5-dione (**2g**) Yield 62%

^{1}H NMR (400 MHz, $CDCl_3$) δ 7.56 (dd, J = 6.0, 3.2 Hz, 1H), 7.35-7.33 (m, 2H), 2.18 (s, 3H);

^{13}C NMR (100 MHz, $CDCl_3$) δ 156.3, 138.7, 132.0, 131.3, 130.5, 127.0, 122.0, 18.1;

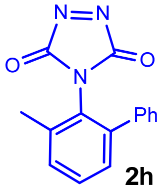

2h

4-(3-methyl-[1,1'-biphenyl]-2-yl)-3H-1,2,4-triazole-3,5-dione (**2h**) Yield 56%

^{1}H NMR (400 MHz, $CDCl_3$) δ 7.52 (t, J = 7.6 Hz, 1H), 7.41 (d, J = 7.6 Hz, 1H), 7.35-7.31 (m, 4H), 7.14-7.11 (m, 2H), 2.19 (s, 3H);

^{13}C NMR (100 MHz, $CDCl_3$) δ 157.2, 141.3, 137.1, 136.2, 130.5, 128.8, 128.6, 128.2, 127.7, 125.6, 17.6;

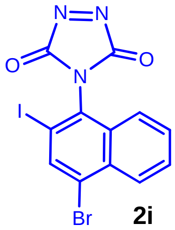

2i

4-(4-bromo-2-iodonaphthalen-1-yl)-3H-1,2,4-triazole-3,5-dione (**2i**) Yield 44%

^{1}H NMR (400 MHz, $CDCl_3$) δ 8.35 (d, J = 8.4 Hz, 1H), 8.31 (s, 1H), 7.77-7.73 (m, 1H), 7.66-7.62 (m, 1H), 7.33 (d, J = 8.4 Hz, 1H);
^{13}C NMR (100 MHz, $CDCl_3$) δ 156.3, 138.0, 132.7, 132.5, 131.0, 130.0, 129.1, 128.7, 127.1, 121.7, 96.

Supplementary Note 2

General procedure for asymmetric synthesis of axially chiral urazoles

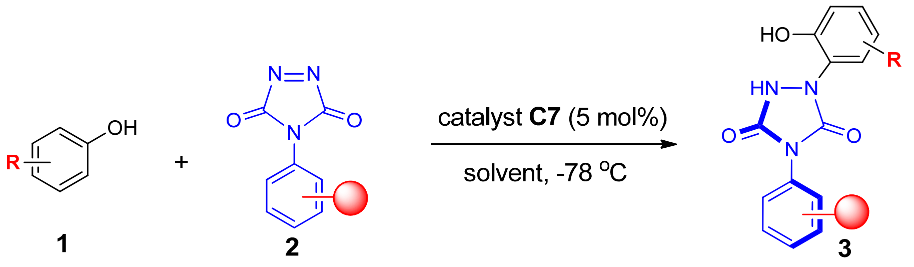

In a Schlenk tube 4-aryl-l,2,4-triazoline-3,5-diones **2** (0.12 mmol) and catalyst **C7** (5 mol%, 0.005 mmol) were dissolved in Et_2O (2 mL). The solution was stirred for 10 min at -78 °C before 2-naphthols and phenols **1** (0.10 mmol) were added. The resulting solution was stirred at- 78 °C until red color disappeared. After monitored by TLC, the reaction mixture was acidified with 6 N HCl and concentrated. Then the obtained crude material was purified by silica gel column chromatography (CH_2Cl_2 to CH_2Cl_2/Acetone = 10/1) to afford the pure products **3**.

In some cases, reactions were performed with 20 mol% of catalyst **C7** in 2.0 mL solvent, for **3d** in DCM at -78℃; **3i** and **3j** in dry toluene at -40℃.

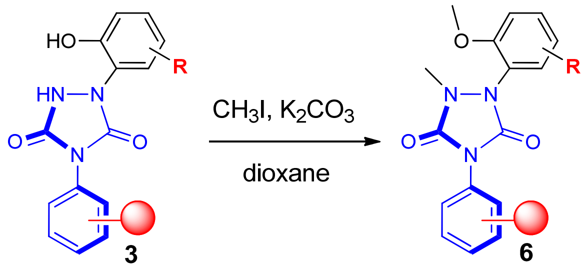

Potassium carbonate (0.4 mmol, 2.0 equiv) was added to a solution of products **3** (0.2 mmol, 1.0 equiv) and iodomethane (2.0 mmol, 10 equiv) in dioxane (1 mL) at room temperature. The solution was stirred at room temperature for 5 h， then the product was afforded by silica gel flash column chromatography (EtOAc/Hexane = 1/2).

Products **6** were synthesized for NMR spectra of **3** (except **3i** and **3j**), **6p'** for X-ray single crystal diffraction. **(Note: The NMR spectra for most of products 3 were displayed very messy and peaks splitting were not clear. Thus, the products 6 were synthesized for better NMR spectra collection.)**

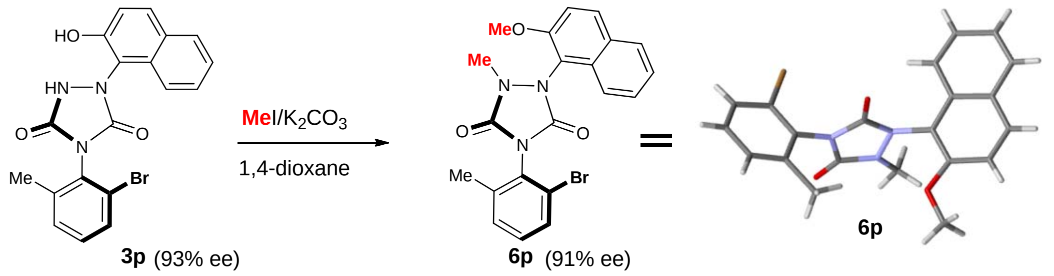

Potassium carbonate (0.4 mmol, 2.0 equiv) was added to a solution of products **3p** (0.2 mmol, 1.0 equiv) and iodomethane (2.0 mmol, 10 equiv) in dioxane (1 mL) at room temperature. The solution was stirred at room temperature for 5 h，then the product **6p** was afforded by silica gel flash column chromatography (EtOAc/Hexane = 1/2) with 60% yield.

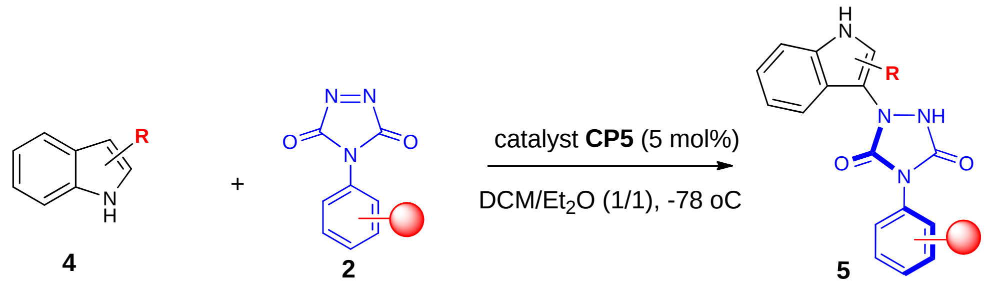

In a Schlenk tube 4-aryl-l,2,4-triazoline-3,5-diones **2** (0.12 mmol) and catalyst **CP5** (5 mol%, 0.005 mmol) were dissolved in DCM/Et_2O = 1/1 (2 mL). The solution was stirred for 10 min at -78 °C before 2-substituted indole **4** (0.10 mmol) was added. The resulting solution was stirred under this condition until purple colour disappeared. After monitored by TLC, the reaction mixture was concentrated，and then purified by silica gel column chromatography (CH_2Cl_2 /Acetone = 20/1) to afford the pure products **5**.

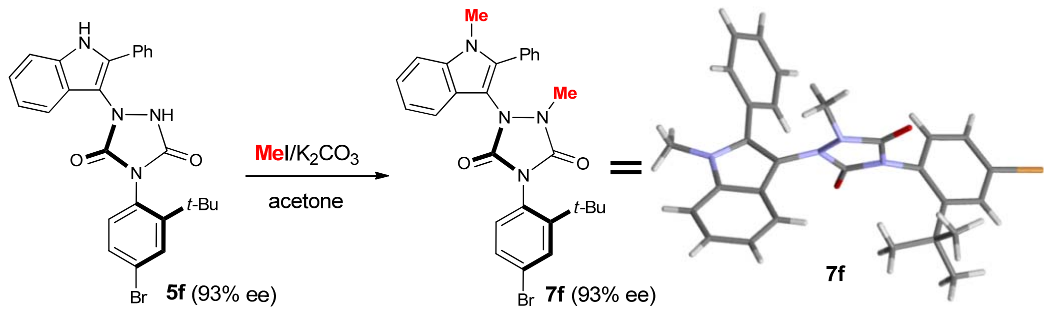

Potassium carbonate (0.4 mmol, 2.0 equiv) was added to a solution of above product **5f** (0.2 mmol, 1.0 equiv) and iodomethane (2.0 mmol, 10 equiv) in acetone (1 mL) at room temperature. The solution was stirred at room temperature for 5 h，then the product **7f** was afforded by silica gel flash column chromatography or PTLC (EtOAc/Hexane = 1/4).

7f was synthesized for X-ray single crystal diffraction.

Supplementary Note 3

(Gram-scale synthesis of 3a and 5a)

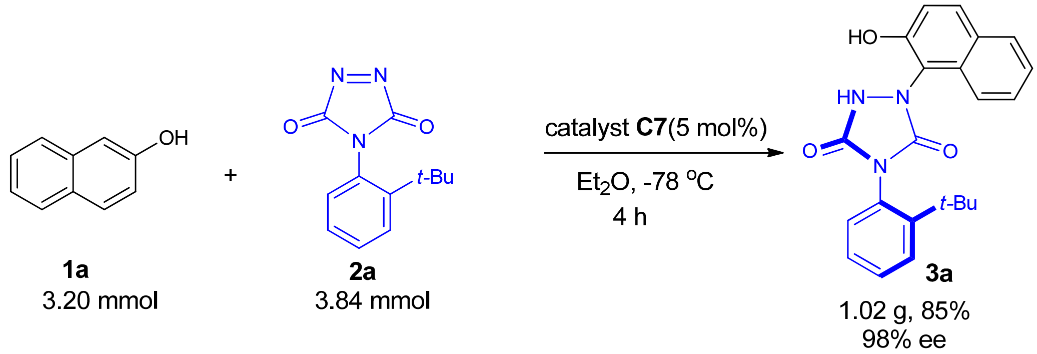

In a 100 mL round flask 4-(2-(*tert*-butyl)phenyl)-1,2,4-triazole-3,5-dione **2a** (3.84 mmol) and catalyst **C7** (5 mol%, 0.16 mmol) were dissolved in 65 mL Et_2O. The solution was stirred for 20 min at -78 °C before 2-naphthol **1a** (3.20 mmol) was added. The resulting solution was stirred under this condition until red colour disappeared. After monitored by TLC, the reaction mixture was acidified with 6 N HCl and concentrated. Then the obtained crude material was purified by silica gel column chromatography (CH_2Cl_2 to CH_2Cl_2/acetone = 10/1) to afford pure product **3a** as white solid. (1.02 g, 85% yield, 98% ee)

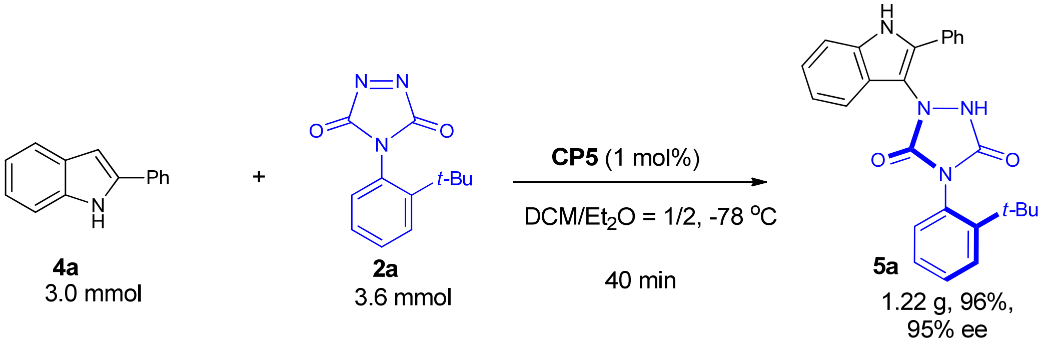

In a 100mL round flask 4-(2-(*tert*-butyl)phenyl)-1,2,4-triazole-3,5-dione **2a** (3.60 mmol) and **CP5** (1 mol%, 0.03 mmol) were dissolved in DCM/Et_2O = 1/2 (60 mL). The solution was stirred for 20 min at -78 °C before 2-phenyl-indole **4a** (3.00 mmol) was added. The resulting solution was stirred under this condition until purple color disappeared. After monitored by TLC, the reaction mixture was concentrated, and then purified by silica gel column chromatography (CH_2Cl_2 /Acetone = 20:1) to afford pure product **5a** as white solid. (1.22 g, 96% yield, 95% ee)

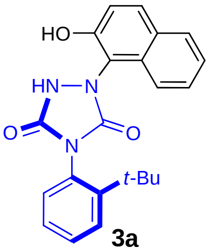

3a

4-(2-(*tert*-butyl)phenyl)-1-(2-hydroxynaphthalen-1-yl)-1,2,4-triazolidine-3,5-dione (3a) Yield 82%, 99% ee

HPLC analysis [Daicel CHIRALPAK AD-H column (250 mm × 4.6 mm), hexane/*i*-PrOH = 85/15, 0.8 mL/min, T = 25 °C, λ = 230nm, t_R (major) = 10.2 min, t_R (minor) = 17.4 min];

HRMS (ESI) calcd for $C_{22}H_{21}N_3O_3Na^+$ $(M+Na)^+$ 398.1475, found 398.1473;

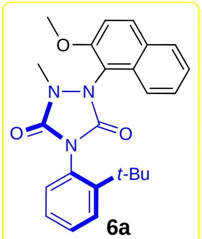

6a

^{1}H NMR (400 MHz, $CDCl_3$) δ 8.15 (d, *J* = 8.8 Hz, 1H), 8.04 (d, *J* = 8.8 Hz, 1H), 7.88 (d, *J* = 8.4 Hz, 1H), 7.67-7.61 (m, 2H), 7.47 (t, *J* = 7.6 Hz, 2H), 7.39 (m, 2H), 7.42-7.36 (d, *J* = 7.6 Hz, 1H), 4.07 (s, 3H), 3.09 (s, 3H), 1.52 (s, 9H);

^{13}C NMR (100 MHz, $CDCl_3$) δ 155.4, 154.6, 154.1, 149.6, 134.2, 132.7, 131.8, 130.3, 129.3, 129.1, 128.7, 128.5, 127.7, 125.8, 124.9, 121.6, 113.6, 112.1, 57.1, 36.2, 32.0, 31.7;

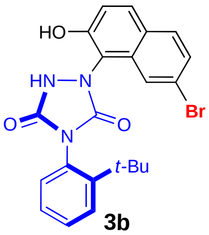

3b

1-(7-bromo-2-hydroxynaphthalen-1-yl)-4-(2-(*tert*-butyl)phenyl)-1,2,4-triazolidine-3,5-dione (3b) Yield 85%, 99% ee

HPLC analysis [Daicel CHIRALPAK AD-H column (250 mm × 4.6 mm), hexane/*i*-PrOH = 85/15, 1.0 mL/min, T = 25 °C, λ = 230nm, t_R (major) = 7.8 min, t_R (minor) = 23.0 min].

HRMS (ESI) calcd for $C_{22}H_{20}BrN_3O_3Na^+$ $(M+Na)^+$ 476.0580, found 476.0579;

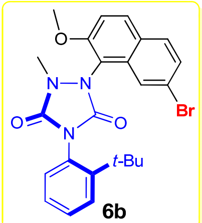

6b

^{1}H NMR (400 MHz, $CDCl_3$) δ 8.24 (s, 1H), 7.95 (d, *J* = 9.2 Hz, 1H), 7.70 (d, *J* = 8.4 Hz, 1H), 7.64 (dd, *J* = 8.0, 1.2 Hz, 1H), 7.50 (dd, *J* = 4.8, 1.6 Hz, 1H), 7.47-7.42 (m, 1H), 7.37-7.33 (m, 2H), 7.17 (dd, *J* = 7.6, 1.2 Hz, 1H), 4.03 (s, 3H), 3.07 (s, 3H), 1.51 (s, 9H);

^{13}C NMR (100 MHz, $CDCl_3$) δ 156.0, 154.2, 153.9, 149.4, 135.0, 132.5, 131.4, 130.1, 130.0, 129.6, 128.9, 128.2, 127.4, 127.3, 123.5, 123.4, 115.6, 113.7, 56.9, 36.0, 31.8, 31.6;

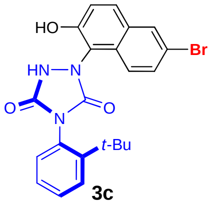

3c

1-(6-bromo-2-hydroxynaphthalen-1-yl)-4-(2-(*tert*-butyl)phenyl)-1,2,4-triazolidine-3,5-dione (3c) Yield 81%, 99% ee

HPLC analysis [Daicel CHIRALPAK AD-H column (250 mm × 4.6 mm), hexane/*i*-PrOH = 85/15, 1.0 mL/min, T = 25 °C, λ = 230nm, t_R (major) = 9.6 min, t_R (minor) = 17.4 min];

HRMS (ESI) calcd for $C_{22}H_{20}BrN_3O_3^+$ $(M+Na)^+$ 476.0580, found 476.0581;

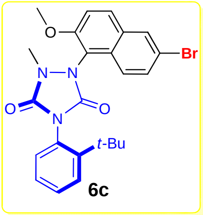

6c

^{1}H NMR (400 MHz, $CDCl_3$) δ 8.04 (s, 1H), 8.03 (d, *J* = 8.8 Hz, 1H), 7.94 (d, *J* = 8.8 Hz, 1H), 7.70-7.65 (m, 2H), 7.47 (td, *J* = 7.0, 1.6 Hz, 1H), 7.42 (d, *J* = 9.2 Hz, 1H), 7.37 (td, *J* = 9.0, 1.2 Hz, 1H), 7.18 (dd, *J* = 7.6, 1.2 Hz, 1H), 4.06 (s, 3H), 3.07 (s, 3H), 1.51 (s, 9H);

^{13}C NMR (100 MHz, $CDCl_3$) δ 155.3, 154.4, 153.9, 149.3, 132.6, 131.8, 131.5, 131.4, 130.1, 130.1, 130.0, 129.5, 128.9, 127.5, 123.2, 118.4, 117.0, 114.5, 56.9, 35.9, 31.7, 31.6;

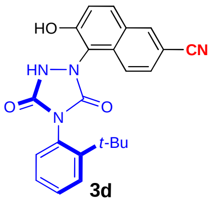

3d

5-(4-(2-(*tert*-butyl)phenyl)-3,5-dioxo-1,2,4-triazolidin-1-yl)-6-hydroxy -2-naphthonitrile (3d) Yield 61%, 98% ee

HRMS (ESI) calcd for $C_{23}H_{20}N_4O_3Na^+$ $(M+Na)^+$ 423.1428, found 423.1429;

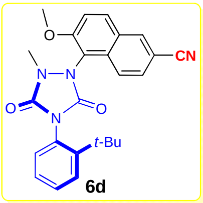

6d

HPLC analysis [Daicel CHIRALPAK IC column (250 mm × 4.6 mm), hexane/*i*-PrOH =70/30, 1.0 mL/min, T= 25 °C, λ= 240nm, t_R (minor) = 33.6 min, t_R (major) = 43.4 min];

^{1}H NMR (400 MHz, $CDCl_3$) δ 8.28 (s, 1H), 8.26 (d, *J* = 8.8 Hz, 1H), 8.12 (d, *J* = 9.2 Hz, 1H), 7.76 (dd, *J* = 8.8, 1.2 Hz, 1H), 7.66 (dd, *J* = 8.4, 1.2 Hz, 1H), 7.53 (d, *J* = 9.2 Hz, 1H), 7.47 (td, *J* = 8.0, 1.6 Hz, 1H), 7.37 (td, *J* = 7.6, 1.6 Hz, 1H), 7.16 (dd, *J* = 7.6, 1.6 Hz, 1H), 4.12 (s, 3H), 3.08 (s, 3H), 1.50 (s, 9H);

^{13}C NMR (100 MHz, $CDCl_3$) δ 157.4, 154.5, 154.0, 149.2, 135.6, 134.5, 133.2, 131.3, 130.2, 129.4, 129.0, 128.9, 127.6, 127.5, 122.7, 118.9, 117.2, 115.1, 108.1, 57.0, 35.9, 31.9, 31.7;

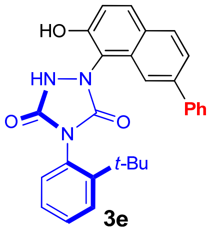

3e

4-(2-(*tert*-butyl)phenyl)-1-(2-hydroxy-7-phenylnaphthalen-1-yl)-1,2,4 - triazolidine-3,5-dione (3e) Yield 76%, 97% ee

HPLC analysis [Daicel CHIRALPAK AD-H column (250 mm × 4.6 mm), hexane/*i*-PrOH = 85/15, 1.0 mL/min, T = 25 °C, λ = 254nm, t_R (major) = 7.7 min, t_R (minor) = 18.3 min];

HRMS (ESI) calcd for $C_{28}H_{25}N_3O_3Na^+$ $(M+Na)^+$ 474.1788, found 474.1789;

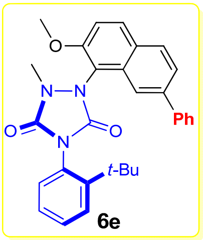

6e

^{1}H NMR (400 MHz, $CDCl_3$) δ 8.22 (d, *J* = 0.8 Hz, 1H), 8.03 (d, *J* = 9.2 Hz, 1H), 7.92 (d, *J* = 8.4 Hz, 1H), 7.73-7.67 (m, 3H), 7.63 (dd, *J* = 8.4, 1.6 Hz, 1H), 7.55-7.51 (m, 2H), 7.46-7.42 (m, 2H), 7.38-7.33 (m, 2H), 7.27 (dd, *J* = 8.0, 1.6 Hz, 1H), 4.02 (s, 3H), 3.10 (s, 3H), 1.52 (s, 9H);

^{13}C NMR (100 MHz, $CDCl_3$) δ 155.3, 154.1, 152.7, 149.6, 141.4, 141.0, 134.5, 132.4, 131.9, 130.0, 129.0, 128.8, 128.5, 128.1, 127.9, 127.7, 127.5, 124.5, 119.3, 115.3, 112.8, 56.2, 35.7, 31.6, 31.4;

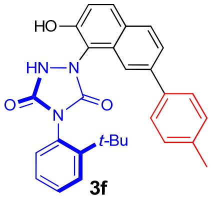

3f

4-(2-(*tert*-butyl)phenyl)-1-(2-hydroxy-7-(p-tolyl)naphthalen-1-yl)-1,2,4- triazolidine-3,5-dione (3f) Yield 71%, 99% ee;
HPLC analysis [Daicel CHIRALPAK AD-H column (250 mm × 4.6 mm), hexane/*i*-PrOH = 85/15, 1.0 mL/min, T = 25 °C, λ = 260nm, t_R (major) = 7.5 min, t_R (minor) = 26.5 min];
HRMS (ESI) calcd for $C_{29}H_{27}N_3O_3Na^+$ $(M+Na)^+$ 488.1945, found 488.1943;

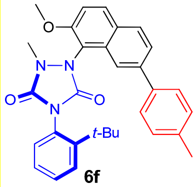

6f

^{1}H NMR (400 MHz, $CDCl_3$) δ 8.22 (s, 1H), 8.05 (d, J = 9.2 Hz, 1H), 7.93 (d, J = 8.4 Hz, 1H), 7.70 (d, J = 8.4 Hz, 1H), 7.67-7.63 (m, 3H), 7.46 (t, J = 7.6 Hz, 1H), 7.40-7.35 (m, 4H), 7.29 (s, 1H), 4.04 (s, 3H), 3.11 (s, 3H), 2.48 (s, 3H), 1.54 (s, 9H);
13C NMR (100 MHz, $CDCl_3$) δ 155.2, 154.1, 152.7, 149.6, 141.3, 138.1, 137.8, 134.5, 132.3, 131.9, 130.0, 129.7, 128.7, 128.5, 128.0, 127.5, 124.5, 119.0, 115.2, 112.7, 56.2, 35.7, 31.6, 31.4, 21.2;

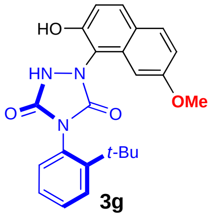

3g

4-(2-(*tert*-butyl)phenyl)-1-(2-hydroxy-7-methoxynaphthalen-1-yl)-1,2,4- triazolidine-3,5-dione (3g) Yield 81%, 97% ee
HPLC analysis [Daicel CHIRALPAK AD-H column (250 mm × 4.6 mm), hexane/*i*-PrOH = 85/15, 1.0 mL/min, T = 25 °C, λ = 230nm, t_R(minor) = 20.0 min, t_R(major) = 25.5 min];
HRMS (ESI) calcd for $C_{23}H_{23}N_3O_4Na^+$ $(M+Na)^+$ 428.1581, found 428.1576;

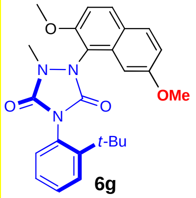

6g

^{1}H NMR (400 MHz, $CDCl_3$) δ 7.90 (d, J = 9.2 Hz, 1H), 7.74 (d,J = 9.2 Hz, 1H), 7.63 (dd, J = 8.0, 0.8 Hz, 1H), 7.46-7.42 (m, 1H), 7.37-7.34 (m, 2H), 7.21-7.18 (m, 2H), 7.08 (dd, J = 8.8, 2.8 Hz, 1H), 4.01 (s, 3H), 3.93 (s, 3H), 3.07 (s, 3H), 1.50 (s, 9H);
^{13}C NMR (100 MHz, $CDCl_3$) δ 159.9, 155.9, 153.6, 153.5, 149.1, 135.6, 132.1, 131.6, 130.0, 129.6, 128.9, 127.5, 124.6, 117.4, 114.8, 110.4, 99.5, 56.7, 55.3, 35.9, 31.7, 31.2;

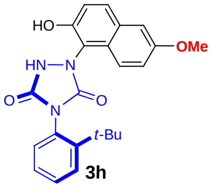

3h

4-(2-(*tert*-butyl)phenyl)-1-(2-hydroxy-6-methoxynaphthalen-1-yl)-1,2,4- triazolidine-3,5-dione (3h) Yield 70%, 98% ee
HPLC analysis [Daicel CHIRALPAK AD-H column (250 mm × 4.6 mm), hexane/*i*-PrOH = 85/15, 1.0 mL/min, T = 25 °C, λ = 230nm, t_R (minor) = 18.1 min, t_R (major) = 25.4 min].
HRMS (ESI) calcd for $C_{23}H_{23}N_3O_4Na^+$ $(M+Na)^+$ 428.1581, found 428.1580;

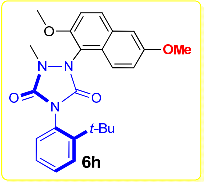

^{1}H NMR (400 MHz, $CDCl_3$) δ 8.05 (d, *J* = 9.2 Hz, 1H), 7.91 (d, *J* = 9.2 Hz, 1H), 7.65 (dd, *J* = 8.0, 1.2 Hz, 1H), 7.46 (td, *J* = 8.0, 1.6 Hz, 1H), 7.39-7.35 (m, 2H), 7.31 (dd, *J* = 9.2, 2.4 Hz, 1H), 7.20 (dd, *J* = 7.6, 1.6 Hz, 1H), 7.17 (d, *J* = 2.4 Hz, 1H), 4.03 (s, 3H), 3.95 (s, 3H), 3.08 (s, 3H), 1.51 (s, 9H);

^{13}C NMR (100 MHz, $CDCl_3$) δ 156.8, 154.4, 153.8, 153.4, 149.3, 131.5, 130.8, 130.1, 130.0, 129.7, 129.3, 128.8, 127.4, 123.0, 121.4, 117.1, 114.1, 106.1, 56.9, 55.4, 35.9, 31.7, 31.4;

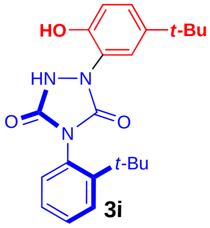

1-(5-(*tert*-butyl)-2-hydroxyphenyl)-4-(2-(tert-butyl)phenyl)-1,2,4-triazolidine-3,5-dione (3i) Yield 51%, 94% ee

HPLC analysis [Daicel CHIRALPAK AD-H column (250 mm × 4.6 mm), hexane/*i*-PrOH = 95/5, 1.2 mL/min, T = 25 °C, λ = 214nm, t_R (major) = 10.9 min, t_R (minor) = 19.9 min];

HRMS (ESI) calcd for $C_{22}H_{27}N_3O_3Na^+$ $(M+Na)^+$ 404.1945, found 404.1944;

^{1}H NMR (400 MHz, $CDCl_3$) δ 7.65 (d, *J* = 8.0 Hz, 1H), 7.49 (t, *J* = 7.6 Hz, 1H), 7.35-7.33 (m, 2H), 7.23 (d, *J* = 8.8 Hz, 1H), 7.11 (d, *J* = 7.6 Hz, 1H), 6.99 (d, *J* = 8.4 Hz, 1H), 1.40 (s, 9H), 1.23 (s, 9H);

^{13}C NMR (100 MHz, $CDCl_3$) δ 154.1, 151.0, 149.2, 146.0, 144.6, 131.3, 130.7, 129.1, 127.8, 127.6, 125.9, 123.6, 119.6, 118.1, 35.9, 34.3, 31.6, 31.3;

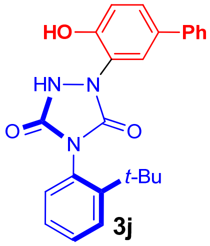

4-(2-(*tert*-butyl)phenyl)-1-(4-hydroxy-[1,1'-biphenyl]-3-yl)-1,2,4-triazolidine-3,5-dione (3j) Yield 60%, 90% ee

HPLC analysis [Daicel CHIRALPAK AD-H column (250 mm × 4.6 mm), hexane/*i*-PrOH = 85/15, 1.0 mL/min, T = 25 °C, λ = 254nm, t_R (major) = 6.1 min, t_R (minor) = 9.8 min];

HRMS (ESI) calcd for $C_{24}H_{24}N_3O_3^+$ $(M+H)^+$ 402.1812, found 402.1815;

^{1}H NMR (400 MHz, $CDCl_3$) δ 7.65 (dd, *J* = 8.0, 1.2 Hz, 1H), 7.60 (d, *J* = 2.0 Hz, 1H), 7.52-7.44 (m, 4H), 7.40-7.33 (m, 4H), 7.15 (d, *J* = 8.4 Hz, 1H), 7.13 (d, *J* = 8.0 Hz, 1H), 1.37 (s, 9H);

^{13}C NMR (100 MHz, $CDCl_3$) δ 154.2, 151.6, 149.1, 147.7, 139.6, 134.9, 131.2, 130.8, 129.2, 128.9, 127.7, 127.6, 127.4, 127.3, 126.8, 124.9, 120.7, 119.3, 35.9, 31.6;

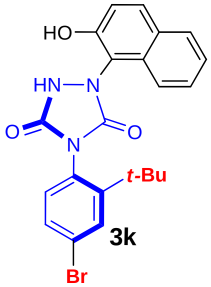

4-(4-bromo-2-(*tert*-butyl)phenyl)-1-(2-hydroxynaphthalen-1-yl)-1,2,4-triazolidine-3,5-dione (3k) Yield 70%, 99% ee

HPLC analysis [Daicel CHIRALPAK AD-H column (250 mm × 4.6 mm), hexane/*i*-PrOH = 85/15, 0.8mL/min, T = 25 °C, λ = 230nm, t_R (major) = 9.1 min, t_R (minor) = 34.1 min];

HRMS (ESI) calcd for $C_{22}H_{20}BrN_3O_3^+$ $(M+Na)^+$ 476.0580, found 476.0579;

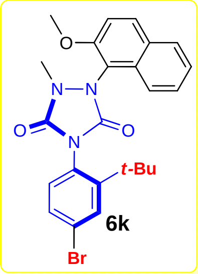

^{1}H NMR (400 MHz, $CDCl_3$) δ 8.11 (d, J = 8.8 Hz, 1H), 8.05 (d, J = 9.2 Hz, 1H), 7.89 (d, J = 8.0 Hz, 1H), 7.77 (d, J = 2.4 Hz, 1H), 7.63 (td, J = 6.8, 1.2 Hz, 1H), 7.52 (dd, J = 8.4, 2.4 Hz, 1H), 7.47 (td, J = 7.8, 1.2 Hz, 1H), 7.40 (d, J = 9.2 Hz, 1H), 7.07 (d, J = 8.43 Hz, 1H), 4.06 (s, 3H), 3.08 (s, 3H), 1.50 (s, 9H);

^{13}C NMR (100 MHz, $CDCl_3$) δ 155.1, 153.8, 153.3, 151.7, 133.9, 133.2, 132.6, 132.2, 130.7, 129.0, 128.9, 128.5, 128.3, 124.7, 124.4, 121.2, 116.2, 113.3, 56.9, 36.1, 31.5, 31.3;

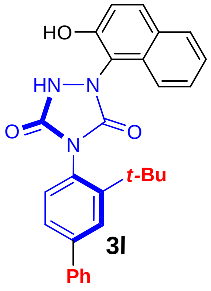

4-(3-(*tert*-butyl)-[1,1'-biphenyl]-4-yl)-1-(2-hydroxynaphthalen-1-yl)-1,2,4-triazolidine-3,5-dione (3l) Yield 64%, 98% ee

HPLC analysis [Daicel CHIRALPAK AD-H column (250 mm × 4.6 mm), T = 25 °C, λ = 230nm, hexane/*i*-PrOH = 80/20, 1mL/min,t_R (major) = 5.6 min, t_R (minor) = 17.9 min];

HRMS (ESI) calcd for $C_{28}H_{25}N_3O_3Na^+$ $(M+Na)^+$ 474.1788, found 474.1790;

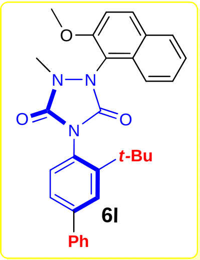

^{1}H NMR (400 MHz, $CDCl_3$) δ 8.14 (d, J = 10 Hz, 1H), 8.06 (d, J = 9.2 Hz, 1H), 7.89 (d, J = 8.4 Hz, 1H), 7.83 (d, J = 2.0 Hz, 1H), 7.66-7.62 (m, 3H), 7.57 (dd, J = 8.0, 2.0 Hz, 1H), 7.51-7.39 (m, 5H), 7.34 (d, J = 8.0 Hz, 1H), 4.05 (s, 3H), 3.09 (s, 3H), 1.58 (s, 9H);

^{13}C NMR (100 MHz, $CDCl_3$) δ 154.9, 154.1, 152.6, 149.7, 143.0, 141.0, 134.2, 132.6, 132.2, 129.2, 129.0, 128.8, 128.5, 128.2, 127.8, 127.6, 127.5, 126.4, 124.6, 121.5, 115.1, 112.9, 56.2, 35.99, 31.6, 31.4;

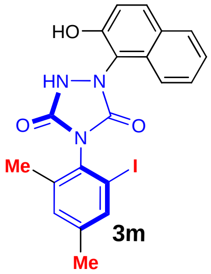

1-(2-hydroxynaphthalen-1-yl)-4-(2-iodo-4,6-dimethylphenyl)-1,2,4-triazolidine-3,5-dione (3m) Yield 68%, 91% ee

HPLC analysis [Daicel CHIRALPAK AD-H column (250 mm × 4.6 mm), hexane/*i*-PrOH = 85/15, 1mL/min, T = 25 °C, λ = 230nm, t_R (major) = 15.0 min, t_R (minor) = 21.3 min];

HRMS (ESI) calcd for $C_{20}H_{16}IN_3O_3Na^+$ $(M+Na)^+$ 496.0129, found 496.0131;

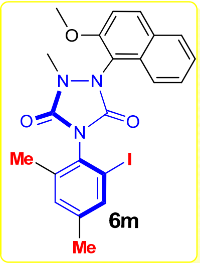

^{1}H NMR (400 MHz, $CDCl_3$) δ 8.05 (d, J = 9.2 Hz, 1H), 8.00 (d, J = 8.8 Hz, 1H), 7.89 (d, J = 8.0 Hz, 1H), 7.68 (s, 1H), 7.62 (td, J = 7.6, 1.2 Hz, 1H), 7.46 (td, J = 7.6, 0.8 Hz, 1H), 7.42 (d, J = 9.2 Hz, 1H), 7.16 (s, 1H), 4.06 (s, 3H), 3.10 (s, 3H), 2.43 (s, 3H), 2.35 (s, 3H);

^{13}C NMR (100 MHz, $CDCl_3$) δ 156.0, 152.0, 151.0, 141.9, 138.8, 138.5, 134.1, 133.0, 132.3, 130.7, 129.3, 128.7, 128.6, 124.8, 121.4, 115.1, 113.8, 99.5, 57.1, 31.8, 21.0, 19.3;

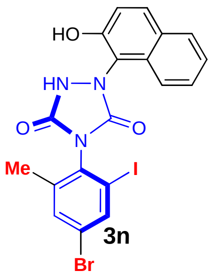

4-(4-bromo-2-iodo-6-methylphenyl)-1-(2-hydroxynaphthalen-1-yl)-1,2,4-triazolidine-3,5-dione (3n) Yield 70%, 90% ee

HRMS (ESI) calcd for $C_{19}H_{13}BrIN_3O_3Na^+$ $(M+Na)^+$ 559.9077, found 559.9077;

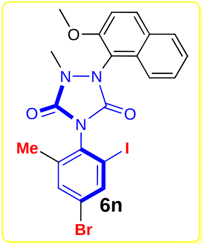

HPLC analysis [Daicel CHIRALPAK IC column (250 mm × 4.6 mm), hexane/*i*-PrOH = 70/30, 1mL/min, T = 25 °C, λ = 240nm, t_R (minor) = 18.3 min, t_R (major) = 24.8 min];

^{1}H NMR (400 MHz, $CDCl_3$) δ 8.19 (d, *J* = 8.4 Hz, 1H), 8.06 (d, *J* = 9.2 Hz, 1H), 7.98 (d, *J* = 2.0 Hz, 1H), 7.89 (d, *J* = 8.4 Hz, 1H), 7.64 (t, *J* = 7.6 Hz, 1H), 7.53 (d, *J* = 1.2 Hz, 1H), 7.47 (t, *J* = 7.2 Hz, 1H), 7.41 (d, *J* = 9.2 Hz, 1H), 4.01 (s, 3H), 3.10 (s, 3H), 2.47 (s, 3H);

^{13}C NMR (100 MHz, $CDCl_3$) δ 1550, 151.3, 150.2, 141.0, 139.4, 134.0, 134.0, 133.6, 132.7, 128.9, 128.6, 128.2, 124.7, 124.4, 121.4, 115.2, 113.0, 100.8, 56.4, 31.6, 18.7;

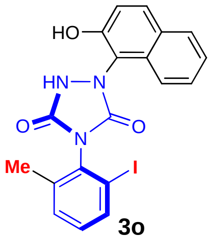

1-(2-hydroxynaphthalen-1-yl)-4-(2-iodo-6-methylphenyl)-1,2,4-triazolidine-3,5-dione (3o) Yield 70%, 98% ee

HPLC analysis [Daicel CHIRALPAK AD-H column (250 mm × 4.6 mm), hexane/*i*-PrOH = 85/15, 1mL/min, T = 25 °C, λ = 230nm, t_R (major) = 17.1 min, t_R (minor) = 25.3 min];

HRMS (ESI) calcd for $C_{19}H_{14}IN_3O_3Na^+$ $(M+Na)^+$ 481.9972, found 481.9974;

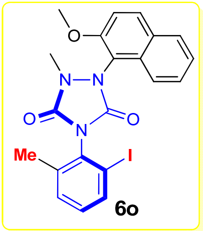

^{1}H NMR (400 MHz, $CDCl_3$) δ 8.06 (d, *J* = 9.2 Hz, 1H), 8.01 (d, *J* = 8.4 Hz, 1H), 7.89 (d, *J* = 8.0 Hz, 1H), 7.85 (d, *J* = 7.6 Hz, 1H), 7.63 (t, *J* = 7.6 Hz, 1H), 7.47 (td, *J* = 7.6, 0.8 Hz,1H), 7.43 (d, *J* = 9.2 Hz, 1H), 7.35 (d, *J* = 7.6 Hz, 1H), 7.11 (t, *J* = 7.6 Hz, 1H), 4.07 (s, 3H), 3.11 (s, 3H), 2.48 (s, 3H);

^{13}C NMR (100 MHz, $CDCl_3$) δ 155.7, 151.5, 150.5, 139.3, 137.8, 133.8, 133.1, 132.8, 131.3, 131.1, 129.0, 128.5, 128.4, 124.6, 121.1, 114.7, 113.5, 99.5, 56.8, 31.5, 19.1;

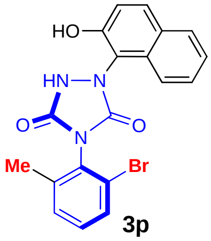

4-(2-bromo-6-methylphenyl)-1-(2-hydroxynaphthalen-1-yl)-1,2,4-triazolidine-3,5-dione (3p) Yield 70%, 93% ee

HPLC analysis [Daicel CHIRALPAK AD-H column (250 mm × 4.6 mm), hexane/*i*-PrOH = 85/15, 1mL/min, T = 25 °C, λ = 230nm, t_R (major) = 21.2 min, t_R (minor) = 28.2 min];

HRMS (ESI) calcd for $C_{19}H_{14}BrN_3O_3Na^+$ $(M+Na)^+$ 434.0111, found 434.0111;

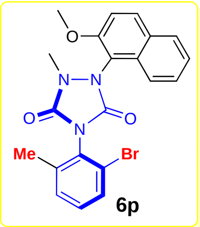

4-(2-bromo-6-methylphenyl)-1-(2-methoxynaphthalen-1-yl)-2-methyl -1,2,4-triazolidine-3,5-dione (6p) Yield 37%, 85% ee

HPLC analysis [Daicel CHIRALPAK ID column (250 mm × 4.6 mm), hexane/*i*-PrOH = 60/40, 1mL/min, T = 25 °C, λ = 230nm, t_R (minor) = 34.9 min, t_R (major) = 44.6 min];

^{1}H NMR (400 MHz, $CDCl_3$) δ 8.07 (d, *J* = 4.4 Hz, 1H), 8.05 (d, *J* =4.8 Hz, 1H), 7.89 (d, *J* = 8.4 Hz, 1H), 7.65-7.61 (m, 2H), 7.46 (td, *J* = 8.0, 1.2 Hz, 1H), 7.42 (d, *J* = 9.2 Hz, 1H), 7.34 (d, *J* = 7.2 Hz, 1H), 7.29 (t, *J* = 8.0 Hz, 1H), 4.05 (s, 3H), 3.10 (s, 3H), 2.47 (s, 3H);

^{13}C NMR (100 MHz, $CDCl_3$) δ155.8, 152.0, 151.5, 140.1, 134.2, 133.0, 131.6, 131.2, 130.4, 129.9, 129.3, 128.7, 128.6, 124.8, 124.3, 121.4, 115.5, 113.7, 57.0, 31.8, 19.0;

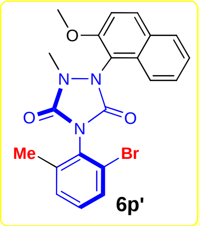

4-(2-bromo-6-methylphenyl)-1-(2-methoxynaphthalen-1-yl)-2-methyl -1,2,4-triazolidine-3,5-dione (6p') Yield 55%, 91% ee

HPLC analysis [Daicel CHIRALPAK IC column (250 mm × 4.6 mm), hexane/*i*-PrOH = 70/30, 1mL/min, T = 25 °C, λ = 230nm, t_R (major) = 19.7 min, t_R (minor) =28.7 min];

^{1}H NMR (400 MHz, $CDCl_3$) δ 8.19 (d, *J* = 8.8 Hz, 1H), 8.05 (d, *J* = 8.8 Hz, 1H), 7.88 (d, *J* = 8.4 Hz, 1H), 7.64 (t, *J* = 7.6 Hz, 1H), 7.60 ((d, *J* = 7.6 Hz, 1H)), 7.47 (t, *J* = 7.2 Hz, 1H), 7.41-7.39 (m, 1H), 7.34 (d, *J* = 7.6 Hz, 1H), 7.27 (t, *J* = 7.6 Hz, 1H), 4.02 (s, 3H), 3.10 (s, 3H), 2.49 (s, 3H);

^{13}C NMR (100 MHz, $CDCl_3$) δ155.1, 152.0, 151.7, 140.2, 134.0, 132.6, 131.0, 130.1, 129.6, 129.3, 129.0, 128.5, 128.2, 124.7, 124.3, 121.5, 115.7, 113.1, 56.5, 31.7, 18.4;

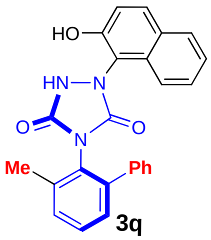

1-(2-hydroxynaphthalen-1-yl)-4-(3-methyl-[1,1'-biphenyl]-2-yl)-1,2,4-triazolidine-3,5-dione (3q) Yield 73%, 95% ee

HPLC analysis [Daicel CHIRALPAK IC column (250 mm × 4.6 mm), hexane/*i*-PrOH =70/30, 1mL/min, T = 25 °C, λ = 230nm, t_R (major) = 8.5 min, t_R (minor) = 23.2 min];

HRMS (ESI) calcd for $C_{25}H_{19}N_3O_3Na^+$ $(M+Na)^+$ 432.1319, found 432.1319;

^{1}H NMR (400 MHz, $CDCl_3$) δ 7.99 (d, *J* = 9.2 Hz, 1H), 7.86 (d, *J* = 8.8 Hz, 2H), 7.59 (t, *J* = 8.0 Hz, 1H), 7.54-7.52 (m, 2H), 7.48-7.39(m, 6H), 7.34-7.30 (m, 2H), 3.76 (s, 3H), 2.89 (s, 3H), 2.49 (s, 3H);

^{13}C NMR (100 MHz, $CDCl_3$) δ 155.8, 153.1, 150.6, 142.8, 139.3, 137.6, 133.3, 132.5, 130.3, 129.7, 129.1, 128.95, 128.9, 128.4, 128.4, 128.1, 128.0, 127.2, 124.6, 121.1, 114.1, 113.7, 56.8, 31.5, 18.2;

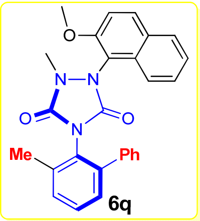

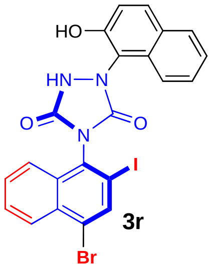

3r

4-(4-bromo-2-iodonaphthalen-1-yl)-1-(2-hydroxynaphthalen-1-yl)-1,2,4-triazolidine-3,5-dione (3r) Yield 62%, 92% ee

HPLC analysis [Daicel CHIRALPAK AD-H column (250 mm × 4.6 mm), hexane/*i*-PrOH = 75/25, 1mL/min, T = 25 °C, λ = 254nm, t_R(minor) = 11.8 min, t_R(major) = 32.0 min];

HRMS (ESI) calcd for $C_{22}H_{13}BrIN_3O_3Na^+$ $(M+Na)^+$ 595.9077, found 595.9075;

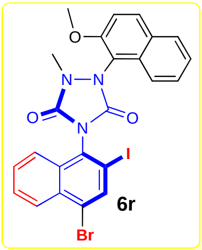

6r

^{1}H NMR (400 MHz, $CDCl_3$) δ 8.36 (s, 1H), 8.32 (d, *J* = 8.0 Hz, 1H), 8.27 (d, *J* = 8.4 Hz, 1H), 8.08 (d, *J* = 9.2 Hz, 1H), 8.04 (d, *J* = 8.0 Hz, 1H), 7.90 (d, *J* = 8.0 Hz, 1H), 7.74-7.65 (m, 3H), 7.49 (t, *J* = 7.6 Hz, 1H), 7.45 (d, *J* = 9.2 Hz, 1H), 4.15 (s, 3H), 3.17 (s, 3H);

^{13}C NMR (100 MHz, $CDCl_3$) δ 155.0, 151.7, 138.2, 134.1, 132.8, 132.7, 132.6, 131.0, 129.1, 128.9, 128.6, 128.4, 128.2, 128.2, 128.1, 125.7, 124.8, 123.4, 121.5, 115.9, 113.2, 98.2, 56.8, 31.7;

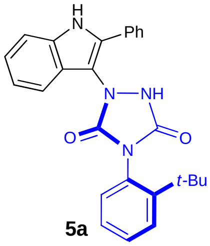

5a

4-(2-(*tert*-butyl)phenyl)-1-(2-phenyl-1H-indol-3-yl)-1,2,4-triazolidine-3,5-dione (5a) Yield 96%, 97% ee

HPLC analysis [Daicel CHIRALPAK IC column (250 mm × 4.6 mm), hexane/*i*-PrOH = 80/20, 1.0 mL/min, T = 25 °C, λ = 230nm, t_R(minor) = 16.4 min, t_R(major) = 25.2 min];

HRMS (ESI) calcd for $C_{26}H_{24}N_4O_2Na^+$ $(M+Na)^+$ 447.1791, found 447.1791;

^{1}H NMR (400 MHz, Acetone-d_6) δ 10.97 (s, 1H), 9.80 (brs, 1H), 7.94-7.92 (m, 2H), 7.70 (dd, *J* = 8.0, 1.2 Hz, 1H), 7.69 (d, *J* = 7.6 Hz, 1H), 7.57-7.45 (m, 5H), 7.39 (td, *J* = 7.6, 1.6 Hz, 1H), 7.32 (dd, *J* = 8.0, 1.6 Hz, 1H), 7.27 (td, *J* = 7.2, 1.2 Hz, 1H), 7.20 (td, *J* = 7.6, 0.8 Hz, 1H), 1.49 (s, 9H);

^{13}C NMR (100 MHz, Acetone-d_6) δ 153.9, 153.2, 149.2, 136.8, 134.9, 132.2, 130.6, 130.3, 129.8, 128.9, 128.6, 128.5, 127.6, 127.2, 125.5, 123.0, 120.6, 117.9, 112.0, 108.1, 35.6, 31.2;

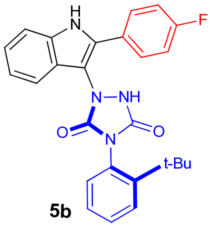

5b

4-(2-(*tert*-butyl)phenyl)-1-(2-(4-fluorophenyl)-1H-indol-3-yl)-1,2,4-triazolidine-3,5-dione (5b) Yield 95%, 96% ee

HPLC analysis [Daicel CHIRALPAK IC column (250 mm × 4.6 mm), hexane/*i*-PrOH = 80/20, 1.0 mL/min, T = 25 °C, λ = 230nm, t_R(minor) = 9.3 min, t_R(major) = 15.8 min];

HRMS (ESI) calcd for $C_{26}H_{24}FN_4O_2^+$ $(M+H)^+$ 443.1878, found 443.1879;

^{1}H NMR (400 MHz, Acetone-d_6) δ 10.98 (s, 1H), 9.71 (brs, 1H), 7.99-7.95 (m, 2H), 7.70 (dd, *J* = 8.0, 1.6 Hz, 1H), 7.69 (d, *J* = 7.6 Hz, 1H), 7.52 (d, *J* = 8.4 Hz, 1H), 7.49 (td, *J* = 8.0, 1.6 Hz, 1H), 7.39 (td, *J* = 7.2, 1.2 Hz, 1H), 7.35-7.29 (m, 3H), 7.27 (td, *J* = 7.2, 1.2 Hz, 1H), 7.21 (td, *J* = 7.6, 1.2 Hz, 1H), 1.49 (s, 9H);

^{13}C NMR (100 MHz, Acetone-d_6) δ 162.8 (d, $J_{C\text{-}F}$ = 246 Hz), 153.9, 153.3, 149.2, 135.9, 134.9, 132.2, 130.2, 129.8, 129.7 (d, $^3J_{C\text{-}F}$ = 8.4 Hz), 128.6, 127.2, 127.0 (d, $^4J_{C\text{-}F}$ = 3.3 Hz), 125.3, 123.0, 120.7, 117.9, 115.8 (d, $^2J_{C\text{-}F}$ = 21.8 Hz), 112.0, 108.0, 35.6, 31.2;

^{19}F NMR (376 MHz, Acetone-d_6) δ -114.06 (s, 1F);

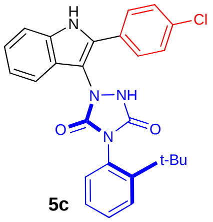

5c

4-(2-(*tert*-butyl)phenyl)-1-(2-(4-chlorophenyl)-1H-indol-3-yl)-1,2,4-triazolidine-3,5-dione (**5c**) Yield 95%, 92% ee

HPLC analysis [Daicel CHIRALPAK IC column (250 mm × 4.6 mm), hexane/*i*-PrOH = 80/20, 1.0 mL/min, T = 25 °C, λ = 240nm, t_R(minor) = 9.1 min, t_R(major) = 16.4 min];

HRMS (ESI) calcd for $C_{26}H_{23}ClN_4O_2Na^+$ $(M+Na)^+$ 481.1402, found 481.1403;

^{1}H NMR (400 MHz, Acetone-d_6) δ 11.04 (s, 1H), 9.65 (brs, 1H), 7.95-7.92 (m, 2H), 7.71-7.70 (m, 2H), 7.60-7.56 (m, 2H), 7.53 (d, *J* = 8.0 Hz, 1H), 7.50 (td, *J* = 8.0, 1.6 Hz, 1H), 7.39 (td, *J* = 8.0, 1.6 Hz, 1H), 7.33 (td, *J* = 8.0, 1.6 Hz, 1H), 7.28 (td, *J* = 7.6, 0.8 Hz, 1H), 7.21 (td, *J* = 7.6, 0.8 Hz, 1H), 1.49 (s, 9H);

^{13}C NMR (100 MHz, Acetone-d_6) δ 153.9, 153.3, 149.2, 135.4, 135.0, 134.0, 132.2, 130.2, 129.8, 129.4, 129.1, 129.0, 128.5, 127.2, 125.3, 123.2, 120.8, 118.0, 112.0, 108.6, 35.6, 31.2;

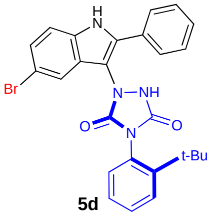

5d

1-(5-bromo-2-phenyl-1H-indol-3-yl)-4-(2-(*tert*-butyl)phenyl)-1,2,4-triazolidine-3,5-dione (**5d**) Yield 94%, 94% ee

HPLC analysis [Daicel CHIRALPAK IC column (250 mm × 4.6 mm), hexane/*i*-PrOH = 85/15, 1.0 mL/min, T = 25 °C, λ = 254nm, t_R(minor) = 10.8 min, t_R(major) = 15.0 min];

HRMS (ESI) calcd for $C_{26}H_{23}BrN_4O_2Na^+$ $(M+Na)^+$ 525.0897, found 525.0899;

^{1}H NMR (400 MHz, Acetone-d_6) δ 11.17 (s, 1H), 9.82 (brs, 1H), 7.93-7.89 (m, 3H), 7.70 (dd, *J* = 8.4, 1.2 Hz, 1H), 7.56 (t, *J* = 8.0 Hz, 2H), 7.51-7.46 (m, 3H), 7.41-7.34 (m, 3H), 1.49 (s, 9H);

^{13}C NMR (100 MHz, Acetone-d_6) δ 154.0 , 153.1 , 149.2 , 138.4 , 133.5 , 132.3 , 130.2 , 130.0 , 129.8 , 129.0 , 129.0 , 128.5 , 127.7 , 127.2 , 127.2 , 125.6 , 120.4 , 113.9 , 113.5 , 107.4 , 35.6 , 31.2;

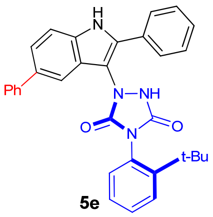

5e

4-(2-(*tert*-butyl)phenyl)-1-(2,5-diphenyl-1H-indol-3-yl)-1,2,4-triazolidine-3,5-dione (**5e**) Yield 90%, 96% ee

HPLC analysis [Daicel CHIRALPAK IC column (250 mm × 4.6 mm), hexane/*i*-PrOH = 85/15, 1.0 mL/min, T = 25 °C, λ = 254nm, t_R(major) = 16.6 min, t_R(minor) = 19.7 min];

HRMS (ESI) calcd for$C_{32}H_{28}N_4O_2Na^+$ $(M+Na)^+$ 523.2104, found 523.2105;

^{1}H NMR (400 MHz, Acetone-d_6) δ 11.08 (s, 1H), 9.81 (s, 1H), 7.96 (d, *J* = 8.8 Hz, 2H), 7.95 (s, 1H) 7.74 (d, *J* = 7.6 Hz, 2H), 7.70 (d, *J* = 8.4 Hz, 1H), 7.84-7.55 (m, 4H), 7.51 (t, *J* = 8.0 Hz, 2H), 7.49 (t, *J* = 8.0 Hz, 2H), 7.41-7.36 (m, 3H), 1.51 (s, 9H);

^{13}C NMR (100 MHz, Acetone-d_6) δ 154.0, 153.1, 149.2, 142.0, 137.6, 134.5, 134.1, 132.2, 130.5, 130.4, 129.7, 128.9, 128.8, 128.7,128.5, 127.6, 127.2, 127.1, 126.6, 126.1, 122.6, 116.1, 112.4, 108.4, 35.6, 31.2;

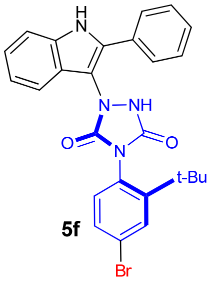

5f

4-(4-bromo-2-(*tert*-butyl)phenyl)-1-(2-phenyl-1H-indol-3-yl)-1,2,4-triazolidine-3,5-dione (5f) Yield 92%, 93% ee

HPLC analysis [Daicel CHIRALPAK IC column (250 mm × 4.6 mm), hexane/*i*-PrOH = 80/20, 1.0 mL/min, T = 25 °C, λ = 230nm, t_R(minor) = 13.5 min, t_R(major) = 22.9 min];

HRMS (ESI) calcd for $C_{26}H_{23}BrN_4O_2Na^+$ $(M+Na)^+$ 525.0897, found 525.0892;

^{1}H NMR (400 MHz, Acetone-d_6) δ 11.04 (s, 1H), 7.91 (d, *J* = 8.0 Hz, 2H), 7.84 (s, 1H), 7.69 (d, *J* = 8.0 Hz, 1H), 7.59 (d, *J* = 8.4 Hz, 1H), 7.56-7.51 (m, 3H), 7.46 (t, *J* = 7.2 Hz, 1H), 7.33 (d, *J* = 8.4 Hz, 1H), 7.27 (t, *J* = 7.6 Hz, 1H), 7.20 (t, *J* = 7.6 Hz, 1H), 1.49 (s, 9H);

^{13}C NMR (100 MHz, Acetone-d_6) δ 153.4, 152.8, 152.0, 136.9, 134.9, 134.3, 131.6, 130.5, 130.4, 129.8, 128.9, 128.7, 127.6, 125.5, 123.5, 123.0, 120.7, 117.9, 112.0, 107.8, 35.8, 30.9;

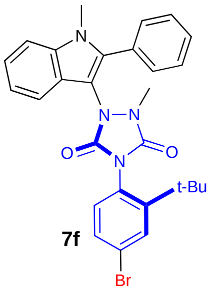

7f

4-(4-bromo-2-(*tert*-butyl)phenyl)-1-methyl-2-(1-methyl-2-phenyl-1H-indol-3-yl)-1,2,4-triazolidine-3,5-dione (7f) Yield 93%, 93% ee

HPLC analysis [Daicel CHIRALPAK ID column (250 mm × 4.6 mm), hexane/*i*-PrOH = 60/40, 1.0 mL/min, T = 25 °C, λ = 230nm, t_R(minor) = 17.8 min, t_R(major) = 25.7 min];

^{1}H NMR (400 MHz, CD_3CN) δ 7.81 (dd, J = 7.2, 2.4 Hz, 1H), 7.72 (d, J = 8.0 Hz, 0.6H), 7.65-7.50 (m, 7.4H), 7.40 (td, J = 7.2, 1.6 Hz, 1H), 7.32-7.28 (m, 1.6H), 6.88 (d, J = 8.4 Hz, 0.4H), 3.74 (s, 1.7H), 3.73 (s, 1.3H), 2.90 (s, 3H), 1.45 (s, 4H), 1.24 (s, 5H);

^{13}C NMR (100 MHz, CD_3CN) δ 154.2, 154.1, 153.9, 152.9, 152.8, 152.8, 142.9, 142.6, 137.0, 136.9, 134.8, 134.6, 132.8, 132.7, 131.5, 131.2, 131.2, 130.5, 130.5, 130.3, 130.3, 130.1, 129.8, 129.7, 129.7, 125.8, 125.3, 124.7, 123.9, 123.9, 122.2, 122.1, 117.9, 111.7, 111.7, 107.2, 106.1, 36.7, 36.6, 32.3, 32.1, 32.0, 31.9, 31.7, 31.5;

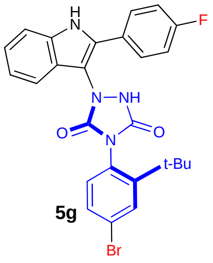

5g

4-(4-bromo-2-(*tert*-butyl)phenyl)-1-(2-(4-fluorophenyl)-1H-indol-3-yl)-1,2,4-triazolidine-3,5-dione (5g) Yield 92%, 93% ee

HPLC analysis [Daicel CHIRALPAK IC column (250 mm × 4.6 mm), hexane/*i*-PrOH = 80/20, 1.0 mL/min, T = 25 °C, λ = 230nm, t_R(minor) = 8.5 min, t_R(major) = 15.8 min];

HRMS (ESI) calcd for $C_{26}H_{22}BrFN_4O_2Na^+$ $(M+Na)^+$ 543.0802, found 543.0801;

^{1}H NMR (400 MHz, Acetone-d_6) δ 11.01 (s, 1H), 9.79 (brs, 1H), 7.97-7.84 (m, 2H), 7.83 (d, *J* = 2.0 Hz, 1H), 7.69 (d, *J* = 8.0 Hz, 1H), 7.59 (dd, *J* = 8.0, 2.0 Hz, 1H), 7.52 (d, *J* = 8.0 Hz, 1H), 7.35 -7.29 (m, 3H), 7.27 (td, *J* = 8.0, 0.8 Hz,1H), 7.20 (td, *J* = 8.0, 0.8 Hz,1H)), 1.49 (s, 9H);

^{13}C NMR (100 MHz, Acetone-d_6) δ 162.9 (d, $J_{C\text{-}F}$ = 245.7 Hz), 153.4, 152.8, 151.9, 135.9, 134.9 , 134.3, 131.7, 130.4, 129.7 (d, $^3J_{C\text{-}F}$ = 8.2 Hz), 129.7, 126.9 (d, $^4J_{C\text{-}F}$ = 3.3 Hz), 125.3, 123.6, 123.0 ,

120.7, 117.9, 115.8 (d, $^2J_{C\text{-}F}$ = 21.6 Hz), 112.0, 107.8, 35.8, 30.9 ;
^{19}F NMR (376 MHz, Acetone-D6) δ -113.97 (s, 1F);

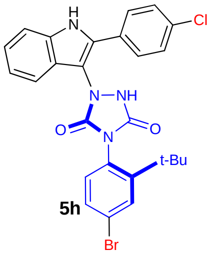
5h

4-(4-bromo-2-(*tert*-butyl)phenyl)-1-(2-(4-chlorophenyl)-1H-indol-3-yl)-1,2,4-triazolidine-3,5-dione (**5h**) Yield 93%, 91% ee
HPLC analysis [Daicel CHIRALPAK IC column (250 mm × 4.6 mm), hexane/*i*-PrOH = 80/20, 1.0 mL/min, T = 25 °C, λ = 230nm, t_R(minor) =8.4 min, t_R(major) = 16.3 min];
HRMS (ESI) calcd for $C_{26}H_{22}BrClN_4O_2Na^+$ $(M+Na)^+$ 559.0507, found 559.0500;

^{1}H NMR (400 MHz, Acetone-d_6) δ 11.07 (s, 1H), 9.72 (brs, 1H), 7.93 (d, *J* = 8.4 Hz, 2H), 7.84 (d, *J* = 2.4 Hz, 1H), 7.70 (d, *J* = 7.6 Hz, 1H), 7.61-7.56 (m, 3H), 7.52 (d, *J* = 8.0 Hz, 1H), 7.35 (d, *J* = 8.4 Hz, 1H), 7.28 (t, *J* = 8.0 Hz, 1H), 7.21 (t, *J* = 7.6 Hz, 1H), 1.49 (s, 9H);
^{13}C NMR (100 MHz, Acetone-d_6) δ 153.4, 152.8, 151.9, 135.5, 135.0, 134.3, 134.0, 131.7, 130.4, 129.6, 129.2, 129.1, 129.0, 125.2, 123.6, 123.3, 120.8, 118.0, 112.1, 108.1, 35.8, 30.9;

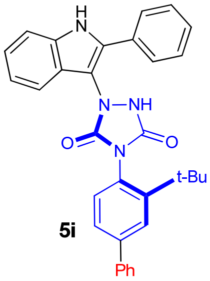
5i

4-(3-(*tert*-butyl)-[1,1'-biphenyl]-4-yl)-1-(2-phenyl-1H-indol-3-yl)-1,2,4-triazolidine-3,5-dione (**5i**) Yield 92%, 94% ee
HPLC analysis [Daicel CHIRALPAK IC column (250 mm × 4.6 mm), hexane/*i*-PrOH = 80/20, 1.0 mL/min, T = 25 °C, λ = 230nm, t_R(minor) = 9.8 min, t_R(major) = 25.9 min];
HRMS (ESI) calcd for $C_{32}H_{28}N_4O_2Na^+$ $(M+Na)^+$ 523.2104, found 523.2103;

^{1}H NMR (400 MHz, Acetone-d_6) δ 10.99 (s, 1H), 9.78 (brs, 1H), 7.96-7.94 (m, 2H), 7.92 (d, *J* = 2.0 Hz, 1H), 7.75-7.71 (m, 3H), 7.65 (dd, *J* = 8.0, 2.0 Hz, 1H), 7.58-7.52 (m, 5H), 7.50-7.41 (m, 3H), 7.28 (td, *J* = 7.2, 0.8 Hz, 1H), 7.22 (td, *J* = 7.2, 0.8 Hz, 1H), 1.56 (s, 9H);
^{13}C NMR (100 MHz, Acetone-d_6) δ 153.8, 153.2, 149.7, 142.4, 140.6, 136.8, 134.9, 132.8, 130.6, 129.6, 128.9, 128.6, 127.7, 127.6, 127.3, 127.2, 125.8, 125.5, 123.0, 120.6, 117.9, 112.0, 108.1, 35.8, 31.2;

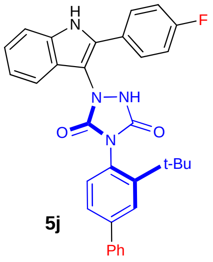
5j

4-(3-(*tert*-butyl)-[1,1'-biphenyl]-4-yl)-1-(2-(4-fluorophenyl)-1H-indol-3-yl)-1,2,4-triazolidine-3,5-dione (**5j**) Yield 91%, 95% ee
HPLC analysis HPLC analysis [Daicel CHIRALPAK IC column (250 mm × 4.6 mm), hexane/*i*-PrOH = 80/20, 1.0 mL/min, T = 25 °C, λ = 230nm, t_R(minor) = 11.0 min, t_R(major) = 33.8 min];
HRMS (ESI) calcd for $C_{32}H_{27}FN_4O_2Na^+$ $(M+Na)^+$ 541.2010, found 541.2010;
^{1}H NMR (400 MHz, Acetone-d_6) δ 11.00 (s, 1H), 9.75 (brs, 1H), 8.01-7.96 (m, 2H), 7.92 (d, *J* = 2.0 Hz, 1H), 7.75-7.71 (m, 3H), 7.65 (dd, *J* = 8.1, 2.0 Hz, 1H), 7.52 (t, *J* = 7.2 Hz, 3H), 7.44-7.41 (m, 2H), 7.34 (t, *J* = 8.8 Hz, 2H), 7.28 (td, *J* = 6.8, 1.2 Hz,1H), 7.22 (td, *J* = 7.2 1.2 Hz,1H), 1.56 (s, 9H);
^{13}C NMR (100 MHz, Acetone-d_6) δ 162.9 (d, $J_{C\text{-}F}$ = 245.5 Hz), 153.9, 153.3, 149.6, 142.5, 140.5,

135.9, 134.9, 132.7, 129.8 (d, ${}^3J_{C-F}$ = 8.2 Hz), 129.5, 128.9, 127.7, 127.3, 127.2, 127.0 (d, ${}^4J_{C-F}$ = 3.2 Hz), 125.8, 125.4, 123.0, 120.7, 118.0, 115.8 (d, ${}^2J_{C-F}$ = 21.8 Hz), 112.0, 108.1, 35.8, 31.2;
^{19}F NMR (376 MHz, Acetone-D6) δ -114.06 (s, 1F);

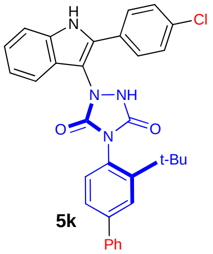

5k

4-(3-(*tert*-butyl)-[1,1'-biphenyl]-4-yl)-1-(2-(4-chlorophenyl)-1H-indol-3-yl)-1,2,4-triazolidine-3,5-dione (5k) Yield 86%, 92% ee

HPLC analysis HPLC analysis [Daicel CHIRALPAK IC column (250 mm × 4.6 mm), hexane/*i*-PrOH = 80/20, 1.0 mL/min, T = 25 °C, λ = 230nm, t_R(minor) = 8.4 min, t_R(major) = 25.7 min];

HRMS (ESI) calcd for $C_{32}H_{27}ClN_4O_2Na^+$ $(M+Na)^+$ 557.1715, found 557.1714;

^{1}H NMR (400 MHz, Acetone-d_6) δ 11.05 (s, 1H), 9.80 (brs, 1H), 7.96 (d, *J* = 8.8 Hz, 2H), 7.93 (d, *J* = 2.0 Hz, 1H), 7.76-7.72 (m, 3H), 7.65 (dd, *J* = 8.0, 2.0 Hz, 1H), 7.59 (d, *J* = 8.8 Hz, 2H), 7.55 -7.50 (m, 3H), 7.45-7.41 (m, 2H), 7.29 (td, *J* = 7.2, 1.2 Hz, 1H), 7.22 (t, *J* = 7.2 Hz, 1H), 1.56 (s, 9H);
^{13}C NMR (100 MHz, Acetone-d_6) δ 153.9, 153.3, 149.6, 142.5, 140.5, 135.4, 135.0, 134.0, 132.8, 129.4, 129.3, 129.1, 129.0, 128.9, 127.8, 127.3, 127.3, 125.8, 125.3, 123.2, 120.8, 118.0, 112.1, 108.5, 35.8, 31.2;

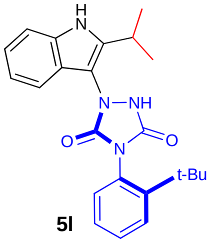

5l

4-(2-(*tert*-butyl)phenyl)-1-(2-isopropyl-1H-indol-3-yl)-1,2,4-triazolidine-3,5-dione (5l) Yield 95%, 90% ee

HPLC analysis HPLC analysis [Daicel CHIRALPAK IC column (250 mm × 4.6 mm), hexane/*i*-PrOH = 80/20, 1.0 mL/min, T = 25 °C, λ = 230nm, t_R(minor) = 7.5 min, t_R(major) =13.1 min];

HRMS (ESI) calcd for $C_{23}H_{26}N_4O_2Na^+$ $(M+Na)^+$ 413.1948, found 413.1945;

^{1}H NMR (400 MHz, Acetone-d_6) δ 10.50 (brs, 1H), 9.69 (brs, 1H), 7.69 (dd, *J* = 8.0, 1.6 Hz, 1H), 7.55 (d, *J* = 7.2 Hz, 1H), 7.48 (td, *J* = 7.2, 1.6 Hz, 1H), 7.38 (td, *J* = 7.6, 1.2 Hz, 2H), 7.30 (dd, *J* = 7.6, 1.6 Hz, 1H), 7.17-7.09 (m, 2H), 3.47-3.37 (m, 1H), 1.49 (s, 9H), 1.43 (d, *J* = 3.2 Hz, 3H), 1.41 (d, *J* = 2.8 Hz, 3H);
^{13}C NMR (100 MHz, Acetone-d_6) δ 154.0, 152.8, 149.2, 144.7, 134.4, 132.2, 130.4, 129.7, 128.5, 127.1, 124.8, 121.6, 120.0, 117.1, 111.5, 106.5, 35.6, 31.2, 25.6, 21.5;

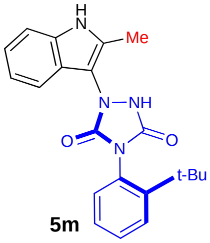

5m

4-(2-(*tert*-butyl)phenyl)-1-(2-methyl-1H-indol-3-yl)-1,2,4-triazolidine-3,5-dione (5m) Yield 95%, 84% ee

HPLC analysis HPLC analysis [Daicel CHIRALPAK IC column (250 mm × 4.6 mm), hexane/*i*-PrOH = 70/30, 1.0 mL/min, T = 25 °C, λ = 230nm, t_R(minor) = 6.6 min, t_R(major) = 16.2 min];

HRMS (ESI) calcd for $C_{21}H_{22}N_4O_2Na^+$ $(M+Na)^+$ 385.1635, found 385.1635;

^{1}H NMR (400 MHz, Acetone-d_6) δ 10.44 (s, 1H), 9.67 (brs, 1H), 7.69 (d, *J* = 8.0 Hz, 1H), 7.54 (d,

J = 7.6 Hz, 1H), 7.47 (td, *J* = 8.0, 1.6 Hz, 1H), 7.40-7.36 (m, 2H), 7.30 (dd, *J* = 7.6, 1.6 Hz, 1H), 7.16-7.09 (m, 2H), 2.48 (s, 3H), 1.49 (s, 9H);
^{13}C NMR (100 MHz, Acetone-d_6) δ 154.1, 152.7, 149.2, 135.0, 134.3, 132.2, 130.4, 129.6, 128.5, 127.1, 124.7, 121.5, 120.0, 117.0, 111.3, 108.6, 35.5, 31.2, 10.2.

Supplementary References

1. Hanhan, N. V., Sahin, A. H., Chang, T. W., Fettinger, J. C. & Franz, A. K. Catalytic asymmetric synthesis of substituted 3-hydroxy-2-oxindoles. *Angew. Chem., Int. Ed.* **49**, 744-747 (2010).
2. Charalambides, Y. C., & Moratti, S. C. Comparison of base-promoted and self-catalyzed conditions in the synthesis of isocyanates from amines using triphosgene. *Syn. Commun.* **37**, 1037–1044 (2007).
3. Zhu, M.-Y., Kim, M. H., Lee, S., Bae, S. J., Kim, S. H. & Park. S. B. Discovery of novel benzopyranyl tetracycles that act as inhibitors of Osteoclastogenesis induced by receptor activator of NF-κB ligand. *J. Med. Chem.* **53**, 8760–8764 (2010).
4. Cookson, R. C., Gupte, S. S., Stevens, R, I. D. & Watts, C. T. 4-phenyl-1,2,4-triazoline-3,5-dione. *Org. Synth.* **6**, 936–940 (1988).
